# Knowledge-guided gene ranking by coordinative component analysis

**DOI:** 10.1186/1471-2105-11-162

**Published:** 2010-03-30

**Authors:** Chen Wang, Jianhua Xuan, Huai Li, Yue Wang, Ming Zhan, Eric P Hoffman, Robert Clarke

**Affiliations:** 1 Department of Electrical and Computer Engineering, Virginia Polytechnic Institute and State University, Arlington, VA, USA; 2 Bioinformatics Unit, Research Resources Branch, National Institute on Aging, NIH, Baltimore, MD, USA; 3 Research Center for Genetic Medicine, Children's National Medical Center, Washington, DC, USA; 4 Departments of Oncology and Physiology & Biophysics, Georgetown University School of Medicine, Washington, DC, USA

## Abstract

**Background:**

In cancer, gene networks and pathways often exhibit dynamic behavior, particularly during the process of carcinogenesis. Thus, it is important to prioritize those genes that are strongly associated with the functionality of a network. Traditional statistical methods are often inept to identify biologically relevant member genes, motivating researchers to incorporate biological knowledge into gene ranking methods. However, current integration strategies are often heuristic and fail to incorporate fully the true interplay between biological knowledge and gene expression data.

**Results:**

To improve knowledge-guided gene ranking, we propose a novel method called coordinative component analysis (COCA) in this paper. COCA explicitly captures those genes within a specific biological context that are likely to be expressed in a coordinative manner. Formulated as an optimization problem to maximize the coordinative effort, COCA is designed to first extract the coordinative components based on a partial guidance from knowledge genes and then rank the genes according to their participation strengths. An embedded bootstrapping procedure is implemented to improve statistical robustness of the solutions. COCA was initially tested on simulation data and then on published gene expression microarray data to demonstrate its improved performance as compared to traditional statistical methods. Finally, the COCA approach has been applied to stem cell data to identify biologically relevant genes in signaling pathways. As a result, the COCA approach uncovers novel pathway members that may shed light into the pathway deregulation in cancers.

**Conclusion:**

We have developed a new integrative strategy to combine biological knowledge and microarray data for gene ranking. The method utilizes knowledge genes for a guidance to first extract coordinative components, and then rank the genes according to their contribution related to a network or pathway. The experimental results show that such a knowledge-guided strategy can provide context-specific gene ranking with an improved performance in pathway member identification.

## Background

It is of great interest to identify genes strongly associated with the functionality of gene networks or signal transduction pathways particularly from gene expression microarray data. Two of the earliest approaches to identify such genes are fold-change and multiple t-testing; each aims to rank the genes in the order of their differential expressions under various experimental conditions. Many improvements to the original t-test method have been proposed for microarray data analysis. For example, significant analysis of microarray (SAM) [1] uses a modified t-statistic with an added estimator for gene ranking in which the false discovery rate (FDR) is estimated by a permutation procedure. A bootstrapped p-value approach was introduced in [2] to address the inherent variability in small sample studies. Prior studies have shown that fold-change is more robust than t-test with respect to the reproducibility of gene rankings [3], while other researchers argue that better reproducibility does not guarantee the accuracy of gene ranking[4]. Nonetheless, both methods are severely limited because they neglect the interaction among genes, prioritizing gene relevance only based on individual gene expression values.

To address the above-mentioned problem, several gene ranking methods have been proposed to either consider the joint effect of genes or to explore the expression pattern in time-course data. For instance, Opgen-Rhein & Strimmer [5] introduced the "shrinkage t" statistic that is based on a novel and model-free shrinkage estimate of the variance vector across genes. Storey *et al. *[6] proposed a method (EDGE) to first fit the time-course expression pattern by splines, and then rank genes by hypothesis testing on the spline parameters. Furlanello *et al. *[7] proposed a classification-based feature elimination scheme to rank genes by iteratively discarding chunks of genes showing least contribution to the classifier.

In contrast, other investigators have proposed incorporating biological knowledge for gene ranking. GeneRank [8] ranks genes by integrating gene expression and network structure derived from gene annotations. Ma *et al. *[9] proposed a strategy to combine gene expression and protein-protein interaction (PPI) knowledge, ranking genes by their association with phenotype calibrated by the PPI information. However, such data integration, while widely adopted, is usually done in a heuristic way and lacks an objective estimate of the true interplay between biological knowledge and gene expression data.

In this paper, we propose a knowledge-guided gene ranking scheme, namely a coordinative component analysis (COCA) algorithm, to model explicitly those genes that are most likely to be expressed in a coordinative manner within a specific biological context. We consider the genes that belong to a pathway or a network as a whole, rather than treating genes as independent or individual measures. To enhance the biological relevance of gene ranking, gene organization requires that the intrinsic coordination among the genes be defined by biological knowledge. Specifically, biological knowledge, which could be the gene sets within a biological pathway or sub-network derived from relevant biological databases, is used to guide the algorithm. Thus, we can address the conditional specificity of biological context, for example, where the deregulation of a network only occurs under specific conditions. We rank each individual gene by evaluating its participation or involvement in the pathway of interest, when projected onto the coordinative direction learned by the COCA algorithm. In COCA, a bootstrapping procedure is also implemented to improve the statistical robustness of the ranking results. We demonstrate that the COCA approach can provide an improved performance as compared to traditional statistical methods using simulation data and published gene expression microarray data including yeast cell cycle data and stem cell time-course data, indicating its effectiveness for incorporating biological knowledge into gene ranking.

## Methods

**Figure 1:**
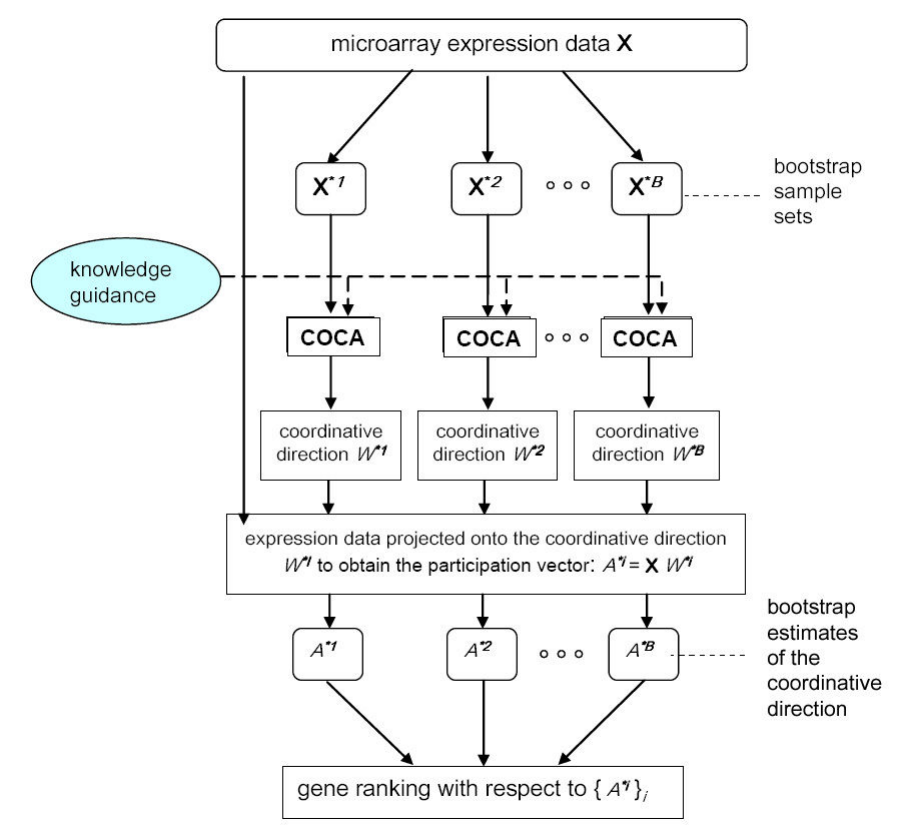
**A flowchart of the proposed approach, namely knowledge-guided coordinative component analysis (COCA), for gene ranking**. A bootstrapping procedure is designed to increase the confidence in estimating the coordinative component (W) and participation vector (A).

A flowchart of the proposed approach is shown in Figure 1. Given a gene expression microarray data set, multiple data sets are first generated through bootstrap resampling of the genes in the array. The bootstrapping procedure is used to overcome the over-fitting problem associated with a small sample size relative to the very high dimensionality of the primary data [10,11]. Each bootstrap sampled data set is then analyzed by the proposed COCA algorithm. COCA aims to learn a coordinative direction by integrating biological knowledge and gene expression data, with which the knowledge is maximally aligned along the coordinative direction. The involvement of each gene in the knowledge network or pathway is estimated from a projection onto the coordinative direction. Finally, multiple bootstrapped estimates of the involvement are merged to create the gene ranking. Note that the COCA software package is made available at the following link: http://www.cbil.ece.vt.edu/software.htm.

### Coordinative component analysis (COCA)

Linear latent variable models are widely used in microarray data analysis, reflecting their simplicity and parsimonious characteristics [12]. In a linear model, gene expressions are represented as the sum of a relatively small number of biological functions (biological processes or signaling pathways or networks) [9,13]:(1)

where **X **∈ ℝ^*N *× *M *^is the mRNA expression matrix consisting of *M *microarray samples with *N *genes. **A **∈ ℝ^*N *× *L *^is the participation or involvement matrix in which each element *a*_*ji *_represents the participation relationship from gene *j *to biological process *i *(i.e., how likely gene *j *is involved in biological process *i*). **T **∈ ℝ^*L *× *M *^contains the latent or hidden activities of biological processes. Given the model as in Eq. (1), several decomposition methods have been proposed to infer **A **and **T **from the mRNA expression profile **X **under certain statistical assumptions [9,14-16] or biological knowledge constraints [17-20]. For example, nonnegative matrix factorization (NMF) imposes the non-negativity constraint on both **A **and **T **for gene module identification [14,15]; independent component analysis (ICA) assumes the independence of biological processes for a sparse decomposition of gene expression [9,16,21]; network component analysis (NCA) incorporates the protein-DNA binding information to constrain the network topology for a reliable estimation of **A **and **T **[17,18]. Despite some apparent success, it remains a difficult task to infer *biologically plausible ***A **and **T **from **X**, mainly due to the complexity of biological systems, the noise in gene expression data **X**, and the incompleteness of current biological knowledge. For example, while the DNA binding of transcript factors (TFs) with high affinity is a more reliable predictor of TF activity than low affinity binding (which are often ignored), studies also showed that low affinity TF-DNA binding can be both evolutionarily and functionally important [22].

In this paper, we address the above-mentioned problem from a different perspective in the context of gene ranking, where network or pathway knowledge is incorporated to guide a COCA approach for inferring the involvement of member genes. In COCA, we apply a linear filtering procedure to extract a particular column of the involvement matrix **A **from **X **by *A*_*i *_= **X***W*_*i*_. As designed, *A*_*i *_∈ ℝ^*N *^denotes a participation vector of the *i*-th biological function (a term that can be referred to as biological process, network or pathway in this paper), and its element *a*_*j *_represents the relationship of biological function *i *to gene *j*. We want to find an optimal *W*_*i *_such that *A*_*i *_is coordinately expressed with the pathway or network knowledge genes. To optimize the linear filter *W*_*i *_for a specific pathway or network, the following cost function is used to fulfill the requirement of achieving maximum coordination of member genes:(2)

where  is the *j*-th row vector of **X**, and subscript *p *refers to the *p*-norm. *W*_*i *_∈ ℝ^*M *^can be interpreted conceptually as the coordinative direction of the *i*-th biological function.

To incorporate prior knowledge in Eq. (2), we define a positive masking vector  for the *i*-th biological function, where  indicating the *j*-th gene is likely to be involved in the *i*-th biological function, and  suggesting otherwise. Conversely,  is a negative masking vector, where  suggests there is no evidence for the *j*-th gene's involvement in the *i*-th biological function, and  suggesting otherwise.

Note that different settings for the parameter *p *in Eq. (2) can lead to different versions of COCA; for example, the norm-2 case (*p *= 2) emphasizes the coordinative behavior of member genes in terms of their energy, while the norm-1 case (*p *= 1) uses their absolute amplitude. From our experiments with microarray data, norm-1 is generally less affected by outliers than norm-2, whereas norm-2 tends to amplify the influence of outliers. Therefore, we use the norm-1 version of Eq. (2) as our default COCA approach. Rewriting Eq. (2) in the norm-1 form, we have the following cost function of a linear projection *W*_*i *_to maximize:(3)

We can maximize the cost function *J*_1_(*W*_*i*_) using a gradient-based learning approach, specifically, by updating *W*_*i *_to follow its gradient direction:(4)

Recall that *W*_*i *_is a vector of size *M *(the number of microarray samples) and let us explicitly denote *W*_*i *_into a vector form as *W*_*i*_(*n*) = [*w*_1*i*_(*n*), ⋯, *w*_*ki*_(*n*), ⋯, *w*_*Mi*_(*n*)]^*T*^.

Then, the gradient of *J*_1_(*W*_*i*_) can be calculated by the following equation:(5)

Since it is mathematically difficult to obtain the analytical form of Eq. (5), we use a simultaneous perturbation technique to approximate the gradient [23]:(6)

In Eq. (6), *c *is a small positive constant controlling the degree of perturbation, and *S*(*n*) = [*s*_1 _(*n*), ⋯, *s*_*k *_(*n*), ⋯, *s*_*M *_(*n*)]^*T *^is a simultaneous perturbation vector. Each element of *S(n) *was draw independently from a binary discrete random distribution taking +1 or -1 for values, with a probability of 0.5 for each value. The gradient form in Eq. (6) is also known as the "stochastic gradient", which is particularly useful when there is no analytical form for the derivative of a cost function. Moreover, when multiple local maxima (or "peak" points) exist in the solution space, the stochastic gradient can help the learning algorithm jump out of these undesirable solution points that may entrap the deterministic gradient.

### Bootstrapping the COCA approach for variability analysis

In practice, the typical size of a knowledge gene set is about a few hundreds, which is much smaller than the number of background genes, which can be several thousands in microarray data. One concern with such an imbalanced comparison is that it will almost inevitably lead to over-fitting. To address this problem, we incorporated a bootstrapping procedure into the COCA approach (see Figure 1). Bootstrapping is a computer-intensive method to generate many 'virtual' samples (called bootstrap samples) by the re-sampling with replacement technique. By applying some estimator on these bootstrap samples, one can calculate a number of statistics of this estimator, such as confidence interval, standard error, etc. Moreover, the averaging of estimations on bootstrap samples can also improve the stability of a model and avoid the over-fitting of the model. This strategy is known as bootstrap aggregating ('bagging') [24] and has been widely used in many machine learning applications such as classification [25] and clustering [26]. Here, we mainly utilize the 'bagging' scheme to reduce the variance of COCA estimation. In practice, the background genes are re-sampled multiple times to form bootstrap samples, each with a comparable size of the knowledge genes. For each bootstrap sample X*^b^, *b *= 1, ⋯, *B*, where *B *is the total number of bootstrapping, COCA was applied to estimate the corresponding coordinative direction W*b, and participation vector A*b = **X**W *b. After ambiguity correction (see Additional file 1: Section S3], for more details), we can obtain 'bagging' aggregated estimations of *W *and *A *using {W*^b^}_*b *= 1, ⋯, *B *_and {A*^b^}_*b *= 1, ⋯, *B*_, respectively. Finally, we used the absolute value of 'bagging' aggregated participation vector to rank genes. The larger the absolute participation value of a gene, the higher the gene was ranked.

## Results

### Simulation data

**Figure 2:**
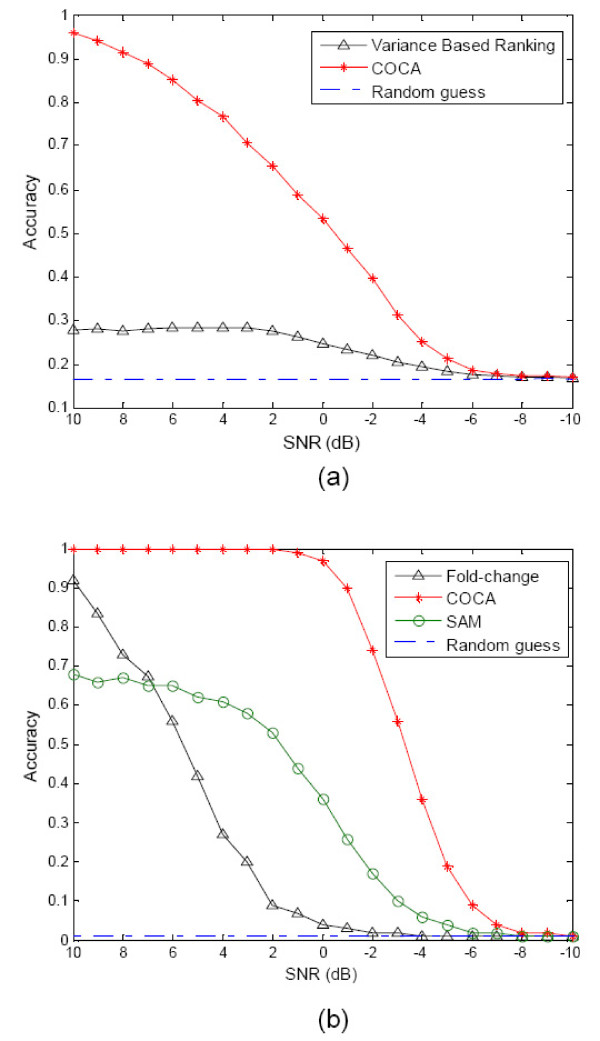
**Performance comparison using simulation data as measured by accuracy vs. signal-to-noise ratio (SNR)**. (a) Comparison of COCA and variance-based ranking (VR) for one-condition case, showing random guess as a baseline. (b) Comparison of COCA, fold-change and SAM for two-condition case, taking random guess as a baseline.

We first applied the proposed COCA approach to simulation data to assess its likely feasibility. Performance of COCA in gene ranking was compared with other methods to demonstrate the improvement. In the simulation of one-condition case, 8 samples were generated according to Eq. (1) with 5 biological processes, each sample consisting of expression measurements of 5,000 genes. For partial knowledge guidance, we input 50 genes to the COCA algorithm, randomly selected from the 200 top ranked genes (called 'ground truth' genes hereafter) of one biological process. In such, COCA incorporated the partial knowledge (from the 50 genes) and set to find the other true knowledge genes (i.e., the remaining 150 'ground truth' genes). We further added a noise component to Eq. (1) to simulate the measurements with different signal-to-noise ratios (SNRs), resulting in a gradual decrease of SNR from 10 dB to -10 dB. Performance of the algorithm was evaluated by its accuracy in finding the genes regulated by the biological process; accuracy is defined as the ratio of the number of 'ground truth' genes identified by the algorithm to the total number of "ground truth" genes, when the genes with the same number as "ground truth" genes were selected for each method. Experimental results from this simulation study are shown in Figure 2(a) that includes a performance comparison with variance-based ranking (VR), an unsupervised method that ranks genes according to their variances. The proposed COCA outperforms VR when SNR is relatively large. When SNR is low (-6 db to -10 db), performance converges to that of a random guess.

Simulations of the two-condition case were also performed. For each condition, 20 samples were generated according to a linear model (Eq. (1)) with 5 biological processes, each sample consisting of expression measurements of 10,000 genes. The difference between the two conditions is that 100 genes, regulated by one biological process in the first condition, were taken out or eliminated in the second condition. Mathematically, let us denote the participation matrices under two conditions as **A**_*cond*1 _= [*A*_1_, *A*_2_, *A*_3_, *A*_4_, *A*_5_] and **A**_*cond*2 _= [, *A*_2_, *A*_3_, *A*_4_, *A*_5_], respectively; except that 100 non-zero items in *A*_1 _were set to be zero in , the items in *A*_1 _are same as those in . Therefore, these 100 'ground-truth' genes are the targets to be detected by the algorithm. For COCA, 50 knowledge genes (not including any of the 100 'ground-truth' genes) are randomly chosen to provide a guidance for the algorithm to find the 100 'ground truth' genes. Similar to the one-condition case, SNR is gradually decreased from 10 dB to -10 dB. Again, performance of the algorithm was evaluated in terms of its accuracy in finding the 'ground-truth' genes; accuracy is defined as the number of detected 'ground-truth' genes among the top ranked 100 genes divided by the total number of 'ground-truth' genes (100 in this case). Figure 2(b) shows the detection accuracies for COCA, fold-change and SAM [1], respectively. COCA outperforms both fold-change and SAM when SNR is higher than -6 dB. For the case of SNR below -6 dB, performances of all three approaches converge to a point that a random guess is equally good. It is worth noting that our COCA approach is designed to detect the changes occurred in the latent level (i.e., the biological process level), while fold-change and SAM approaches are intended to mainly detect the changes in the observation level (i.e., the gene expression level). This major difference can also be appreciated from this simulation study; as seen in Figure 2(b), the performance of COCA remains superior as SNR decreases from 10 dB to 0 dB, while the performance of fold-change or SAM degrades substantially.

### Yeast cell cycle data

We then applied the COCA approach to yeast cell cycle data to identify the genes involved in cell cycle. The yeast cell cycle microarray experiment was performed using fluorescently labeled cDNA arrays, measuring the expression levels of 6178 genes of wild-type *S. cerevisiae *cultures. The cell cycle was synchronized by three independent methods: firstly α-pheromone (α-factor) was used to arrest the cells in G1 phase; secondly centrifugal elutriation was used to obtain small G1 cells; finally, a temperature-sensitive mutation *cdc15-2 *was utilized to arrest cell in mitosis. In our study, we used 59 cDNA samples from these three synchronization experiments [27]. About 800 genes were identified to be periodically expressed during the cell cycle, which can be further grouped into five subsets related to cell cycle phases M/G1, G1, S, G2 and M [27]. In this study, we used these five subsets of genes to further demonstrate the importance of coordinative components in the COCA approach. The total numbers of genes in five subsets (corresponding to M/G1, G1, S, G2 and M) are 113, 120, 196, 300 and 71, respectively. For each phase, 20 genes were randomly selected as knowledge genes to guide the COCA approach. After finding the coordinative component, gene expressions of all genes were projected onto the component for ranking.

**Figure 3:**
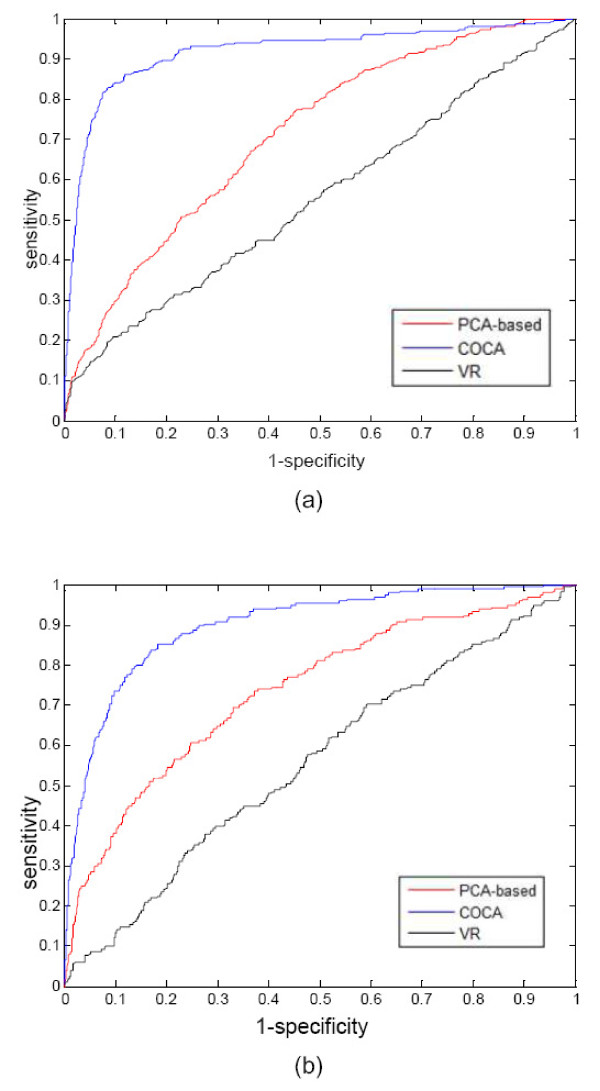
**Receiver Operator Characteristic (ROC) curves of COCA to rank yeast cell cycle-related genes in (a) G1 phase and (b) M phase as synchronized by CDC15**. The ROC curves of other phases can be found in the Additional files [Additional file 1].

To objectively evaluate the performance, receiver operator characteristic (ROC) analysis was conducted to obtain the sensitivity and specificity of the algorithm. Two other approaches were also implemented for a comparison study; the first one is the VR approach that ranks the genes according to their variances; the second one is a supervised approach, which uses principal component analysis (PCA) to first find the principal component of given knowledge genes, and then all the genes are ranked according to their absolute correlations with the principal component. The comparison results are shown in Figure 3 for G1 and M phases; the complete results for all the phases can be found in the supplemental figures [Additional file 1: Figures S1 - S3]. The areas under ROC curves (AUCs) are summarized in Table 1 for all the cell cycle phases under different synchronization methods. Both COCA and PCA-based approaches substantially outperform VR. The VR approach suffers from the lack of knowledge guidance, hence, showing poor performance. More importantly, the COCA approach outperforms the PCA-based approach for all cell cycle phases, since it is the coordinative component (not the principal component) that reflects the underlying regulatory mechanism in yeast cell cycle.

**Table 1:** Performance comparison of COCA, PCA-based and variance-based ranking (VR) approaches.

	Alpha-factor arrest			CDC15 arrest			CDC28 arrest		
	
	COCA	PCA-based	VR	COCA	PCA-based	VR	COCA	PCA-based	VR
M/G1	**0.8477**	0.8277	0.5685	**0.9045**	0.7594	0.5854	**0.7904**	0.7661	0.6524

G2	**0.8182**	0.7172	0.6523	**0.8888**	0.6979	0.5547	**0.8036**	0.6767	0.7418

M	**0.7731**	0.7537	0.6705	**0.8873**	0.7365	0.5572	**0.8448**	0.7585	0.5685

G1	**0.9123**	0.821	0.6611	**0.9172**	0.7119	0.5521	**0.9032**	0.7524	0.792

S	**0.8763**	0.7641	0.7054	**0.9478**	0.6867	0.6385	**0.8532**	0.7058	0.799

Area under ROC curves (AUCs) are summarized in the table for ranking cell cycle-related genes in five yeast cell cycle phases, i.e., M/G1, G1, S, G2 and M, respectively

### Embryonic stem cell data

Understanding the molecular mechanisms controlling self-renewal and differentiation in embryonic stem cells (ESCs) is of central importance towards realizing their potential in medicine and science [28,29]. ESCs serve as a model system for studying cell development and have considerable potential in cancer research and for improving cancer treatments. Most studies on ESC transcriptomes have primarily used fold changes of individual genes to identify the molecular signatures of ESCs for elucidating the mechanisms controlling pluripotency [30-32]. Here, we used the COCA algorithm to infer biologically relevant genes in ESC-critical pathways including Notch, JAK/STAT, TGFβ and WNT pathways [31].

The mouse embryonic stem cell data sets that we used were acquired from [33]. The original research aimed to study the genetic determinants of mouse embryonic stem cell (mESC) differentiation. The transition from mESC to embryoid body (EB) was initialized by removing leukemia inhibitory factor (LIF) and making murine embryonic feeder cells absent. The data that we used was measured on R1 cell line at 11-point time series over a period of two weeks (0 h - undifferentiated mESCs, 6 h, 12 h, 18 h, 24 h, 36 h, 48 h, 4 d, 7 d, 9 d, and 14 d), with three replicates at each time point (GEO database accession number: GSE2972). In our study, we only used 33 samples measured by Affymetrix MOE430A GeneChip set, because the MOE430A array measures genes that are generally better characterized than those on MOE430B and has much better signal quality than MOE430B in terms of false discovery rate of significantly changed probe sets [33].

In the study, 5,000 genes were randomly sampled as the background genes for bootstrapping, and one hundred bootstrap iterations were carried out to estimate the variability and then to perform gene ranking. For the COCA approach, pathway related genes were selected as knowledge genes to guide finding the coordinative component. After finding the coordinative component, gene expressions of all the genes were projected onto the component for ranking.

**Figure 4:**
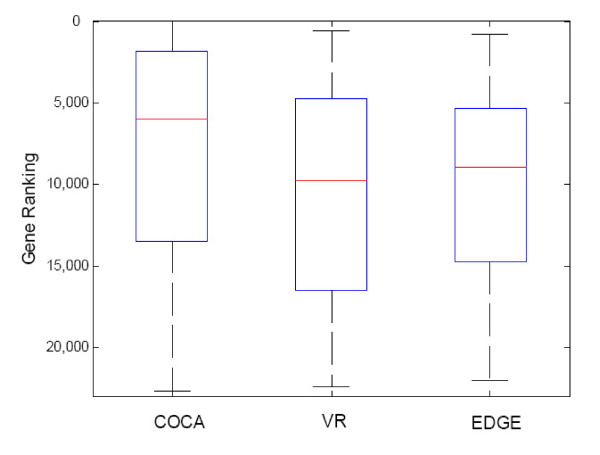
**A boxplot of the ranking of Notch pathway probe sets by Notch pathway-guided COCA, as compared to those by variance-based ranking (VR) and EDGE-based ranking, respectively**.

**Figure 5:**
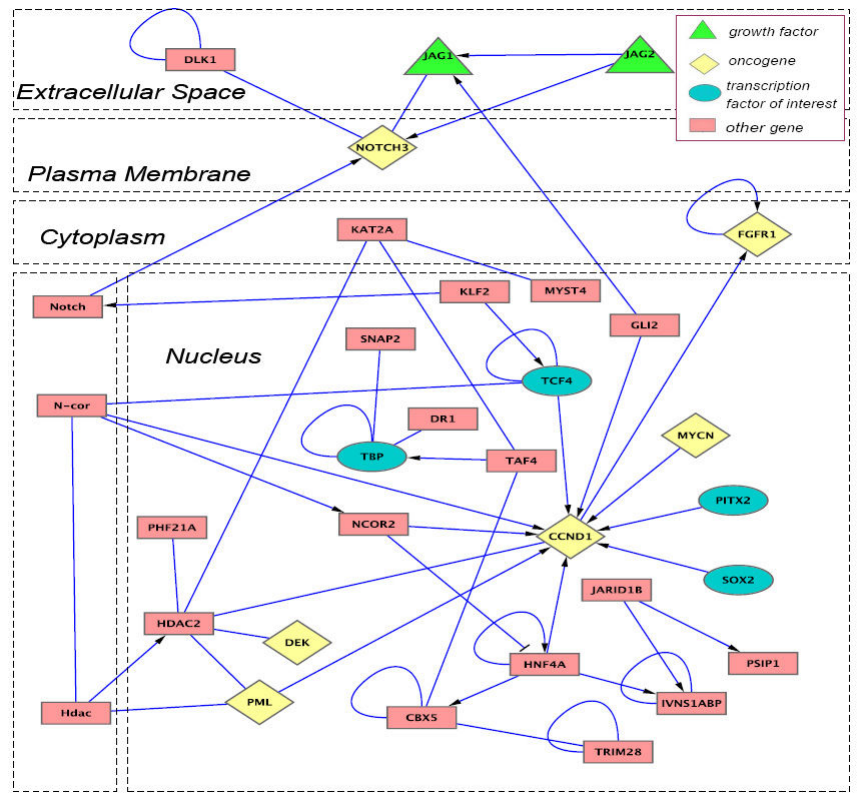
**The identified Notch pathway including several growth factors, transcription factors and oncogenes**. Some of the members (e.g., NOTCH3, JAG1, JAG2 and SOX2) are known to be associated with the Notch pathway while several novel members are revealed by the COCA approach, e.g., transcription factors: TCF4, TBP and PITX2; oncogenes: MYCN, FGFR1 and CCND1.

For each pathway analysis, we generated a gene list of top 500 probe sets ranked by COCA, and conducted pathway and functional enrichment analysis using DAVID [34]http://david.abcc.ncifcrf.gov/. The results of GO enrichment analysis are listed in Table 2 for the Notch pathway; the results of enrichment analysis of other pathways (i.e., JAK/STAT, TGFβ and WNT pathways) and the detailed gene lists can be found in the Supplemental Tables S1 [Additional file 1], S3 - S6 [Additional files 2, 3, 4 and 5]. Taking the results of Notch pathway as an example, we can see from Figure 4 that COCA effectively boosts the ranking of pathway-relating gene set, as compared to conventional approaches like VR and the EDGE [6]. Once the coordinative direction is estimated, we can discover weakly expressed but related genes. While it is well known that many downstream genes have large variation, COCA can boost the ranking of genes with smaller variation but larger participation value. From pathway enrichment analysis, we can see that VR mainly prioritizes ribosome, cell adhesion and metabolic pathways (Table S7), which are more likely the downstream of stem cell development. The EDGE-based ranking prioritizes the pathways related to cell communication, focal adhesion and ECM-receptor interaction (Table S8). On the other hand, COCA-based ranking prioritizes many upstream pathways (Table 2), especially several signaling pathways that might be the cause of those downstream pathways identified by VR. The gene list obtained from Notch pathway-guided COCA includes a notch receptor (NOTCH3) and three ligands (DSL1, JAG1 and JAG2) that can potentially bind to the notch receptor (Figure 5); the list also includes APH-1, a gene encoding a multipass membrane protein, which is required for notch pathway signaling; besides, the list includes many transcription factors as the Notch target genes, revealing a signaling cascade to modulate cell fate by further regulating downstream gene expression. For example, SOX2 in the list is a transcription factor closely related to notch pathway in the development of inner ear [35] and neocortex [36]. While functional enrichment analysis gives us a global picture of that top COCA-ranked genes tend to have better function over-representation than those ranked by VR or EDGE, we also performed Gene Set Enrichment Analysis (GSEA) [37] on the ranked gene lists to further examine whether the ranking can promote the knowledge gene set significantly. In this study we used a web tool, GeneTrail [38], for the GSEA analysis, where false discovery rate (FDR) was used to correct for multiple hypothesis testing (the FDR threshold was set as 10%). We also set the minimum gene number as 10 in order to avoid finding too small sized gene sets. We can see from the results (Table 3 and Table S2(a)-(c)) that COCA ranking tends to boost signaling pathways to be ranked relatively high, while variance-based ranking (VR) mainly boosts ribosome, metabolic pathway and other downstream biological processes (Table 4). None of the signaling pathways from the COCA approach is shown in the GSEA results from the VR approach. We also noticed that the JAK-STAT pathway (GSEA FDR = 0.077) was ranked relatively lower than all the other pathways (GSEA FDR = 0.013, 9.71E-05, 0.042 for Notch, TGF-beta and WNT, respectively). To understand this, we looked further into the GSEA results from the VR approach, and found that JAK-STAT member genes were significantly enriched at the bottom of the VR ranking list (FDR = 0.0279572), suggesting that most of JAK-STAT member genes have lower expression change (thus, relatively weak signal). That could explain, or at least in part, why JAK-STAT pathway was ranked lower than the other pathways (i.e., Notch, TGF-beta and WNT pathways).

**Table 2:** Enriched pathways in the top 500 probe sets ranked by Notch pathway-guided COCA approach

Pathway Term	Count	%	p-value	FDR
Pentose and glucuronate interconversions	8	1.70%	1.64E-06	0.00001847
**Notch signaling pathway**	8	1.70%	5.65E-04	0.00640122
Porphyrin and chlorophyll metabolism	7	1.49%	6.28E-04	0.00719142
p53 signaling pathway	9	1.91%	9.87E-04	0.01107482
Cell cycle	11	2.34%	0.002362	0.02596788
Starch and sucrose metabolism	8	1.70%	0.002805	0.03161464
Androgen and estrogen metabolism	7	1.49%	0.002846	0.03237976
Polyunsaturated fatty acid biosynthesis	4	0.85%	0.015829	0.1738869
Metabolism of xenobiotics by cytochrome P450	7	1.49%	0.022415	0.2321759
Glycolysis/Gluconeogenesis	6	1.28%	0.024584	0.253719
Fructose and mannose metabolism	5	1.06%	0.054919	0.4893802
Galactose metabolism	4	0.85%	0.075736	0.6119058
PPAR signaling pathway	6	1.28%	0.083934	0.6456008

**Table 3:** GSEA analysis results for the gene ranking list generated by Notch pathway-guided COCA approach

Pathway Term	GSEA FDR
**Notch signaling pathway**	0.0133129
DNA replication	0.0775257

**Table 4:** GSEA analysis results for the gene ranking list generated by variance-based ranking (VR)

Pathway Term	GSEA FDR
Ribosome	0.000129461
Parkinson's disease	0.0279572
Metabolic pathways	0.0387599
Oxidative phosphorylation	0.0387599
Homologous recombination	0.0548052
DNA replication	0.0694425
Nucleotide excision repair	0.0694425
Cell cycle	0.0852042

Figure S4 in Additional file 1 shows a Venn diagram of the top 500 genes of those pathways as detected by the COCA ranking approach. As illustrated, most genes are unique to a single pathway and thus pathway-specific, while other genes are common among different pathways, suggestive of possible crosstalk between these pathways. For example, MYO10 and MYL9 are shared between Notch and TGF pathways, while IGF2, APP and S100A6 are common in all the pathways examined. Many of top ranked genes identified by the COCA approach are transcription factors (Table S9 and Table S10). Similarly, some transcription factors are pathway-specific, while others are common among different pathways. For example, the following three transcript factors, JARID2, SOX2 and PITX2, are among the shared transcript factors between Notch and TGFβ pathways, which play a critical role in controlling self-renewal and differentiation of ESCs [32,39]. More interestingly, many oncogenes are among the top ranked genes in each pathways by the COCA approach (Table S9 and Table S10; see Figure 5 for an example), which reaffirms the notion that stem cells are similar to cancer cells on the molecular levels [32].

Specifically we also examined the top 20 genes by looking into their annotations (Table S12(a)-(d)). Within the top 20 genes ranked by Notch pathway-guided COCA (Table S12(a)), there are several genes related to differentiation (Tdgf1, Egr1 and Lefty1), cell growth (Ddit4, Hk2, Phlda2, Egln3 and Igfbp1) and tumor/cancer development (Afp, Sfrp2, Egr1, Hk2 and Phlda2). Some of them are also related to the determination of certain organ as demonstrated by biological studies. For examples, Tdgf1 (Teratocarcinoma-derived growth factor 1, also known as Cripto-1 growth factor) could play a role in the determination of the epiblastic cells that subsequently give rise to the mesoderm [40], and it also contributes to deregulated growth of cancer cells [41]. Note that Tdgf1 was ranked No. 2 by Notch pathway-guided COCA ranking but was ranked No. 906 by variance-based ranking (VR), suggesting that COCA can efficiently boost the ranking of biologically relevant genes. Another gene, left-right determination factor 1 (Lefty1), is known to play a major role during mouse gastrulation and transiently expressed during human embryonic stem cell differentiation [42]. We also note that Lefty1 was ranked No. 9 by COCA ranking but was ranked No. 11,900 by VR, once again suggesting the effectiveness of the COCA approach.

Taking together, the results obtained from the COCA approach provide not only new insights into the complex system of signaling pathways, but also new clues to investigate the molecular mechanisms underlying ESC development. We believe that COCA is of great potential to be utilized in many other studies to help identify biologically meaningful candidate genes and improve our understanding of biological pathways.

## Discussions

Gene ranking is an important task in genomic data analysis to provide biologists with candidate genes of mechanistic interest for further study. However, single gene-based approaches, such as fold-change and SAM [1], suffer from the large noise in microarray data, particularly when the signal-to-noise ratio is relatively low, making gene ranking unreliable. This limitation has motivated many researchers to integrate biological knowledge into data analysis for reliable gene ranking [8,9]. For integration, one must keep in mind that different information sources may not always be sufficiently robust, complete and/or accurate for integration. COCA tries to address this problem by finding a coordinative component from the observation, providing a semi-supervised learning approach for optimization in contrast to combining knowledge and observation heuristically. Such a semi-supervised learning scheme is also a practical solution to the problem, since biological knowledge itself contains false-positives and false-negatives from several sources. For example, knowledge of gene function is often obtained from other biological experiments that contain noise, and the knowledge can be incomplete, too general, and frequently not condition-specific thus irrelevant to the biological conditions under study. Therefore, in the proposed approach, knowledge genes are used to provide guidance only rather than forcing the algorithm to abide by biological knowledge.

Un-supervised methods, not relying on any prior knowledge, could serve as exploratory tools to reveal interesting gene patterns or potential phenotype groupings at an initial data analysis stage. However, for the study with certain biological focus, e.g., looking for the genes related to given biological processes or pathways, semi-supervised or supervised methods are more appropriate to employ than un-supervised methods. If we have sufficient confidence about the knowledge that we have, supervised learning is usually powerful enough to guide us finding important clues. However, since biological knowledge is usually incomplete, supervised methods could be biased and misleading. That is also one of our motivations to perform semi-supervised learning, i.e., using knowledge as the guidance and simultaneously looking at the characteristics of data. Therefore, one should choose un-supervised, semi-supervised or supervised methods in different situations, according to the availability and quality of biological knowledge. It could also be a practical strategy to combine them in order to confirm the findings from different views.

Notice that the optimization criterion defined in Eq. (2) of the COCA approach is similar, at least in principle, to that of a linear discriminate analysis (LDA) approach [43]. In LDA (a supervised learning approach), the criterion is to maximize the ratio of between-class variance to within-class variance; the optimal linear transformation is obtained by maximizing the separability of two classes. The criterion in COCA designed to enable a semi-supervised learning to extract the component of interest guided by prior knowledge genes; the linear transformation is constructed so as to maximize the likelihood of positive knowledge masking with respect to negative knowledge masking.

The importance of biological guidance as incorporated in the COCA approach also needs further discussion. Recently, many statistical decomposition methods have been applied to microarray data in an attempt to elucidate the underlying biological mechanisms [9,13,14,21,44]. However, many of these methods lack an appropriate consideration of biological relevance. Statistical assumptions, such as uncorrelatedness for PCA and independence for ICA, may not be valid in many biological processes, pathways or networks. For example, biological processes or pathways often exhibit redundancy in their signaling and cross talk with other signaling pathways to keep the system robust. Each of these violates the statistical assumptions in PCA and ICA, respectively. Consequently, many statistical decomposition methods are incapable of revealing underlying biological mechanisms. Even if the statistical assumption is considered to be broadly acceptable, improper model selection in any statistical decomposition method will likely bias the results. For example, ICA with an improper model order will either miss important components or generate false components. Cross-validation is often used to select a suitable model order for prediction based on a generalization of model performance. However, it is computationally demanding to evaluate all of the model orders exhaustively; in many cases, even an appropriate model order cannot guarantee the biological relevance of the corresponding results.

COCA has several advantages over conventional statistical decomposition methods such as PCA and ICA. COCA is guided by biological knowledge with the goal of extracting the coordinative component related to a specific biological process or pathway. COCA is also an optimization approach to maximize a coordinative participation ratio of pathway members to non-pathway members. Indeed, the ratio implicitly incorporates a negative reference to the knowledge to make the result biologically comparable. The estimated coordinative component is thus biologically relevant and condition-specific for the study. In addition, COCA avoids the model selection problem by extracting only the desired component rather than performing unnecessary decomposition to uncover all the components underneath. The bootstrapping procedure in COCA further prevents over-fitting of the algorithm when the noise level is relatively high within the data.

Although the exact value of participation matrix (**A**) needs to be estimated according to expression observations in given biological condition, some prior information is available such as predefined memberships of certain pathways. The knowledge can come from different knowledge databases such as KEGG, GO and TRANSPATH, or other knockout (or knockdown) biological experiments. The merit to utilize such prior knowledge is that we can have a clear biological context of the study and a better idea to interpret the results from data analysis. The weakness is that these external knowledge sources may be too generic and not specific enough to describe particular biological situations that we encounter. This, as a matter of fact, is our motivation to propose the COCA approach to utilize prior knowledge but also re-evaluate the knowledge later by participation matrix estimation.

It is worth pointing out that COCA is different from some gene grouping methods that use knowledge to cluster knowledge-related genes together. Here, we would like to highlight some key points that differentiate COCA from gene grouping methods. Firstly, COCA uses knowledge genes to guide the estimation of coordinative direction and such estimation reflects the consistency between the knowledge and the data under certain biological condition. Secondly, while gene grouping methods tend to stick to the originally given knowledge genes, COCA ranks the genes according to the estimated coordinative direction, hence, in a condition-specific manner. Finally, gene grouping methods mainly pay attention to the pattern similarity as calculated directly from gene expression data (**X**), COCA, in contrast, ranks the genes according to their underlying participation matrix (**A**).

Different from traditional gene ranking schemes mainly focusing on the statistical characteristics of data alone, COCA was proposed to rank the genes according to both data and available biological knowledge. However, if relevant biological knowledge is not available, traditional methods still play a major role in prioritizing genes for biological studies. For the study with some confirmed knowledge already known, COCA may serve as a more specific tool for gene ranking, providing an alternative angle to analyze the data.

## Conclusion

In this paper, we have proposed a knowledge-guided method called coordinative component analysis (COCA) for reliable mechanistic gene ranking. The method utilizes partial biological knowledge genes to find coordinative components representing the underlying biological processes or pathways; microarray gene expression data are then projected onto the coordinative components to estimate the participation strengths of genes, these strengths are then used to rank the genes. COCA is mathematically formulated as an optimization problem to maximize the coordinative contribution of member genes to a pathway or network. A bootstrapping procedure has been further developed to overcome the over-fitting problem and provide COCA with a confidence measure for each estimated coordinative component. The proposed COCA approach has been tested with several simulation data and real microarray data, showing an improved performance in gene ranking compared to traditional statistical methods like fold-change, SAM [1] and EDGE [6]. The application of the method to stem cell data has revealed several transcript factors and oncogenes associated with the system development and signaling pathways that are potentially related to cancers. In the future, we will validate the findings through biological experiments to establish their functional role in embryonic development of stem cells. Furthermore, we plan to fully test the proposed method on multiple related data sets to show that COCA can provide us improved ranking results with small variability across the data sets and large relevance to biological pathways.

## Authors' contributions

CW and JX formulated the problem and developed the theoretical framework of the algorithm. CW carried out the development and implementation of the algorithm. HL and MZ directed the application of the algorithm to the stem cell data set. YW, EPH and RC provided technical and biological support to the project. All authors participated in the writing of the manuscript, and have read and approved the manuscript.

## Supplementary Material

Additional file 1 **Supplementary information of the COCA method**. The supplementary information includes a geometrical interpretation of the method, the concept of linear extraction and ambiguity correction, and supplementary results of yeast cell cycle and stem cell studies. Click here for file

Additional file 2 The top 500 probe sets ranked by Notch pathway-guided COCA approach. Click here for file

Additional file 3 The top 500 probe sets ranked by JAK/STAT pathway-guided COCA approach. Click here for file

Additional file 4 The top 500 probe sets ranked by TGFβ pathway-guided COCA approach. Click here for file

Additional file 5 The top 500 probe sets ranked by WNT pathway-guided COCA approach. Click here for file

## References

[B1] Tusher VG Tibshirani R Chu G Significance analysis of microarrays applied to the ionizing radiation response Proc Natl Acad Sci USA 2001 98 9 5116 5121 10.1073/pnas.091062498 11309499 PMC33173

[B2] Mukherjee SN Roberts SJ Sykacek P Gurr SJ Gene ranking using bootstrapped P-values SIGKDD Explor Newsl 2003 5 2 16 22 10.1145/980972.980976

[B3] Shi L Reid LH Jones WD Shippy R Warrington JA Baker SC Collins PJ de Longueville F Kawasaki ES Lee KY The MicroArray Quality Control (MAQC) project shows inter- and intraplatform reproducibility of gene expression measurements Nat Biotechnol 2006 24 9 1151 1161 10.1038/nbt1239 16964229 PMC3272078

[B4] Chen JJ Hsueh HM Delongchamp RR Lin CJ Tsai CA Reproducibility of microarray data: a further analysis of microarray quality control (MAQC) data BMC Bioinformatics 2007 8 412 10.1186/1471-2105-8-412 17961233 PMC2204045

[B5] Opgen-Rhein R Strimmer K Accurate ranking of differentially expressed genes by a distribution-free shrinkage approach Stat Appl Genet Mol Biol 2007 6 Article9 17402924 10.2202/1544-6115.1252

[B6] Storey JD Xiao W Leek JT Tompkins RG Davis RW Significance analysis of time course microarray experiments Proc Natl Acad Sci USA 2005 102 36 12837 12842 10.1073/pnas.0504609102 16141318 PMC1201697

[B7] Furlanello C Serafini M Merler S Jurman G Entropy-based gene ranking without selection bias for the predictive classification of microarray data BMC Bioinformatics 2003 4 54 10.1186/1471-2105-4-54 14604446 PMC293475

[B8] Morrison JL Breitling R Higham DJ Gilbert DR GeneRank: using search engine technology for the analysis of microarray experiments BMC Bioinformatics 2005 6 233 10.1186/1471-2105-6-233 16176585 PMC1261158

[B9] Ma X Lee H Wang L Sun F CGI: a new approach for prioritizing genes by combining gene expression and protein-protein interaction data Bioinformatics 2007 23 2 215 221 10.1093/bioinformatics/btl569 17098772

[B10] Bradley Efron RJT An Introduction to the Bootstrap 1994 New York, Chapman & Hall/CRC

[B11] Jiang W Simon R A comparison of bootstrap methods and an adjusted bootstrap approach for estimating the prediction error in microarray classification Stat Med 2007 26 29 5320 5334 10.1002/sim.2968 17624926

[B12] Kerr MK Linear models for microarray data analysis: hidden similarities and differences J Comput Biol 2003 10 6 891 901 10.1089/106652703322756131 14980016

[B13] Tomfohr J Lu J Kepler TB Pathway level analysis of gene expression using singular value decomposition BMC Bioinformatics 2005 6 225 10.1186/1471-2105-6-225 16156896 PMC1261155

[B14] Devarajan K Nonnegative matrix factorization: an analytical and interpretive tool in computational biology PLoS Comput Biol 2008 4 7 e1000029 10.1371/journal.pcbi.1000029 18654623 PMC2447881

[B15] Pascual-Montano A Carmona-Saez P Chagoyen M Tirado F Carazo JM Pascual-Marqui RD bioNMF: a versatile tool for non-negative matrix factorization in biology BMC Bioinformatics 2006 7 366 10.1186/1471-2105-7-366 16875499 PMC1550731

[B16] Teschendorff AE Journee M Absil PA Sepulchre R Caldas C Elucidating the altered transcriptional programs in breast cancer using independent component analysis PLoS Comput Biol 2007 3 8 e161 10.1371/journal.pcbi.0030161 17708679 PMC1950343

[B17] Liao JC Boscolo R Yang YL Tran LM Sabatti C Roychowdhury VP Network component analysis: reconstruction of regulatory signals in biological systems Proc Natl Acad Sci USA 2003 100 26 15522 15527 10.1073/pnas.2136632100 14673099 PMC307600

[B18] Galbraith SJ Tran LM Liao JC Transcriptome network component analysis with limited microarray data Bioinformatics 2006 22 15 1886 1894 10.1093/bioinformatics/btl279 16766556

[B19] Li H Zhan M Unraveling transcriptional regulatory programs by integrative analysis of microarray and transcription factor binding data Bioinformatics 2008 24 17 1874 1880 10.1093/bioinformatics/btn332 18586698 PMC2519161

[B20] Wang C Xuan J Chen L Zhao P Wang Y Clarke R Hoffman E Motif-directed network component analysis for regulatory network inference BMC Bioinformatics 2008 9 Suppl (S1) S21 10.1186/1471-2105-9-S1-S21 18315853 PMC2259422

[B21] Lee SI Batzoglou S Application of independent component analysis to microarrays Genome Biol 2003 4 11 R76 10.1186/gb-2003-4-11-r76 14611662 PMC329130

[B22] Tanay A Extensive low-affinity transcriptional interactions in the yeast genome Genome Res 2006 16 8 962 972 10.1101/gr.5113606 16809671 PMC1524868

[B23] Bartkute V Sakalauskas L Simultaneous perturbation stochastic approximation of nonsmooth functions European Journal of Operational Research 2007 181 3 1174 1188 10.1016/j.ejor.2005.09.052

[B24] Breiman L Bagging predictors Machine Learning; 1996 1996 123 140

[B25] Dettling M BagBoosting for tumor classification with gene expression data Bioinformatics 2004 20 18 3583 3593 10.1093/bioinformatics/bth447 15466910

[B26] Dudoit S Fridlyand J Bagging to improve the accuracy of a clustering procedure Bioinformatics 2003 19 9 1090 1099 10.1093/bioinformatics/btg038 12801869

[B27] Spellman PT Sherlock G Zhang MQ Iyer VR Anders K Eisen MB Brown PO Botstein D Futcher B Comprehensive identification of cell cycle-regulated genes of the yeast Saccharomyces cerevisiae by microarray hybridization Mol Biol Cell 1998 9 12 3273 3297 9843569 10.1091/mbc.9.12.3273 PMC25624

[B28] Lerou PH Daley GQ Therapeutic potential of embryonic stem cells Blood Rev 2005 19 6 321 331 10.1016/j.blre.2005.01.005 16275420

[B29] Zeng X Rao MS The therapeutic potential of embryonic stem cells: A focus on stem cell stability Curr Opinion Mol Therap 2006 8 4 338 344 16955697

[B30] Sato N Sanjuan IM Heke M Uchida M Naef F Brivanlou AH Molecular signature of human embryonic stem cells and its comparison with the mouse Dev Biol 2003 260 2 404 10.1016/S0012-1606(03)00256-2 12921741

[B31] Miura T Luo Y Khrebtukova I Brandenberger R Zhou D Thies RS Vasicek T Young H Lebkowski J Carpenter MK Monitoring early differentiation events in human embryonic stem cells by massively parallel signature sequencing and expressed sequence tag scan Stem Cells Dev 2004 13 6 694 715 10.1089/scd.2004.13.694 15684837

[B32] Zhan M Genomic studies to explore self-renewal and differentiation properties of embryonic stem cells Front Biosci 2008 13 276 283 10.2741/2678 17981546

[B33] Hailesellasse Sene K Porter CJ Palidwor G Perez-Iratxeta C Muro EM Campbell PA Rudnicki MA Andrade-Navarro MA Gene function in early mouse embryonic stem cell differentiation BMC Genomics 2007 8 85 10.1186/1471-2164-8-85 17394647 PMC1851713

[B34] Huang da W Sherman BT Lempicki RA Systematic and integrative analysis of large gene lists using DAVID bioinformatics resources Nat Protoc 2009 4 1 44 57 10.1038/nprot.2008.211 19131956

[B35] Kiernan AE Xu J Gridley T The Notch ligand JAG1 is required for sensory progenitor development in the mammalian inner ear PLoS Genet 2006 2 1 e4 10.1371/journal.pgen.0020004 16410827 PMC1326221

[B36] Bani-Yaghoub M Tremblay RG Lei JX Zhang D Zurakowski B Sandhu JK Smith B Ribecco-Lutkiewicz M Kennedy J Walker PR Role of Sox2 in the development of the mouse neocortex Dev Biol 2006 295 1 52 66 10.1016/j.ydbio.2006.03.007 16631155

[B37] Subramanian A Tamayo P Mootha VK Mukherjee S Ebert BL Gillette MA Paulovich A Pomeroy SL Golub TR Lander ES Gene set enrichment analysis: a knowledge-based approach for interpreting genome-wide expression profiles Proc Natl Acad Sci USA 2005 102 43 15545 15550 10.1073/pnas.0506580102 16199517 PMC1239896

[B38] Backes C Keller A Kuentzer J Kneissl B Comtesse N Elnakady YA Muller R Meese E Lenhof HP GeneTrail--advanced gene set enrichment analysis Nucleic Acids Res 2007 35 Web Server W186 192 10.1093/nar/gkm323 17526521 PMC1933132

[B39] Sun Y Li H Liu Y Shin S Mattson MP Rao MS Zhan M Cross-species transcriptional profiles establish a functional portrait of embryonic stem cells Genomics 2007 89 1 22 35 10.1016/j.ygeno.2006.09.010 17055697 PMC2658876

[B40] Liguori G Tucci M Montuori N Dono R Lago CT Pacifico F Armenante F Persico MG Characterization of the mouse Tdgf1 gene and Tdgf pseudogenes Mamm Genome 1996 7 5 344 348 10.1007/s003359900100 8661720

[B41] Adamson ED Minchiotti G Salomon DS Cripto: a tumor growth factor and more J Cell Physiol 2002 190 3 267 278 10.1002/jcp.10072 11857442

[B42] Dvash T Sharon N Yanuka O Benvenisty N Molecular analysis of LEFTY-expressing cells in early human embryoid bodies Stem Cells 2007 25 2 465 472 10.1634/stemcells.2006-0179 17038673

[B43] Jieping Y Least squares linear discriminant analysis Proceedings of the 24th international conference on Machine learning 2007 Corvalis, Oregon: ACM

[B44] Gong T Xuan J Wang C Li H Hoffman E Clarke R Wang Y Gene module identification from microarray data using nonnegative independent component analysis Gene Regulation and Systems Biology 2007 1 349 363 PMC2759148 19936101

