# Supplementary material for: Knowledge-guided gene ranking by coordinative component analysis
Source: BMC Bioinformatics. 2010 Mar 30;11:162. doi: 10.1186/1471-2105-11-162 (PMC2865494; doi:10.1186/1471-2105-11-162)
Supplement: Additional file 1 — Supplementary information of the COCA method. The supplementary information includes a geometrical interpretation of the method, the concept of linear extraction and ambiguity correction, and supplementary results of yeast cell cycle and stem cell studies. [file 1471-2105-11-162-S1.PDF]

Supplemental Materials

Knowledge-guided Gene Ranking by Coordinative Component Analysis

Chen Wang[1], Jianhua Xuan[1*], Huai Li[2], Yue Wang [1], Ming Zhan[2], Eric P. Hoffman[3], and Robert Clarke[4]

[1]Department of Electrical and Computer Engineering, Virginia Polytechnic Institute and State University, Arlington, VA, USA

[2]Bioinformatics Unit, RRB, National Institute on Aging, National Institutes of Health, Baltimore, MD, USA

[3]Research Center for Genetic Medicine, Children's National Medical Center, Washington, DC, USA

[4]Lombardi Comprehensive Cancer Center and Department of Oncology, Physiology and Biophysics, Georgetown University, Washington, DC, USA

I. Supplement to Methods

S1. Geometrical understanding of COCA

Treating W_i as a vector in *M*-dimensional space, coordinative direction is the direction on which the knowledge genes can generate larger projection than background genes. Projection along the coordinative direction is the inner product of *j*-th gene vector X_j and W_i, i.e., $a_{ji} = \langle X_j, W_i \rangle = \sum_{k=1}^{M} x_{jk} w_{ik} = X_j^T W_i$. We can further rewrite it in a matrix multiplication form for all the genes: $A_i = \mathbf{X} W_i$

If there are *M* conditions, each gene's expression variation across all the conditions can be regarded as a point in *M*-dimensional space. For the simplicity, we only consider 2-dimensional case to facilitate illustration. As shown in Figure S5(a), each gene's variation pattern across these two conditions will be denoted as one point in 2-D plate, and if two genes have very similar pattern, they will have very similar slope angle in the space. Actually, each gene's variation pattern is caused by different factors (biological processes, pathways etc.), as illustrated in Figure S5(b). Green arrow and red arrow can be regarded

as two biological processes, and ‘empty’ arrow represents the noise effect. So, genes’ patterns are determined by geometry summation of single biological process, multiple biological processes and/or noise effects. The length of arrow is the participation value of a corresponding process; as an example we can see the pattern of genes in the 1st quadratic is strongly influenced by BP2 (Biological Process 2) and weakly influenced by BP1 (Biological Process 1).

Thus, expression will be fully decided by underlying processes, which can be defined as a specific direction in this space (see Figure S5(c)). But without knowing genes’ topology relationship with biological process, we cannot infer the correct direction of the process. Sometimes we only have partial knowledge (with false-positives and false-negatives) about one biological process, and we need to decide which direction is the correct one (see Figure S5(d)).

If we have a wrong guess of the direction of biological process (Figure S5(e)), according to the COCA criterion the averaged participation level of knowledge genes will not significantly higher than background genes, in this case, much smaller. If we have a correct guess of the direction (Figure S5(f)), the averaged participation level of knowledge genes should be much higher than background genes. Once the right direction is estimated according to COCA, we have further opportunity to reduce false-positive genes by filtering out these genes that have small contribution or even do harm to the averaged participation level of knowledge genes, and discover false-negative genes that can contribute to the averaged participation level of knowledge genes greatly.

S2. Linear extraction

This section is aimed to clarify the difference of COCA with common linear decomposition methods, since we only extract one vector each time according to available knowledge. Let us re-write the COCA formulation as follows:

$$\mathbf{X} = \mathbf{AT}. \tag{S1}$$

For ideal case if we know all the topology information, according to the COCA criterion we can estimate the entire matrix A. Rewriting the decomposition into vector form (A in column vector representation and W in row vector representation), one can understand that each extraction can be independent of other extractions. If we assume different biological processes have different activities so that matrix $\mathbf{T}$ is invertible, we can represent the pseudo inverse matrix $\mathbf{W} = \mathbf{T}^{\dagger}$ and multiply it to Eq. (S1) on both sides:

$$\begin{aligned}\mathbf{XW} &= \mathbf{X}\left[W_1, \cdots, W_i, \cdots, W_N\right] = \left[\mathbf{X}W_1, \cdots, \mathbf{X}W_i, \cdots, \mathbf{X}W_N\right] \\ &= \left[A_1, \cdots, A_i, \cdots, A_N\right] = \mathbf{A} = \mathbf{ATW}\end{aligned} \tag{S2}$$

In order to estimate the *i*-th column of $\mathbf{A}$, we can construct a linear filter $\hat{W}$ through COCA algorithm to obtain an estimation $\hat{A}_i$ of the underlying A_i:

$$\mathbf{X}\hat{W}_i = \hat{A}_i. \tag{S3}$$

We can see from Eq. (S2) and Eq. (S3) that when $\hat{W}_i = W_i$ we will have the correct estimation $\hat{A}_i = A_i$.

S3. Ambiguity in COCA estimation

Similar to other latent variable analysis methods (e.g., PCA and ICA), there are also scale and sign ambiguities in the COCA estimation. If we have a $\hat{W}$ that maximizes the defined cost function $J(W)$, any $\hat{W}' = c\hat{W}$ will serve as another feasible solution, where c

can be any non-zero constant. Careful readers may have concerns about the influence of estimation ambiguities on the convergence, ranking results and bootstrapping analysis. Below we give some explanations to address these concerns.

Firstly, estimation ambiguities do not affect the convergence of the COCA algorithm since the scalar difference does not change the value of cost function. As a result the updating algorithm based on stochastic gradient search will not encourage any update in the direction of scalar change. In practice, one can also normalize $\hat{W}$ to a unit norm during each iteration to stabilize the convergence. Secondly, since we use the absolute value of A to rank genes, a sign difference will not affect ranking results. Finally but most importantly, we do need to correct ambiguities to make different estimations based on various bootstrap samples comparable. Let us assume we have a set $\{W^{*i}\}$, each member has different sign and scale ambiguities with respect to underlying true W: $W^{*i} = g^{*i}c^{*i}W$, where $g^{*i} = \pm 1$ indicating sign ambiguity and $c^{*i} > 0$ indicating scale ambiguity. We can perform k-mean clustering to group $\{W^{*i}\}$ into two clusters, and the members within each cluster should have the same sign. Then we perform scale normalization within each cluster, and reverse the sign of one cluster. In this way, we obtain the ambiguity corrected set $\{\hat{W}^{*i}\}$. Each of them has the same range of ambiguities with respect to the true W: $W^{*i} = \hat{g}\hat{c}W$, but within this set the members are all comparable. Although the ambiguities still exist with respect to the true W, it does not affect ranking since we are only interested in each gene's relative contribution.

II. Supplemental Tables (S1-S12)

Table S1. Enriched pathways in the top 500 probe sets ranked by pathway-guided COCA approach: (a) JAK, (b) TGFβ and (c) WNT.

(a)

Pathway Term	Count	%	p-Value	FDR
Cell Communication	17	3.81%	1.81E-05	0.0022622
ECM-receptor interaction	11	2.47%	8.91E-04	0.01106759
Adherens junction	8	1.79%	0.01809	0.2037532
Complement and coagulation cascades	7	1.57%	0.04245	0.4181014
Focal adhesion	13	2.91%	0.04843	0.4618464
TGF-beta signaling pathway	8	1.79%	0.04869	0.4637096
Alzheimer's disease	4	0.90%	0.07488	0.6214758
Prion disease	3	0.67%	0.08037	0.6485869

(b)

Pathway Term	Count	%	p-Value	FDR
Antigen processing and presentation	12	2.82%	1.95E-04	0.00212871
Cell Communication	14	3.29%	3.80E-04	0.00409277
Cell adhesion molecules (CAMs)	12	2.82%	0.009767	0.1042418
ECM-receptor interaction	8	1.88%	0.021768	0.2253211
Lysine degradation	5	1.17%	0.054709	0.4832151
Focal adhesion	12	2.82%	0.056211	0.4887451
TGF-beta signaling pathway	7	1.64%	0.08392	0.6440064
Leukocyte transendothelial migration	8	1.88%	0.090313	0.670197

(c)

Pathway Term	Count	%	p-Value	FDR
ECM-receptor interaction	12	2.52%	1.14E-04	0.00141944
Cell Communication	15	3.15%	1.28E-04	0.00159389
Focal adhesion	14	2.94%	0.013675	0.157906
Small cell lung cancer	8	1.68%	0.02869	0.3046581
Cell adhesion molecules (CAMs)	10	2.10%	0.064062	0.5623693
Prion disease	3	0.63%	0.071239	0.6024614
Complement and coagulation cascades	6	1.26%	0.090903	0.6956516
Leukocyte transendothelial migration	8	1.68%	0.098572	0.7261892

Table S2. GSEA analysis results for the gene ranking lists generated by pathway-guided COCA approach (a) JAK, (b) TGFβ and (c) WNT.

(a)

Pathway Term	GSEA FDR
Cell cycle	0.0473566
Focal adhesion	0.0473566
TGF-beta signaling pathway	0.0473566
Prostate cancer	0.0692307
**Jak-STAT signaling pathway**	0.0774095

(b)

Pathway Term	GSEA FDR
**TGF-beta signaling pathway**	9.71E-05
Focal adhesion	0.0273523
Lysosome	0.0273523
Other glycan degradation	0.0273523
Ubiquitin mediated proteolysis	0.0273523
Long-term potentiation	0.0775771
Nucleotide excision repair	0.0775771
Gap junction	0.0780887
Pentose phosphate pathway	0.0780887
Terpenoid backbone biosynthesis	0.0780887
Biosynthesis of plant hormones	0.0853492
Endocytosis	0.0862163
Tight junction	0.0960988

(c)

Pathway Term	GSEA FDR
Biosynthesis of alkaloids derived from ornithine, lysine and nicotinic acid	0.0418807
Fatty acid metabolism	0.0418807
**Wnt signaling pathway**	0.0418807
Amino sugar and nucleotide sugar metabolism	0.0712975
Biosynthesis of plant hormones	0.0712975
Glutathione metabolism	0.0712975
Keratan sulfate biosynthesis	0.0712975
Prostate cancer	0.0712975
Tryptophan metabolism	0.0712975
p53 signaling pathway	0.0712975
Pathways in cancer	0.075325
Arginine and proline metabolism	0.0870202
Ascorbate and aldarate metabolism	0.0870202
Biosynthesis of alkaloids derived from histidine and purine	0.0870202
Biosynthesis of terpenoids and steroids	0.0870202
Lysine degradation	0.0870202
Biosynthesis of alkaloids derived from shikimate pathway	0.0902419

Table S3. The top 500 probe sets ranked by Notch pathway-guided COCA approach

Please see the pdf file (Table-S3.pdf) [Additional file 2].

Table S4. The top 500 probe sets ranked by JAK/STAT pathway-guided COCA approach

Please see the pdf file (Table-S4.pdf) [Additional file 3].

Table S5. The top 500 probe sets ranked by TGFβ pathway-guided COCA approach

Please see the pdf file (Table-S5.pdf) [Additional file 4].

Table S6. The top 500 probe sets ranked by WNT pathway-guided COCA approach

Please see the pdf file (Table-S6.pdf) [Additional file 5].

Table S7. Enriched pathways in the top 500 probe sets ranked by the variance-based ranking (VR) method

Pathway Term	Count	%	p-value	FDR
Ribosome	25	4.84%	1.11E-14	0.0
Oxidative phosphorylation	14	2.71%	0.001137	0.0141
Carbon fixation	5	0.97%	0.010325	0.1215
Ubiquitin mediated proteolysis	11	2.13%	0.027168	0.2909
Glycolysis / Gluconeogenesis	6	1.16%	0.047022	0.4518
Adherens junction	7	1.35%	0.062235	0.5515
Huntington's disease	4	0.77%	0.08213	0.6569
Regulation of actin cytoskeleton	13	2.51%	0.096168	0.7169

Table S8. Enriched pathways in the top 500 probe sets ranked by the EDGE-based ranking method

Pathway Term	Count	%	p-value	FDR
Cell Communication	15	3.54%	6.15E-05	0.007678
Prion disease	4	0.94%	0.007311	0.087526
Tight junction	9	2.12%	0.058215	0.527002
Small cell lung cancer	9	2.12%	0.006328	0.076178
Cell adhesion molecules (CAMs)	9	2.12%	0.09856	0.726143
ECM-receptor interaction	9	2.12%	0.005116	0.062016

Table S9. Number of transcription factors and oncogenes in the top 500 probe sets ranked by the proposed COCA approach, variance-based ranking (VR) and EDGE-based ranking

Method	No. of transcription factors	No. of oncogenes
Notch pathway-guided COCA	46	9
JAK pathway-guided COCA	44	5
TGF pathway-guided COCA	43	3
WNT pathway-guided COCA	34	7
Variance-based ranking (VR)	29	0
EDGE-based ranking	19	4

Table S10. The transcription factors identified by Notch pathway-guided COCA

GENE_SYMBOL	Gene Name
MYCN	V-MYC MYELOCYTOMATOSIS VIRAL RELATED ONCOGENE, NEUROBLASTOMA DERIVED (AVIAN)
TAF4	TAF4 RNA POLYMERASE II, TATA BOX BINDING PROTEIN (TBP)-ASSOCIATED FACTOR, 135KDA
NFKBIA	NUCLEAR FACTOR OF KAPPA LIGHT POLYPEPTIDE GENE ENHANCER IN B-CELLS INHIBITOR, ALPHA
ZFP36L2	ZINC FINGER PROTEIN 36, C3H TYPE-LIKE 2
KEAP1	KELCH-LIKE ECH-ASSOCIATED PROTEIN 1
GLI2	GLI-KRUPPEL FAMILY MEMBER GLI2
PSIP1	PC4 AND SFRS1 INTERACTING PROTEIN 1
PHF21A	PHD FINGER PROTEIN 21A
AFF4	AF4/FMR2 FAMILY, MEMBER 4
NCOR2	NUCLEAR RECEPTOR CO-REPRESSOR 2
TCF4	TRANSCRIPTION FACTOR 4
HNF4A	HEPATOCYTE NUCLEAR FACTOR 4, ALPHA
CBX5	CHROMOBOX HOMOLOG 5 (HP1 ALPHA HOMOLOG, DROSOPHILA)
BACH1	BTB AND CNC HOMOLOGY 1, BASIC LEUCINE ZIPPER TRANSCRIPTION FACTOR 1
PITX2	PAIRED-LIKE HOMEODOMAIN TRANSCRIPTION FACTOR 2
GCN5L2	GCN5 GENERAL CONTROL OF AMINO-ACID SYNTHESIS 5-LIKE 2 (YEAST)
EGR1	EARLY GROWTH RESPONSE 1
DR1	DOWN-REGULATOR OF TRANSCRIPTION 1, TBP-BINDING (NEGATIVE COFACTOR 2)
SNAPC2	SMALL NUCLEAR RNA ACTIVATING COMPLEX, POLYPEPTIDE 2, 45KDA
TRIM25	TRIPARTITE MOTIF-CONTAINING 25
CREB3	CAMP RESPONSIVE ELEMENT BINDING PROTEIN 3
TEAD2	TEA DOMAIN FAMILY MEMBER 2
MEF2D	MADS BOX TRANSCRIPTION ENHANCER FACTOR 2, POLYPEPTIDE D (MYOCYTE ENHANCER FACTOR 2D)
ETV5	ETS VARIANT GENE 5 (ETS-RELATED MOLECULE)
DEK	DEK ONCOGENE (DNA BINDING)
ESRRB	ESTROGEN-RELATED RECEPTOR BETA
HDAC2	HISTONE DEACETYLASE 2
IVNS1ABP	INFLUENZA VIRUS NS1A BINDING PROTEIN
TRIM28	TRIPARTITE MOTIF-CONTAINING 28
RLF	REARRANGED L-MYC FUSION
TSC22D1	TSC22 DOMAIN FAMILY, MEMBER 1
NFATC4	NUCLEAR FACTOR OF ACTIVATED T-CELLS, CYTOPLASMIC, CALCINEURIN-DEPENDENT 4
PML	PROMYELOCYTIC LEUKEMIA
JARID2	JUMONJI, AT RICH INTERACTIVE DOMAIN 2
AMOT	ANGIOMOTIN

JARID1B	JUMONJI, AT RICH INTERACTIVE DOMAIN 1B (RBP2-LIKE)
MYST4	MYST HISTONE ACETYLTRANSFERASE (MONOCYTIC LEUKEMIA) 4
TBP	TATA BOX BINDING PROTEIN
SF1	SPLICING FACTOR 1
SOX2	SRY (SEX DETERMINING REGION Y)-BOX 2
BTBD14B	BTB (POZ) DOMAIN CONTAINING 14B
KLF2	KRUPPEL-LIKE FACTOR 2 (LUNG)
ZFP106	ZINC FINGER PROTEIN 106 HOMOLOG (MOUSE)
PLAU	PLASMINOGEN ACTIVATOR, UROKINASE
MYB	V-MYB MYELOBLASTOSIS VIRAL ONCOGENE HOMOLOG (AVIAN)
FALZ	Fetal Alz-50 clone 1 protein, bromodomain PHD finger transcription factor

Table S11. The oncogenes identified by Notch pathway-guided COCA

GENE_SYMBOL	Gene Name
NOTCH3	notch gene homolog 3 (drosophila)
MYCN	v-myc myelocytomatosis viral related oncogene, neuroblastoma derived (avian)
CCND1	cyclin d1
FGFR1	fibroblast growth factor receptor 1
CCND2	cyclin d2
DEK	riken cdna 1810019e15 gene
ETV5	riken cdna 8430401f14 gene
MYB	myeloblastosis oncogene
PML	promyelocytic leukemia

Table S12. Annotations of top-20 genes ranked by pathway guided COCA:

(a) NOTCH, (b) JAK, (c) TGFβ and (d) WNT.

(a)

Rank	GeneSymbol	Gene Description
1	Afp	Alpha-fetoprotein. This gene encodes alpha-fetoprotein, a major plasma protein produced by the yolk sac and the liver during **fetal life**. Alpha-fetoprotein expression in adults is often associated with hepatoma or teratoma.
2	Tdgf1	Epidermal growth factor-like cripto protein CR1. Could play a role in the **determination** of the epiblastic cells that subsequently give rise to the mesoderm.
3	Rbp4	Plasma retinol-binding protein. This protein belongs to the lipocalin family and is the specific carrier for retinol (vitamin A alcohol) in the blood. It delivers retinol from the liver stores to the peripheral tissues. In plasma, the RBP-retinol complex interacts with transthyretin which prevents its loss by filtration through the kidney glomeruli. A deficiency of vitamin A blocks secretion of the binding

		protein posttranslationally and results in defective delivery and supply to the epidermal cells.
4	Sfrp2	Secreted apoptosis-related protein 1. This gene encodes a member of the SFRP family that contains a cysteine-rich domain homologous to the putative Wnt-binding site of Frizzled proteins. SFRPs act as soluble **modulators** of Wnt **signaling.** Methylation of this gene is a potential marker for the presence of colorectal cancer.
5	Trh	Thyrotropin-releasing hormone. Functions as a **regulator** of the biosynthesis of TSH in the anterior pituitary gland and as a neurotransmitter/ neuromodulator in the central and peripheral nervous systems.
6	Tagln	Smooth muscle protein 22-alpha. The protein encoded by this gene is a transformation and shape-change sensitive actin cross-linking/gelling protein found in fibroblasts and smooth muscle. Its expression is down-regulated in many cell lines, and this down-regulation may be an early and sensitive marker for the onset of transformation. A functional role of this protein is unclear. Two transcript variants encoding the same protein have been found for this gene.
7	Mt2	Metallothionein 2A. Metallothioneins have a high content of cysteine residues that bind various heavy metals; these proteins are transcriptionally regulated by both heavy metals and glucocorticoids.
8	Egr1	Early growth response protein 1. The protein encoded by this gene belongs to the EGR family of C2H2-type zinc-finger proteins. It is a nuclear protein and functions as a transcriptional regulator. The products of target genes it activates are required for **differentitation** and **mitogenesis.** Studies suggest this is a cancer suppressor gene.
9	Lefty1	Left-right determination factor B. This gene encodes a member of the TGF-beta family of proteins. A similar secreted protein in mouse plays a role in left-right asymmetry **determination of organ systems** during development. Alternative processing of this protein can yield three different products. This gene is closely linked to both a related family member and a related pseudogene.
10	Hspb2	Heat-shock protein beta-2.
11	Ddit4	DNA-damage-inducible transcript 4.**Inhibits cell growth** by regulating the FRAP1 pathway upstream of the TSC1-TSC2 complex and downstream of AKT1. Promotes neuronal cell death
12	Scd2	Acyl-CoA-desaturase 4. Stearoyl-CoA desaturase (SCD; EC 1.14.99.5) is an integral membrane protein of the endoplasmic reticulum that catalyzes the formation of monounsaturated fatty acids from saturated fatty acids. SCD may be a key regulator of energy metabolism with a role in obesity and dislipidemia. Four SCD isoforms, Scd1 through Scd4, have been identified in mouse. In contrast, only 2 SCD isoforms, SCD1 (MIM 604031) and SCD5, have been identified in human.
13	Hk2	Hexokinase type II. Hexokinases phosphorylate glucose to produce glucose-6-phosphate, the first step in most glucose metabolism pathways. This gene encodes hexokinase 2, the predominant form found in skeletal muscle. It localizes to the outer membrane of mitochondria. Expression of this gene is insulin-responsive, and studies in rat suggest that it is involved in the increased rate of glycolysis seen in rapidly growing cancer cells.

14	Phlda2	Tumor-suppressing STF cDNA 3 protein. This gene is one of several genes in the imprinted gene domain of 11p15.5 which is considered to be an important **tumor suppressor** gene region. Alterations in this region may be associated with the Beckwith-Wiedemann syndrome, Wilms tumor, rhabdomyosarcoma, adrenocortical carcinoma, and lung, ovarian, and breast cancer. Studies of the mouse gene, however, which is also located in an imprinted gene domain, have shown that the product of this gene regulates placental growth.
15	Egln3	Hypoxia-inducible factor prolyl hydroxylase 3. Catalyzes the post-translational formation of 4-hydroxyproline in hypoxia-inducible factor (HIF) alpha proteins. Hydroxylates HIF-1 alpha at 'Pro-564', and HIF-2 alpha. Functions as a cellular oxygen sensor and, under normoxic conditions, targets HIF through the hydroxylation for proteasomal degradation via the von Hippel-Lindau ubiquitination complex. May play a role in **cell growth regulation** in muscle cells and in **apoptosis** in neuronal tissue. Promotes cell death through a caspase-dependent mechanism.
16	Lrpprc	Leucine-rich PPR motif-containing protein. This gene encodes a protein that is leucine-rich and is thought to play a role in regulating the interaction of the cytoskeleton with a variety of cellular processes. Mutations in this gene are associated with the French-Canadian type of Leigh syndrome. Transcripts ranging in size from 4.8 to 7.0 kb which result from alternative polyadenylation have been reported for this gene.
17	Hnrnpm	Heterogenous nuclear ribonucleoprotein M. This gene belongs to the subfamily of ubiquitously expressed heterogeneous nuclear ribonucleoproteins (hnRNPs). The hnRNPs are RNA binding proteins and they complex with heterogeneous nuclear RNA (hnRNA). These proteins are associated with pre-mRNAs in the nucleus and appear to influence pre-mRNA processing and other aspects of mRNA metabolism and transport. While all of the hnRNPs are present in the nucleus, some seem to shuttle between the nucleus and the cytoplasm. The hnRNP proteins have distinct nucleic acid binding properties. The protein encoded by this gene has three repeats of quasi-RRM domains that bind to RNAs. This protein also constitutes a monomer of the N-acetylglucosamine-specific receptor which is postulated to trigger selective recycling of immature GlcNAc-bearing thyroglobulin molecules. Multiple alternatively spliced transcript variants are known for this gene but only two transcripts has been isolated.
18	Apoc2	Apolipoprotein C-II. The protein encoded by this gene is secreted in plasma where it is a component of very low density lipoprotein. This protein activates the enzyme lipoprotein lipase, which hydrolyzes triglycerides and thus provides free fatty acids for cells. Mutations in this gene cause hyperlipoproteinemia type IB, characterized by hypertriglyceridemia, xanthomas, and increased risk of pancreatitis and early atherosclerosis.
19	Ell2	ELL-related RNA polymerase II, elongation factor. Elongation factor that can increase the catalytic rate of RNA polymerase II transcription by suppressing transient pausing by the polymerase at multiple sites along the DNA.
20	Igfbp1	Insulin-like growth factor binding protein 1. IGF-binding proteins prolong the half-life of the IGFs and have been shown to either **inhibit or stimulate the growth promoting effects** of the IGFs on cell culture. They alter the interaction of IGFs with their cell surface receptors. Promotes cell migration.

(b)

Rank	GeneSymbol	Gene Description
1	Ctsj	Cathepsin J precursor.
2	Prl2c2	Prolactin family 2. May have a role in embryonic development. It is likely to

		provide a growth stimulus to target cells in maternal and fetal tissues during the development of the embryo at mid-gestation.
3	Lgals3	Galactose-specific lectin 3. Galactose-specific lectin which binds IgE. May mediate with the alpha-3, beta-1 integrin the stimulation by CSPG4 of endothelial cells migration. Together with DMBT1, required for terminal differentiation of columnar epithelial cells during **early embryogenesis**.
4	Gkn2	Gastrokine 2. Down-regulated in gastric cancer GDDR.
5	Prl2a1	Prolactin-2A1 precursor (Placental prolactin-like protein M). Invading mouse trophoblast cells possess endocrine activities, including the expression of PLP-M.
6	Rhox6	Reproductive homeobox 6. 18 days pregnant adult female placenta and extra embryonic tissue cDNA
7	Prl3b1	Chorionic somatomammotropin hormone 2. Expression of mPL-II and Igf2 is highly related to placental development in mice.
8	Prl7d1	Prolactin-7D1 precursor (Proliferin-related protein). Adult female placenta cDNA.
9	Vim	Vimentin. Along with the microfilaments (actins) and microtubules (tubulins), the intermediate filaments represent a third class of well-characterized cytoskeletal elements. The subunits display a tissue-specific pattern of expression. Desmin (MIM 125660) is the subunit specific for muscle and vimentin the subunit specific for mesenchymal tissue.
10	Afp	Alpha-fetoprotein. This gene encodes alpha-fetoprotein, a major plasma protein produced by the yolk sac and the liver during fetal life. Alpha-fetoprotein expression in adults is often associated with hepatoma or teratoma.
11	Cdkn1c	Cyclin-dependent kinase inhibitor 1C. The protein encoded by this gene is a tight-binding, strong inhibitor of several G1 cyclin/Cdk complexes and a negative regulator of cell **proliferation**. Mutations in this gene are implicated in sporadic cancers and Beckwith-Wiedemann syndorome, suggesting that this gene is a tumor suppressor candidate. Three transcript variants encoding two different isoforms have been found for this gene.
12	Col4a2	Collagen, type IV, alpha 2. This gene encodes one of the six subunits of type IV collagen, the major structural component of basement membranes. The C-terminal portion of the protein, known as canstatin, is an inhibitor of **angiogenesis and tumor growth**. Like the other members of the type IV collagen gene family, this gene is organized in a head-to-head conformation with another type IV collagen gene so that each gene pair shares a common promoter.
13	Col4a1	Collagen, type IV, alpha 1. This gene encodes the major type IV alpha collagen chain of basement membranes. Like the other members of the type IV collagen gene family, this gene is organized in a head-to-head conformation with another type IV collagen gene so that each gene pair shares a common promoter.
14	Krt19	Keratin. The protein encoded by this gene is a member of the keratin family. The keratins are intermediate filament proteins responsible for the structural integrity of epithelial cells and are subdivided into cytokeratins and hair keratins. The type I cytokeratins consist of acidic proteins which are arranged in pairs of heterotypic keratin chains. Unlike its related family members, this smallest known acidic cytokeratin is not paired with a basic cytokeratin in epithelial cells. It is specifically expressed in the periderm, the transiently superficial layer that envelopes the developing epidermis. The type I cytokeratins are clustered in a region of chromosome 17q12-q21.

15	Lama1	Laminin, alpha 1. Binding to cells via a high affinity receptor, laminin is thought to mediate the attachment, migration and organization of cells into tissues during **embryonic development** by interacting with other extracellular matrix components.
16	Rbp4	Retinol binding protein 4, plasma. This protein belongs to the lipocalin family and is the specific carrier for retinol (vitamin A alcohol) in the blood. It delivers retinol from the liver stores to the peripheral tissues. In plasma, the RBP-retinol complex interacts with transthyretin which prevents its loss by filtration through the kidney glomeruli. A deficiency of vitamin A blocks secretion of the binding protein posttranslationally and results in defective delivery and supply to the epidermal cells.
17	Cited2	Cbp/p300-interacting transactivator, with Glu/Asp-rich. Interferes with the binding of transcription factors HIF-1a and STAT2 to p300/CBP
18	S100a6	S100 calcium binding protein A6 (calcyclin). The protein encoded by this gene is a member of the S100 family of proteins containing 2 EF-hand calcium-binding motifs. S100 proteins are localized in the cytoplasm and/or nucleus of a wide range of cells, and involved in the regulation of a number of cellular processes such as **cell cycle progression and differentiation.** S100 genes include at least 13 members which are located as a cluster on chromosome 1q21. This protein may function in stimulation of Ca2+-dependent insulin release, stimulation of prolactin secretion, and exocytosis. Chromosomal rearrangements and altered expression of this gene have been implicated in melanoma.
19	Ldhb	L-lactate dehydrogenase B.
20	Sycp3	Synaptonemal complex protein 3. Component of the transverse filaments of synaptonemal complexes (SCS), formed between homologous chromosomes during meiotic prophase. Has an essential meiotic function in spermatogenesis. May be important for testis **development**.

(c)

Rank	GeneSymbol	Gene Description
1	Afp	Alpha-fetoprotein. This gene encodes alpha-fetoprotein, a major plasma protein produced by the yolk sac and the liver during **fetal life**. Alpha-fetoprotein expression in adults is often associated with hepatoma or teratoma.
2	Rbp4	Retinol binding protein 4, plasma. This protein belongs to the lipocalin family and is the specific carrier for retinol (vitamin A alcohol) in the blood. It delivers retinol from the liver stores to the peripheral tissues. In plasma, the RBP-retinol complex interacts with transthyretin which prevents its loss by filtration through the kidney glomeruli. A deficiency of vitamin A blocks secretion of the binding protein posttranslationally and results in defective delivery and supply to the epidermal cells.

3	Ttr	Thyroxine-binding prealbumin. This gene encodes transthyretin, one of the three prealbumins including alpha-1-antitrypsin, transthyretin and orosomucoid. Transthyretin is a carrier protein; it **transports thyroid hormones** in the plasma and cerebrospinal fluid, and also transports retinol (vitamin A) in the plasma. The protein consists of a tetramer of identical subunits. More than 80 different mutations in this gene have been reported; most mutations are related to amyloid deposition, affecting predominantly peripheral nerve and/or the heart, and a small portion of the gene mutations is non-amyloidogenic. The diseases caused by mutations include amyloidotic polyneuropathy, euthyroid hyperthyroxinaemia, amyloidotic vitreous pacities, cardiomyopathy, oculoleptomeningeal amyloidosis, meningocerebrovascular amyloidosis, carpal tunnel syndrome, etc.
4	Krt8	Keratin 8. This gene is a member of the type II keratin family clustered on the long arm of chromosome 12. Type I and type II keratins heteropolymerize to form intermediate-sized filaments in the cytoplasm of epithelial cells. The product of this gene typically dimerizes with keratin 18 to form an intermediate filament in simple single-layered epithelial cells. This protein plays a role in maintaining cellular structural integrity and also functions in **signal transduction** and cellular **differentiation**. Mutations in this gene cause cryptogenic cirrhosis.
5	Apoc2	Apolipoprotein C-II. The protein encoded by this gene is secreted in plasma where it is a component of very low density lipoprotein. This protein activates the enzyme lipoprotein lipase, which hydrolyzes triglycerides and thus provides free fatty acids for cells. Mutations in this gene cause hyperlipoproteinemia type IB, characterized by hypertriglyceridemia, xanthomas, and increased risk of pancreatitis and early atherosclerosis.
6	S100g	S100 calcium binding protein G. This gene encodes calbindin D9K, a vitamin D-dependent calcium-binding protein. This cytosolic protein belongs to a family of calcium-binding proteins that includes calmodulin, parvalbumin, troponin C, and S100 protein. In the intestine, the protein is vitamin D-dependent and its expression correlates with calcium transport activity. The protein may increase Ca2+ absorption by buffering Ca2+ in the cytoplasm and increase ATP-dependent Ca2+ transport in duodenal basolateral membrane vesicles.
7	Ctgf	Insulin-like growth factor-binding protein 8. Major connective tissue mitoattractant secreted by vascular endothelial cells. **Promotes proliferation and differentiation** of chondrocytes. Mediates heparin- and divalent cation-dependent cell adhesion in many cell types including fibroblasts, myofibroblasts, endothelial and epithelial cells. Enhances fibroblast growth factor-induced DNA synthesis
8	Spink3	Pancreatic secretory trypsin inhibitor. The protein encoded by this gene is a trypsin inhibitor, which is secreted from pancreatic acinar cells into pancreatic juice. It is thought to function in the prevention of trypsin-catalyzed premature activation of zymogens within the pancreas and the pancreatic duct. Mutations in this gene are associated with hereditary pancreatitis and tropical calcific pancreatitis.
9	Apoa1	Apolipoprotein A-I. This gene encodes apolipoprotein A-I, which is the major protein component of high density lipoprotein (HDL) in plasma. The protein promotes cholesterol efflux from tissues to the liver for excretion, and it is a cofactor for lecithin cholesterolacyltransferase (LCAT) which is responsible for the formation of most plasma cholesteryl esters. This gene is closely linked with two other apolipoprotein genes on chromosome 11. Defects in this gene are associated with HDL deficiencies, including Tangier disease, and with systemic non-neuropathic amyloidosis.

10	Krt18	Cytokeratin 18, Cell **proliferation**-inducing gene 46 protein. KRT18 encodes the type I intermediate filament chain keratin 18. Keratin 18, together with its filament partner keratin 8, are perhaps the most commonly found members of the intermediate filament gene family. They are expressed in single layer epithelial tissues of the body. Mutations in this gene have been linked to cryptogenic cirrhosis. Two transcript variants encoding the same protein have been found for this gene.
11	H19	H19, imprinted maternally expressed transcript. This gene expresses a non-coding RNA, and functions as a **tumor suppressor**. The gene is located in an imprinted region of chromosome 11 near the insulin-like growth factor 2 (IGF2) gene. Expression of this gene and IGF2 are imprinted so that this gene is only expressed from the maternally-inherited chromosome, and IGF2 is only expressed from the paternally-inherited chromosome. A region of paternal-specific methylation upstream of this gene is required for the imprinting of these genes. Mutations in this gene are associated with Beckwith-Wiedemann Syndrome and Wilms tumorigenesis.
12	Car4	Carbonate dehydratase IV. Carbonic anhydrases (CAs) are a large family of zinc metalloenzymes that catalyze the reversible hydration of carbon dioxide. They participate in a variety of biological processes, including respiration, calcification, acid-base balance, bone resorption, and the formation of aqueous humor, rebrospinal fluid, saliva, and gastric acid. They show extensive diversity in tissue distribution and in their subcellular localization. This gene encodes a glycosylphosphatidyl-inositol-anchored membrane isozyme expressed on the luminal surfaces of pulmonary (and certain other) capillaries and proximal renal tubules. Its exact function is not known; however, it may have a role in inherited renal abnormalities of bicarbonate transport.
13	Tpm1	Alpha tropomyosin. This gene is a member of the tropomyosin family of highly conserved, widely distributed actin-binding proteins involved in the contractile system of striated and smooth muscles and the cytoskeleton of non-muscle cells. Tropomyosin is composed of two alpha-helical chains arranged as a coiled-coil. It is polymerized end to end along the two grooves of actin filaments and provides stability to the filaments. The encoded protein is one type of alpha helical chain that forms the predominant tropomyosin of striated muscle, where it also functions in association with the troponin complex to regulate the calcium-dependent interaction of actin and myosin during muscle contraction. In smooth muscle and non-muscle cells, alternatively spliced transcript variants encoding a range of isoforms have been described. Mutations in this gene are associated with type 3 familial hypertrophic cardiomyopathy.
14	Gpx3	Glutathione peroxidase 3. This gene product belongs to the glutathione peroxidase family, which functions in the detoxification of hydrogen peroxide. It contains a selenocysteine (Sec) residue at its active site. The selenocysteine is encoded by the UGA codon, which normally signals translation termination. The 3' UTR of Sec-containing genes have a common stem-loop structure, the sec insertion sequence (SECIS), which is necessary for the recognition of UGA as a Sec codon rather than as a stop signal.
15	Thbs1	Thrombospondin 1. The protein encoded by this gene is a subunit of a disulfide-linked homotrimeric protein. This protein is an adhesive glycoprotein that mediates cell-to-cell and cell-to-matrix interactions. This protein can bind to fibrinogen, fibronectin, laminin, type V collagen and integrins alpha-V/beta-1. This protein has been shown to play roles in platelet **aggregation, angiogenesis, and tumorigenesis.**

16	Tagln	Smooth muscle protein 22-alpha. The protein encoded by this gene is a transformation and shape-change sensitive actin cross-linking/gelling protein found in fibroblasts and smooth muscle. Its expression is down-regulated in many cell lines, and this down-regulation may be an early and sensitive marker for the onset of transformation. A functional role of this protein is unclear. Two transcript variants encoding the same protein have been found for this gene.
17	Mt2	metallothionein 2A. Metallothioneins have a high content of cysteine residues that bind various heavy metals; these proteins are transcriptionally regulated by both heavy metals and glucocorticoids.
18	Prl2c2	Prolactin family 2. May have a role in embryonic development. It is likely to provide a growth stimulus to target cells in maternal and fetal tissues during the development of the embryo at mid-gestation.
19	Gbp1	Guanine nucleotide-binding protein 1. Guanylate binding protein expression is induced by interferon. Guanylate binding proteins are characterized by their ability to specifically bind guanine nucleotides (GMP, GDP, and GTP) and are distinguished from the GTP-binding proteins by the presence of 2 binding motifs rather than 3.
20	Id1	DNA-binding protein inhibitor ID-1. The protein encoded by this gene is a helix-loop-helix (HLH) protein that can form heterodimers with members of the basic HLH family of transcription factors. The encoded protein has no DNA binding activity and therefore can inhibit the DNA binding and transcriptional activation ability of basic HLH proteins with which it interacts. This protein may play a role in **cell growth, senescence, and differentiation**. Two transcript variants encoding different isoforms have been found for this gene.

(d)

Rank	GeneSymbol	Gene Description
1	Ctsj	Cathepsin J precursor.
2	Prl2c2	Prolactin family 2. May have a role in embryonic development. It is likely to provide a growth stimulus to target cells in maternal and fetal tissues during the development of the embryo at mid-gestation.
3	Lgals3	Galactose-specific lectin 3. Galactose-specific lectin which binds IgE. May mediate with the alpha-3, beta-1 integrin the stimulation by CSPG4 of endothelial cells migration. Together with DMBT1, required for terminal differentiation of columnar epithelial cells during **early embryogenesis**.
4	Gkn2	Gastrokine 2. Down-regulated in gastric cancer GDDR.
5	Prl2a1	Prolactin-2A1 precursor (Placental prolactin-like protein M). Invading mouse trophoblast cells possess endocrine activities, including the expression of PLP-M.
6	Rhox6	Reproductive homeobox 6. 18 days pregnant adult female placenta and extra embryonic tissue cDNA
7	Prl3b1	Chorionic somatomammotropin hormone 2. Expression of mPL-II and Igf2 is highly related to placental development in mice.
8	Prl7d1	Prolactin-7D1 precursor (Proliferin-related protein). Adult female placenta cDNA.
9	Vim	Vimentin. Along with the microfilaments (actins) and microtubules (tubulins), the intermediate filaments represent a third class of well-characterized cytoskeletal elements. The subunits display a tissue-specific pattern of expression. Desmin (MIM 125660) is the subunit specific for muscle and vimentin the subunit specific for mesenchymal tissue.

10	Afp	Alpha-fetoprotein. This gene encodes alpha-fetoprotein, a major plasma protein produced by the yolk sac and the liver during fetal life. Alpha-fetoprotein expression in adults is often associated with hepatoma or teratoma.
11	Cdkn1c	Cyclin-dependent kinase inhibitor 1C. The protein encoded by this gene is a tight-binding, strong inhibitor of several G1 cyclin/Cdk complexes and a negative regulator of cell **proliferation**. Mutations in this gene are implicated in sporadic cancers and Beckwith-Wiedemann syndorome, suggesting that this gene is a tumor suppressor candidate. Three transcript variants encoding two different isoforms have been found for this gene.
12	Col4a2	Collagen, type IV, alpha 2. This gene encodes one of the six subunits of type IV collagen, the major structural component of basement membranes. The C-terminal portion of the protein, known as canstatin, is an inhibitor of **angiogenesis and tumor growth**. Like the other members of the type IV collagen gene family, this gene is organized in a head-to-head conformation with another type IV collagen gene so that each gene pair shares a common promoter.
13	Col4a1	Collagen, type IV, alpha 1. This gene encodes the major type IV alpha collagen chain of basement membranes. Like the other members of the type IV collagen gene family, this gene is organized in a head-to-head conformation with another type IV collagen gene so that each gene pair shares a common promoter.
14	Krt19	Keratin. The protein encoded by this gene is a member of the keratin family. The keratins are intermediate filament proteins responsible for the structural integrity of epithelial cells and are subdivided into cytokeratins and hair keratins. The type I cytokeratins consist of acidic proteins which are arranged in pairs of heterotypic keratin chains. Unlike its related family members, this smallest known acidic cytokeratin is not paired with a basic cytokeratin in epithelial cells. It is specifically expressed in the periderm, the transiently superficial layer that envelopes the developing epidermis. The type I cytokeratins are clustered in a region of chromosome 17q12-q21.
15	Lama1	Laminin, alpha 1. Binding to cells via a high affinity receptor, laminin is thought to mediate the attachment, migration and organization of cells into tissues during **embryonic development** by interacting with other extracellular matrix components.
16	Rbp4	Retinol binding protein 4, plasma. This protein belongs to the lipocalin family and is the specific carrier for retinol (vitamin A alcohol) in the blood. It delivers retinol from the liver stores to the peripheral tissues. In plasma, the RBP-retinol complex interacts with transthyretin which prevents its loss by filtration through the kidney glomeruli. A deficiency of vitamin A blocks secretion of the binding protein posttranslationally and results in defective delivery and supply to the epidermal cells.
17	Cited2	Cbp/p300-interacting transactivator, with Glu/Asp-rich. Interferes with the binding of transcription factors HIF-1a and STAT2 to p300/CBP
18	S100a6	S100 calcium binding protein A6 (calcyclin). The protein encoded by this gene is a member of the S100 family of proteins containing 2 EF-hand calcium-binding motifs. S100 proteins are localized in the cytoplasm and/or nucleus of a wide range of cells, and involved in the regulation of a number of cellular processes such as **cell cycle progression and differentiation.** S100 genes include at least 13 members which are located as a cluster on chromosome 1q21. This protein may function in stimulation of Ca2+-dependent insulin release, stimulation of prolactin secretion, and exocytosis. Chromosomal rearrangements and altered expression of this gene have been implicated in melanoma.
19	Ldhb	L-lactate dehydrogenase B.

20	Sycp3	Synaptonemal complex protein 3. Component of the transverse filaments of synaptonemal complexes (SCS), formed between homologous chromosomes during meiotic prophase. Has an essential meiotic function in spermatogenesis. May be important for testis **development**.

III. Supplemental Figures (S1-S5)

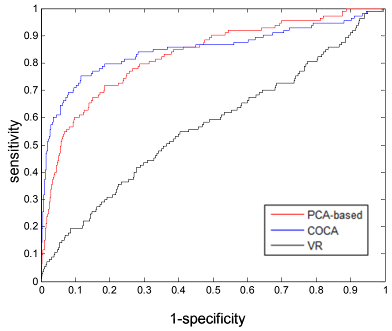

(a)

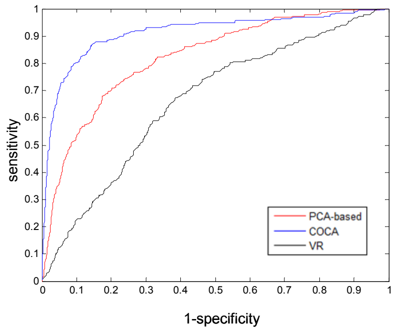

(b)

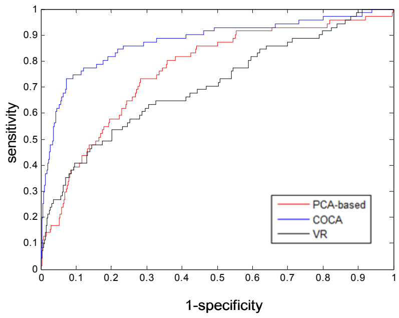

(c)

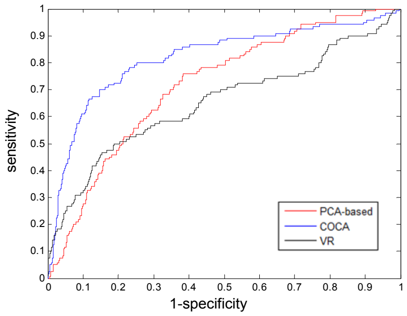

(d)

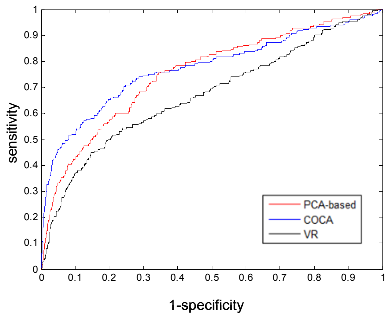

(e)

Figure S1. Receiver Operator Characteristic (ROC) curves of COCA to rank yeast cell cycle-related genes in (a) M/G1 phase, (b) G1 phase, (c) S phase, (d) G2 phase and (e) M phase as synchronized by Alpha.

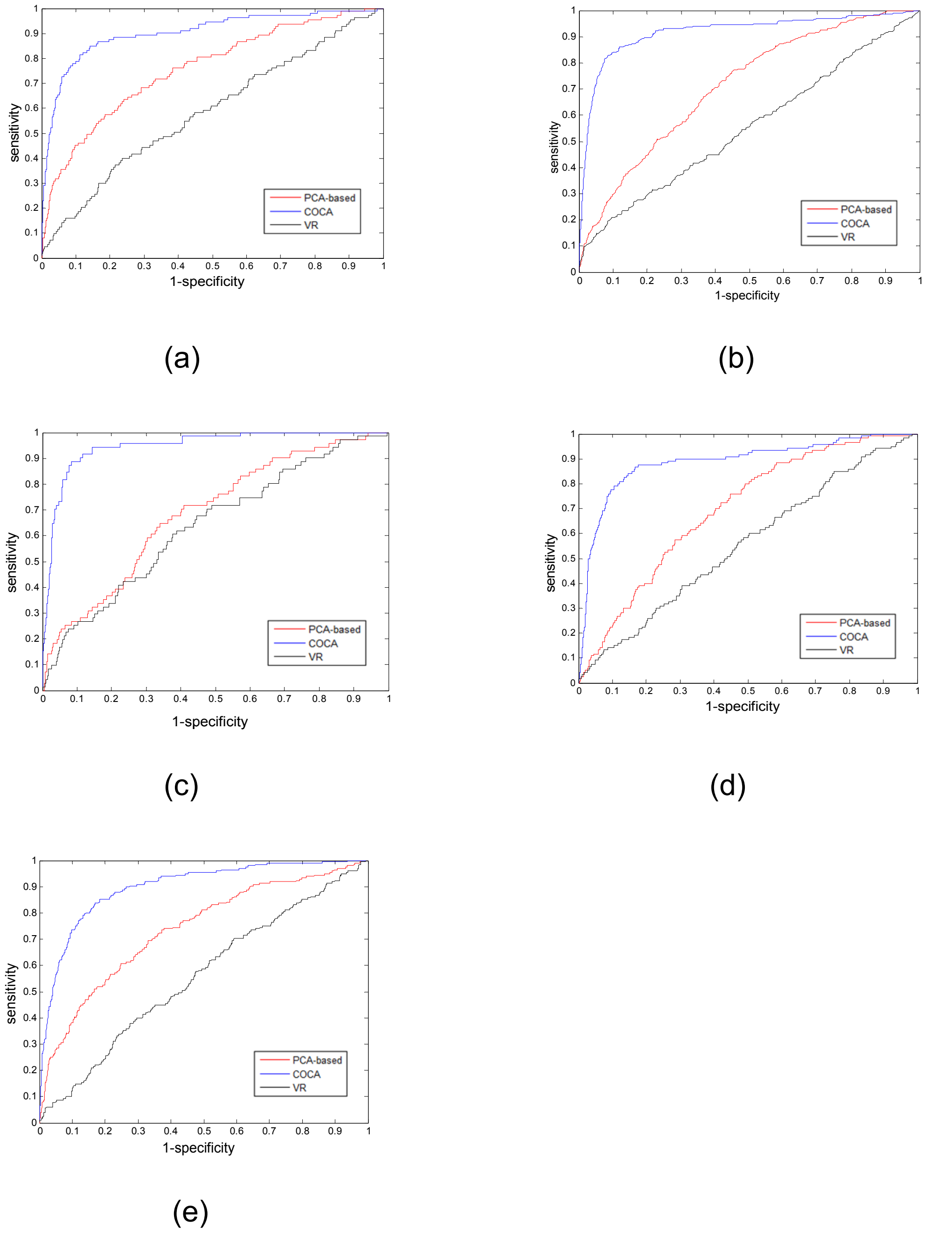

Figure S2. Receiver Operator Characteristic (ROC) curves of COCA to rank yeast cell cycle-related genes (a) M/G1 phase, (b) G1 phase, (c) S phase, (d) G2 phase and (e) M phase as synchronized by CDC15.

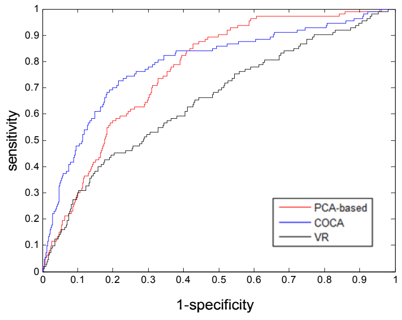

(a)

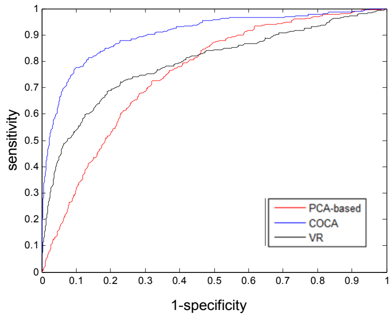

(b)

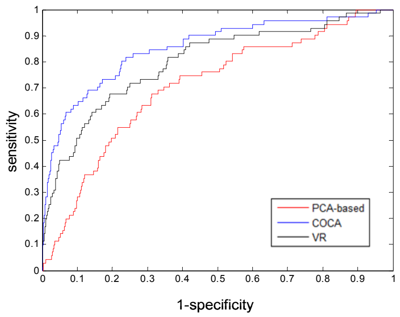

(c)

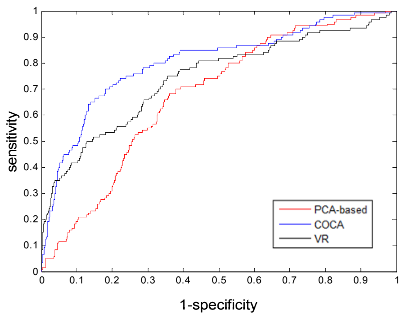

(d)

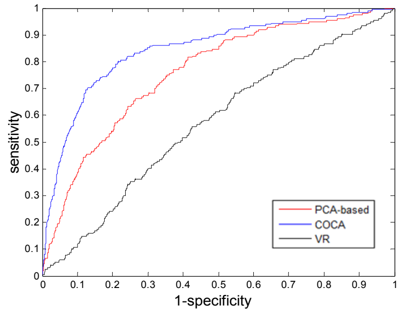

(e)

Figure S3. Receiver Operator Characteristic (ROC) curves of COCA to rank yeast cell cycle-related genes in (a) M/G1 phase, (b) G1 phase, (c) S phase, (d) G2 phase and (e) M phase as synchronized by CDC28.

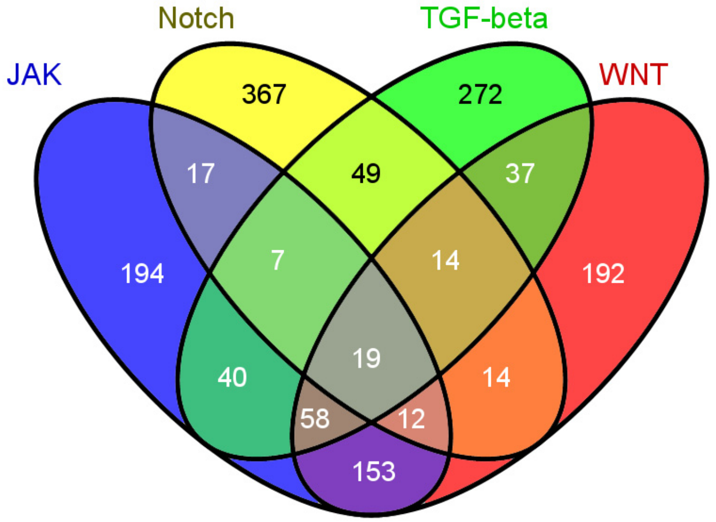

Figure S4. A Venn diagram of the top 500 probe sets ranked by the COCA approach guided by JAK, Notch, TGFβ and WNT pathways, respectively.

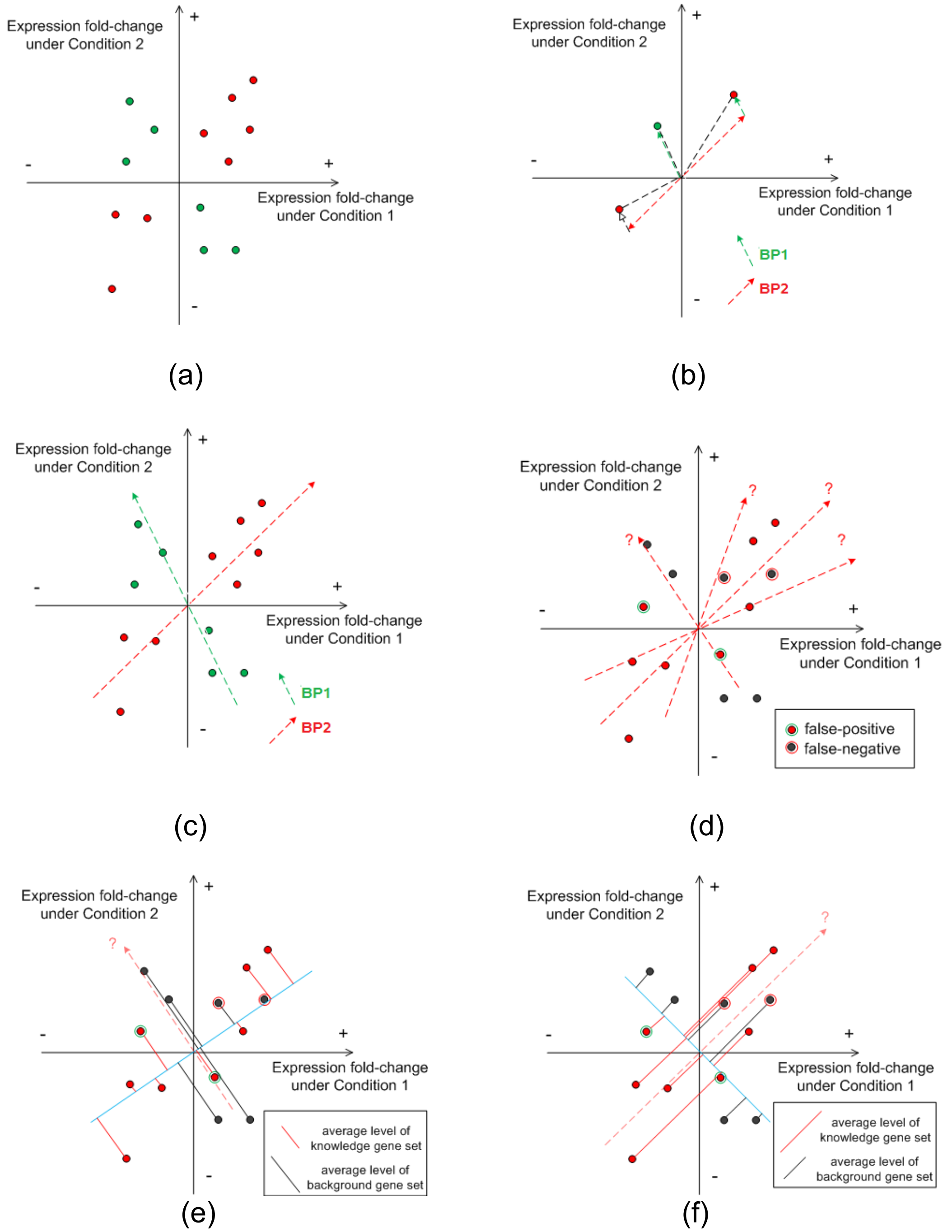

Figure S5. Geometrical understanding of COCA: (a) expression variation pattern; (b) underlying biological processes; (c) expression determined by underlying biological processes; (d) how to determine the direction; (e) a wrong guess of the direction; (f) a correct guess of the direction.
