# Supplementary material for: Knowledge-guided gene ranking by coordinative component analysis
Source: BMC Bioinformatics. 2010 Mar 30;11:162. doi: 10.1186/1471-2105-11-162 (PMC2865494; doi:10.1186/1471-2105-11-162)
Supplement: Additional file 2 — The top 500 probe sets ranked by Notch pathway-guided COCA approach. [file 1471-2105-11-162-S2.PDF]

Probe Set ID	Gene Symbol	Gene Name
1415708_at	Tug1	taurine upregulated gene 1
1415771_at	Ncl	nucleolin
1415773_at	Ncl	nucleolin
1415795_at	LOC100046080 /// Spin1	similar to Spindlin 1 /// spindlin 1
1415800_at	Gja1	gap junction protein, alpha 1
1415810_at	Uhrf1	ubiquitin-like, containing PHD and RING finger domains, 1
1415822_at	Scd2	stearoyl-Coenzyme A desaturase 2
1415823_at	Scd2	stearoyl-Coenzyme A desaturase 2
1415840_at	Elovl5	ELOVL family member 5, elongation of long chain fatty acids (yeast)
1415849_s_at	Stmn1	stathmin 1
1415863_at	Eif4g2	eukaryotic translation initiation factor 4, gamma 2
1415869_a_at	Trim28	tripartite motif-containing 28
1415918_a_at	Tpi1	triosephosphate isomerase 1
1415920_at	Cstf2t	cleavage stimulation factor, 3' pre-RNA subunit 2, tau
1415963_at	Hnrnph2	heterogeneous nuclear ribonucleoprotein H2
1415972_at	Marcks	myristoylated alanine rich protein kinase C substrate
1415988_at	Hdlbp	high density lipoprotein (HDL) binding protein
1415996_at	Txnip	thioredoxin interacting protein
1415997_at	Txnip	thioredoxin interacting protein
1416019_at	Dr1	down-regulator of transcription 1
1416041_at	Sgk1	serum/glucocorticoid regulated kinase 1
1416042_s_at	Nasp	nuclear autoantigenic sperm protein (histone-binding)
1416069_at	Pfkp	phosphofructokinase, platelet
1416105_at	Nnt	nicotinamide nucleotide transhydrogenase
1416131_s_at	Efr3a	EFR3 homolog A (S. cerevisiae)
1416156_at	Vcl	vinculin
1416214_at	Mcm4	minichromosome maintenance deficient 4 homolog (S. cerevisiae)
1416221_at	Fstl1	follistatin-like 1
1416252_at	Stk38	serine/threonine kinase 38
1416271_at	Perp	PERP, TP53 apoptosis effector
1416350_at	Klf16	Kruppel-like factor 16
1416401_at	Cd82	CD82 antigen
1416455_a_at	Cryab	crystallin, alpha B
1416488_at	Ccng2	cyclin G2
1416530_a_at	LOC100045567 /// Pnp1	similar to purine nucleoside phosphorylase /// purine-nucleoside phosphorylase 1
1416536_at	Mum1	melanoma associated antigen (mutated) 1

1416558_at	Melk	maternal embryonic leucine zipper kinase
1416589_at	Sparc	secreted acidic cysteine rich glycoprotein
1416617_at	Acss1	acyl-CoA synthetase short-chain family member 1
1416645_a_at	Afp	alpha fetoprotein
1416646_at	Afp	alpha fetoprotein
1416688_at	Snap91	synaptosomal-associated protein 91
1416749_at	Htra1	HtrA serine peptidase 1
1416759_at	Mical1	microtubule associated monoxygenase, calponin and LIM domain containing 1
1416800_at	Trpm7	transient receptor potential cation channel, subfamily M, member 7
1416846_a_at	Pdzrn3	PDZ domain containing RING finger 3
1416880_at	Mcl1	myeloid cell leukemia sequence 1
1416884_at	Cbx3	chromobox homolog 3 (Drosophila HP1 gamma)
1416915_at	Msh6	mutS homolog 6 (E. coli)
1416923_a_at	Bnip3l	BCL2/adenovirus E1B interacting protein 3-like
1416967_at	Sox2	SRY-box containing gene 2
1417057_a_at	Lamp3 /// LOC100043444 /// LOC100045251 /// Ppid	lysosomal-associated membrane protein 3 /// similar to peptidylprolyl isomerase D /// similar to peptidylprolyl isomerase D /// peptidylprolyl isomerase D (cyclophilin D)
1417065_at	Egr1	early growth response 1
1417155_at	Mycn	v-myc myelocytomatosis viral related oncogene, neuroblastoma derived (avian)
1417216_at	Pim2	proviral integration site 2
1417250_at	Rnf12	ring finger protein 12
1417356_at	Peg3	paternally expressed 3
1417392_a_at	Slc7a7	solute carrier family 7 (cationic amino acid transporter, y+ system), member 7
1417420_at	Ccnd1	cyclin D1
1417440_at	Arid1a	AT rich interactive domain 1A (SWI-like)
1417543_at	Rps6ka2	ribosomal protein S6 kinase, polypeptide 2
1417625_s_at	Cxcr7	chemokine (C-X-C motif) receptor 7
1417638_at	Lefty1	left right determination factor 1
1417654_at	Sdc4	syndecan 4
1417655_a_at	Ars2	arsenate resistance protein 2
1417749_a_at	Tjp1	tight junction protein 1
1417766_at	Cyb5b	cytochrome b5 type B
1417837_at	Phlda2	pleckstrin homology-like domain, family A, member 2
1417845_at	Cldn6	claudin 6

1417963_at	Pltp	phospholipid transfer protein
1418015_at	Pum2	pumilio 2 (Drosophila)
1418057_at	Tiam1	T-cell lymphoma invasion and metastasis 1
1418069_at	Apoc2	apolipoprotein C-II
1418078_at	Psme3	proteaseome (prosome, macropain) 28 subunit, 3
1418086_at	Ppp1r14a	protein phosphatase 1, regulatory (inhibitor) subunit 14A
1418115_s_at	Ifrg15	interferon alpha responsive gene
1418125_at	Ino80	INO80 homolog (S. cerevisiae)
1418152_at	Nsbp1	nucleosome binding protein 1
1418217_at	Nme7	non-metastatic cells 7, protein expressed in (nucleoside-diphosphate kinase)
1418362_at	Zfp42	zinc finger protein 42
1418370_at	Tnnc1	troponin C, cardiac/slow skeletal
1418380_at	Terf1	telomeric repeat binding factor 1
1418391_at	Phf21a	PHD finger protein 21A
1418502_a_at	Oxr1	oxidation resistance 1
1418507_s_at	Socs2	suppressor of cytokine signaling 2
1418520_at	LOC100038890 /// Tgoln1	hypothetical protein LOC100038890 /// trans-golgi network protein
1418563_at	Serbp1	Serpine1 mRNA binding protein 1
1418648_at	Egln3	EGL nine homolog 3 (C. elegans)
1418649_at	Egln3	EGL nine homolog 3 (C. elegans)
1418755_at	Tbx15	T-box 15
1418756_at	Trh	thyrotropin releasing hormone
1418797_at	Ms4a8a	membrane-spanning 4-domains, subfamily A, member 8A
1418918_at	Igfbp1	insulin-like growth factor binding protein 1
1419010_x_at	Klk1b5	kallikrein 1-related peptidase b5
1419021_at	Mcf2	mcf.2 transforming sequence
1419031_at	Fads2	fatty acid desaturase 2
1419086_at	Fgfbp1	fibroblast growth factor binding protein 1
1419095_a_at	Apom	apolipoprotein M
1419112_at	LOC100044468 /// Nlk	similar to nemo-like kinase /// nemo like kinase
1419232_a_at	Apoa1	apolipoprotein A-I
1419233_x_at	Apoa1	apolipoprotein A-I
1419350_at	Hook2	hook homolog 2 (Drosophila)
1419389_at	Pde10a	phosphodiesterase 10A
1419440_at	Trim54	tripartite motif-containing 54
1419452_at	Uchl5	ubiquitin carboxyl-terminal esterase L5
1419574_at	Zfp292	zinc finger protein 292
1419879_s_at	Trim25	tripartite motif-containing 25
1420088_at	Nfkbia	nuclear factor of kappa light polypeptide gene enhancer in B-cells inhibitor, alpha
1420441_at	Cenpc1	centromere protein C1

1420476_a_at	100043064 /// Nap1l1	predicted gene, 100043064 /// nucleosome assembly protein 1-like 1
1420478_at	Nap1l1	nucleosome assembly protein 1-like 1
1420494_x_at	Gm1821 /// LOC100048105 /// Rps27a /// Ubb /// Ubc	gene model 1821, (NCBI) /// similar to Ubc protein /// ribosomal protein S27a /// ubiquitin B /// ubiquitin C
1420549_at	Gbp1	guanylate binding protein 1
1420621_a_at	App	amyloid beta (A4) precursor protein
1420719_at	Tex15	testis expressed gene 15
1420884_at	Sln	sarcolipin
1420901_a_at	Hk1	hexokinase 1
1420916_at	Prpf40a	PRP40 pre-mRNA processing factor 40 homolog A (yeast)
1420922_at	Usp9x	ubiquitin specific peptidase 9, X chromosome
1420928_at	St6gal1	beta galactoside alpha 2,6 sialyltransferase 1
1420952_at	Son	Son DNA binding protein
1420968_at	Btbd14b	BTB (POZ) domain containing 14B
1421106_at	Jag1	jagged 1
1421130_at	Zfp111	zinc finger protein 111
1421330_at	Ptpn4	protein tyrosine phosphatase, non-receptor type 4
1421375_a_at	S100a6	S100 calcium binding protein A6 (calcyclin)
1421668_x_at	Speer3	spermatogenesis associated glutamate (E)-rich protein 3
1421754_at	AY036118	cDNA sequence AY036118
1421811_at	LOC640441 /// Thbs1	similar to thrombospondin 1 /// thrombospondin 1
1421821_at	Ldlr	low density lipoprotein receptor
1421872_at	Rab24	RAB24, member RAS oncogene family
1421882_a_at	Elavl2	ELAV (embryonic lethal, abnormal vision, Drosophila)-like 2 (Hu antigen B)
1421883_at	Elavl2	ELAV (embryonic lethal, abnormal vision, Drosophila)-like 2 (Hu antigen B)
1421964_at	Notch3	Notch gene homolog 3 (Drosophila)
1421965_s_at	Notch3	Notch gene homolog 3 (Drosophila)
1421967_at	B4galt5	UDP-Gal:betaGlcNAc beta 1,4-galactosyltransferase, polypeptide 5
1421983_s_at	Hnf4a	hepatic nuclear factor 4, alpha
1422045_a_at	Ptpn12	protein tyrosine phosphatase, non-receptor type 12
1422122_at	Fcer2a	Fc receptor, IgE, low affinity II, alpha polypeptide
1422138_at	Plau	plasminogen activator, urokinase
1422142_at	Nphs1	nephrosis 1 homolog, nephrin (human)
1422557_s_at	Mt1	metallothionein 1
1422612_at	Hk2	hexokinase 2
1422621_at	Ranbp2	RAN binding protein 2
1422734_a_at	Myb	myeloblastosis oncogene

1422902_s_at	Mgea5	meningioma expressed antigen 5 (hyaluronidase)
1423050_s_at	Hnrnpu	heterogeneous nuclear ribonucleoprotein U
1423051_at	Hnrnpu	heterogeneous nuclear ribonucleoprotein U
1423078_a_at	Sc4mol	sterol-C4-methyl oxidase-like
1423130_a_at	Sfrs5	splicing factor, arginine/serine-rich 5 (SRp40, HRS)
1423222_at	Cap2	CAP, adenylate cyclase-associated protein, 2 (yeast)
1423228_at	B4galt6 /// LOC675709	UDP-Gal:betaGlcNAc beta 1,4-galactosyltransferase, polypeptide 6 /// similar to Beta-1,4-galactosyltransferase 6 (Beta-1,4-GalTase 6) (Beta4Gal-T6) (b4Gal-T6) (UDP-galactose:beta-N-acetylglucosamine beta-1,4-galactosyltransferase 6) (UDP-Gal:beta-GlcNAc beta-1,4-galactosyltransferase 6)
1423294_at	Mest	mesoderm specific transcript
1423331_a_at	LOC100047693 /// Pvrl3	similar to cell adhesion molecule nectin-3 beta /// poliovirus receptor-related 3
1423358_at	Ece2	endothelin converting enzyme 2
1423380_s_at	Nfatc4	nuclear factor of activated T-cells, cytoplasmic, calcineurin-dependent 4
1423474_at	Top1	topoisomerase (DNA) I
1423491_at	Fbxo3	F-box protein 3
1423504_at	Jam3	junction adhesion molecule 3
1423505_at	Tagln	transgelin
1423508_at	Myst4	MYST histone acetyltransferase monocytic leukemia 4
1423532_at	Rnf44	ring finger protein 44
1423566_a_at	Hsph1	heat shock 105kDa/110kDa protein 1
1423645_a_at	Ddx5	DEAD (Asp-Glu-Ala-Asp) box polypeptide 5
1423667_at	Mat2a	methionine adenosyltransferase II, alpha
1423740_a_at	Rbm10	RNA binding motif protein 10
1423747_a_at	Pdk1	pyruvate dehydrogenase kinase, isoenzyme 1
1423750_a_at	Sf1	splicing factor 1
1423795_at	Sfpq	splicing factor proline/glutamine rich (polypyrimidine tract binding protein associated)
1423804_a_at	Idi1	isopentenyl-diphosphate delta isomerase
1423828_at	Fasn	fatty acid synthase
1423878_at	Gypc	glycophorin C
1423899_at	Trip12	thyroid hormone receptor interactor 12
1423900_at	Trip12	thyroid hormone receptor interactor 12

1423905_at	Pvr	poliovirus receptor
1423961_at	Wdr26	WD repeat domain 26
1424050_s_at	Fgfr1	fibroblast growth factor receptor 1
1424075_at	9430016H08Rik	RIKEN cDNA 9430016H08 gene
1424211_at	Slc25a33	solute carrier family 25, member 33
1424353_at	Lrpprc	leucine-rich PPR-motif containing
1424390_at	Nupl1	nucleoporin like 1
1424398_at	Dhx36	DEAH (Asp-Glu-Ala-His) box polypeptide 36
1424450_at	Gprc5c	G protein-coupled receptor, family C, group 5, member C
1424528_at	Cgref1	cell growth regulator with EF hand domain 1
1424641_a_at	Thoc1	THO complex 1
1424702_a_at	Atg2b	ATG2 autophagy related 2 homolog B (S. cerevisiae)
1424723_s_at	Cstf3	cleavage stimulation factor, 3' pre-RNA, subunit 3
1424740_at	Creb3	cAMP responsive element binding protein 3
1424752_x_at	Zfp71-rs1	zinc finger protein 71, releated sequence
1424759_at	Arrdc4	arrestin domain containing 4
1424797_a_at	Pitx2	paired-like homeodomain transcription factor 2
1424826_s_at	Mtss1	metastasis suppressor 1
1425332_at	Zfp106	zinc finger protein 106
1425469_a_at	---	---
1425498_at	Prpf4b	PRP4 pre-mRNA processing factor 4 homolog B (yeast)
1425577_at	Zmym5	zinc finger, MYM-type 5
1425617_at	Dhx9	DEAH (Asp-Glu-Ala-His) box polypeptide 9
1425628_a_at	Gtf2i	general transcription factor II I
1425652_s_at	LOC100044395 /// Rbpms	similar to RNA binding protein gene with multiple splicing /// RNA binding protein gene with multiple splicing
1425717_at	Lrba	LPS-responsive beige-like anchor
1425718_a_at	Ivns1abp	influenza virus NS1A binding protein
1425911_a_at	Fgfr1	fibroblast growth factor receptor 1
1426084_a_at	Tor1aip1	torsin A interacting protein 1
1426148_at	Gbgt1	globoside alpha-1,3-N-acetylgalactosaminyltransferase 1
1426191_a_at	Bcl2l1	BCL2-like 1
1426225_at	Rbp4	retinol binding protein 4, plasma

1426260_a_at	LOC632297 /// Ugt1a1 /// Ugt1a10 /// Ugt1a2 /// Ugt1a5 /// Ugt1a6a /// Ugt1a6b /// Ugt1a7c /// Ugt1a9	similar to UDP glycosyltransferase 1 family, polypeptide A8 /// UDP glucuronosyltransferase 1 family, polypeptide A1 /// UDP glycosyltransferase 1 family, polypeptide A10 /// UDP glucuronosyltransferase 1 family, polypeptide A2 /// UDP glucuronosyltransferase 1 family, polypeptide A5 /// UDP glucuronosyltransferase 1 family, polypeptide A6A /// UDP glucuronosyltransferase 1 family, polypeptide A6B /// UDP glucuronosyltransferase 1 family, polypeptide A7C /// UDP glucuronosyltransferase 1 family, polypeptide A9
1426262_at	Adnp2	ADNP homeobox 2
1426371_at	Far1	fatty acyl CoA reductase 1
1426411_a_at	C230082I21Rik /// Strbp	RIKEN cDNA C230082I21 gene /// spermatid perinuclear RNA binding protein
1426430_at	Jag2	jagged 2
1426469_a_at	Tbp	TATA box binding protein
1426631_at	LOC100047009 /// Pus7	hypothetical protein LOC100047009 /// pseudouridylate synthase 7 homolog (S. cerevisiae)
1426642_at	Fn1	fibronectin 1
1426664_x_at	Slc45a3	solute carrier family 45, member 3
1426674_at	Eif3b	eukaryotic translation initiation factor 3, subunit B
1426698_a_at	Hnrnpm	heterogeneous nuclear ribonucleoprotein M
1426730_a_at	Prl2b1	prolactin family 2, subfamily b, member 1
1426736_at	Gspt1	G1 to S phase transition 1
1426746_at	1810026J23Rik	RIKEN cDNA 1810026J23 gene
1426783_at	Kat2a	K(lysine) acetyltransferase 2A
1426817_at	Mki67	antigen identified by monoclonal antibody Ki 67
1426853_at	671392 /// Set	predicted gene, 671392 /// SET translocation
1426854_a_at	671392 /// LOC100047898 /// Set	predicted gene, 671392 /// similar to protein phosphatase 2A inhibitor-2 I-2PP2A /// SET translocation
1426880_at	Etl4	enhancer trap locus 4
1426909_at	Uck2	uridine-cytidine kinase 2
1426936_at	629242 /// BC005512 /// EG641366 /// LOC215866	predicted gene, 629242 /// cDNA sequence BC005512 /// predicted gene, EG641366 /// hypothetical protein LOC215866

1426946_at	Ipo5	importin 5
1427131_s_at	Lrrc58	leucine rich repeat containing 58
1427143_at	Jarid1b	jumonji, AT rich interactive domain 1B (Rbp2 like)
1427171_at	Rlf	rearranged L-myc fusion sequence
1427260_a_at	Tpm3	tropomyosin 3, gamma
1427266_at	Pbrm1	polybromo 1
1427275_at	Smc4	structural maintenance of chromosomes 4
1427310_at	Bptf	bromodomain PHD finger transcription factor
1427825_at	---	---
1427873_at	Defcr15	defensin related cryptdin 15
1427902_at	LOC100046744 /// Srrm2	similar to Serine/arginine repetitive matrix protein 2 /// serine/arginine repetitive matrix 2
1427949_at	Zfp294	zinc finger protein 294
1427965_at	Ssbp1	single-stranded DNA binding protein 1
1428094_at	Lamp2	lysosomal-associated membrane protein 2
1428111_at	Slc38a4	solute carrier family 38, member 4
1428193_at	Usp9x	ubiquitin specific peptidase 9, X chromosome
1428194_at	Usp9x	ubiquitin specific peptidase 9, X chromosome
1428280_at	Fip1l1	FIP1 like 1 (S. cerevisiae)
1428286_at	2900097C17Rik	RIKEN cDNA 2900097C17 gene
1428306_at	Ddit4	DNA-damage-inducible transcript 4
1428389_s_at	Wdr43	WD repeat domain 43
1428402_at	Zcchc3	zinc finger, CCHC domain containing 3
1428510_at	Lphn1	latrophilin 1
1428662_a_at	Hopx	HOP homeobox
1428847_a_at	Macf1	microtubule-actin crosslinking factor 1
1428850_x_at	Cd99	CD99 antigen
1428853_at	Ptch1	patched homolog 1
1428869_at	Nolc1	nucleolar and coiled-body phosphoprotein 1
1428870_at	Nolc1	nucleolar and coiled-body phosphoprotein 1
1428942_at	Mt2	metallothionein 2
1429359_s_at	Rbpms	RNA binding protein gene with multiple splicing
1429491_s_at	Rif1	Rap1 interacting factor 1 homolog (yeast)
1429888_a_at	Hspb2	heat shock protein 2
1430514_a_at	Cd99	CD99 antigen
1430692_a_at	Sel1l	sel-1 suppressor of lin-12-like (C. elegans)
1431701_a_at	Pdzk1	PDZ domain containing 1
1431939_a_at	Mina	myc induced nuclear antigen

1433443_a_at	Hmgcs1 /// LOC100040592	3-hydroxy-3-methylglutaryl-Coenzyme A synthase 1 /// similar to Hmgcs1 protein
1433444_at	Hmgcs1 /// LOC100040592	3-hydroxy-3-methylglutaryl-Coenzyme A synthase 1 /// similar to Hmgcs1 protein
1433446_at	Hmgcs1 /// LOC100040592	3-hydroxy-3-methylglutaryl-Coenzyme A synthase 1 /// similar to Hmgcs1 protein
1433486_at	Clcn3	chloride channel 3
1433488_x_at	Gns	glucosamine (N-acetyl)-6-sulfatase
1433519_at	Nucks1	nuclear casein kinase and cyclin-dependent kinase substrate 1
1433531_at	Acsl4	acyl-CoA synthetase long-chain family member 4
1433534_a_at	Cct2	chaperonin containing Tcp1, subunit 2 (beta)
1433540_x_at	LOC100044953 /// Ppp1cb	similar to protein phosphatase 1 /// protein phosphatase 1, catalytic subunit, beta isoform
1433552_a_at	Polr2b	polymerase (RNA) II (DNA directed) polypeptide B
1433576_at	Mat2a	methionine adenosyltransferase II, alpha
1433581_at	1190002N15Rik /// LOC100044725	RIKEN cDNA 1190002N15 gene /// hypothetical protein LOC100044725
1433631_at	Eif5	eukaryotic translation initiation factor 5
1433676_at	Wnk1	WNK lysine deficient protein kinase 1
1433809_at	Ddx5	DEAD (Asp-Glu-Ala-Asp) box polypeptide 5
1433892_at	Spag5	sperm associated antigen 5
1434011_a_at	Ints5	integrator complex subunit 5
1434038_at	Dnajc13	DnaJ (Hsp40) homolog, subfamily C, member 13
1434047_x_at	Hnrnpa2b1	heterogeneous nuclear ribonucleoprotein A2/B1
1434148_at	Tcf4	transcription factor 4
1434215_at	B230308N11Rik	RIKEN cDNA B230308N11 gene
1434278_at	Mtm1	X-linked myotubular myopathy gene 1
1434487_at	Mef2d	myocyte enhancer factor 2D
1434503_s_at	Lamp2	lysosomal-associated membrane protein 2
1434578_x_at	LOC100045999 /// Ran	similar to RAN, member RAS oncogene family /// RAN, member RAS oncogene family
1434644_at	Tbl1x	transducin (beta)-like 1 X-linked
1434705_at	Ctbp2	C-terminal binding protein 2
1434853_x_at	Mkrn1	makorin, ring finger protein, 1
1434884_at	Mtdh	Metadherin
1434920_a_at	Evl /// LOC100047333	Ena-vasodilator stimulated phosphoprotein /// similar to Ena-VASP-like

1434972_x_at	LOC100048559 /// Sfrs1	similar to splicing factor, arginine/serine-rich 1 (splicing factor 2, alternate splicing factor) /// splicing factor, arginine/serine-rich 1 (ASF/SF2)
1435086_s_at	Klhdc2	kelch domain containing 2
1435140_at	Ide	insulin degrading enzyme
1435194_at	Hspa4	heat shock protein 4
1435335_a_at	Gnptab	N-acetylglucosamine-1-phosphate transferase, alpha and beta subunits
1435494_s_at	Dsp	desmoplakin
1435702_s_at	Ywhae	tyrosine 3-monooxygenase/tryptophan 5-monooxygenase activation protein, epsilon polypeptide
1435989_x_at	Krt8	keratin 8
1436298_x_at	Paics	phosphoribosylaminoimidazole carboxylase, phosphoribosylaminoribosylaminoimidazole, succinocarboxamide synthetase
1436308_at	Zfp292	zinc finger protein 292
1436392_s_at	Tcfap2c	transcription factor AP-2, gamma
1436584_at	Spry2	sprouty homolog 2 (Drosophila)
1436703_x_at	Snapc2	small nuclear RNA activating complex, polypeptide 2
1436714_at	Lpp	LIM domain containing preferred translocation partner in lipoma
1436736_x_at	D0H4S114	DNA segment, human D4S114
1436879_x_at	Afp	alpha fetoprotein
1436898_at	Sfpq	splicing factor proline/glutamine rich (polypyrimidine tract binding protein associated)
1436926_at	Esrrb	estrogen related receptor, beta
1437052_s_at	Slc2a3	solute carrier family 2 (facilitated glucose transporter), member 3
1437238_x_at	Nmd3	NMD3 homolog (S. cerevisiae)
1437267_x_at	Hnrnph1	Heterogeneous nuclear ribonucleoprotein H1
1437278_a_at	Uba2	ubiquitin-like modifier activating enzyme 2
1437405_a_at	Igfbp4	insulin-like growth factor binding protein 4
1437455_a_at	Btg1 /// LOC100047353	B-cell translocation gene 1, anti-proliferative /// similar to myocardial vascular inhibition factor
1437458_x_at	Clu	clusterin
1437608_x_at	Ywhaq	tyrosine 3-monooxygenase/tryptophan 5-monooxygenase activation protein, theta polypeptide
1437626_at	Zfp36l2	zinc finger protein 36, C3H type-like 2
1437689_x_at	Clu	clusterin
1437719_x_at	A230046K03Rik	RIKEN cDNA A230046K03 gene
1437721_at	Coro1c	coronin, actin binding protein 1C

1437773_x_at	Ddx17	DEAD (Asp-Glu-Ala-Asp) box polypeptide 17
1437810_a_at	Hbb-bh1 /// LOC100044263	hemoglobin Z, beta-like embryonic chain /// hypothetical protein LOC100044263
1437835_a_at	0610011L14Rik	RIKEN cDNA 0610011L14 gene
1437841_x_at	Csdc2	cold shock domain containing C2, RNA binding
1437843_s_at	Nupl1	nucleoporin like 1
1437974_a_at	Hk1	hexokinase 1
1437990_x_at	Hbb-bh1 /// LOC100044263	hemoglobin Z, beta-like embryonic chain /// hypothetical protein LOC100044263
1437992_x_at	Gja1	gap junction protein, alpha 1
1437993_x_at	Qdpr	quinoid dihydropteridine reductase
1438092_x_at	H2afz	H2A histone family, member Z
1438096_a_at	Dtymk	deoxythymidylate kinase
1438118_x_at	Vim	vimentin
1438292_x_at	Adk	adenosine kinase
1438354_x_at	Cnn3	Calponin 3, acidic
1438360_x_at	EG383528 /// EG433326 /// Slc25a5	predicted gene, EG383528 /// predicted gene, EG433326 /// solute carrier family 25 (mitochondrial carrier, adenine nucleotide translocator), member 5
1438371_x_at	Ddx5	DEAD (Asp-Glu-Ala-Asp) box polypeptide 5
1438549_a_at	Srr	serine racemase
1438630_x_at	Mat2a	methionine adenosyltransferase II, alpha
1438631_x_at	Ttc13	tetratricopeptide repeat domain 13
1438637_x_at	Sf3b2	splicing factor 3b, subunit 2
1438651_a_at	Aplnr	apelin receptor
1438695_at	C230091D08Rik	RIKEN cDNA C230091D08 gene
1438825_at	Calm3	Calmodulin 3
1438840_x_at	Apoa1	apolipoprotein A-I
1438852_x_at	Mcm6	minichromosome maintenance deficient 6 (MIS5 homolog, S. pombe) (S. cerevisiae)
1439065_x_at	OTTMUSG00000010173	Predicted gene, OTTMUSG00000010173
1439148_a_at	Pfkl	phosphofructokinase, liver, B-type
1439380_x_at	Meg3	maternally expressed 3
1439411_a_at	Xpo7	exportin 7
1439435_x_at	Pgk1	phosphoglycerate kinase 1
1439438_a_at	1110005A23Rik /// EG625193	RIKEN cDNA 1110005A23 gene /// predicted gene, EG625193
1439439_x_at	Eef1d	eukaryotic translation elongation factor 1 delta (guanine nucleotide exchange protein)
1439440_x_at	Twf2	twinfilin, actin-binding protein, homolog 2 (Drosophila)

1439464_s_at	Tex10	testis expressed gene 10
1440195_at	Serbp1	Serpine1 mRNA binding protein 1
1440831_at	Bach1	BTB and CNC homology 1
1442554_s_at	Kalrn	kalirin, RhoGEF kinase
1442661_at	Dhrs7b	dehydrogenase/reductase (SDR family) member 7B
1443856_at	Rabep1	rabaptin, RAB GTPase binding effector protein 1
1443892_at	EG435970 /// LOC100045488	predicted gene, EG435970 /// similar to crooked legs CG14938-PB
1444952_a_at	Nucks1	nuclear casein kinase and cyclin-dependent kinase substrate 1
1446086_s_at	Gli2	GLI-Kruppel family member GLI2
1447977_x_at	3100002L24Rik /// OTTMUSG00000016609 /// OTTMUSG00000016611	RIKEN cDNA 3100002L24 gene /// predicted gene, OTTMUSG00000016609 /// predicted gene, OTTMUSG00000016611
1448152_at	Igf2	insulin-like growth factor 2
1448201_at	Sfrp2	secreted frizzled-related protein 2
1448207_at	Lasp1	LIM and SH3 protein 1
1448219_a_at	Ywhaz	tyrosine 3-monooxygenase/tryptophan 5-monooxygenase activation protein, zeta polypeptide
1448226_at	Rrm2	ribonucleotide reductase M2
1448270_at	Ddx21	DEAD (Asp-Glu-Ala-Asp) box polypeptide 21
1448289_at	Crmp1	collapsin response mediator protein 1
1448347_a_at	Caprin1	cell cycle associated protein 1
1448396_at	Tmem131	transmembrane protein 131
1448519_at	Tead2	TEA domain family member 2
1448552_s_at	Tmem206	transmembrane protein 206
1448609_at	Tst	thiosulfate sulfurtransferase, mitochondrial
1448619_at	Dhcr7	7-dehydrocholesterol reductase
1448709_at	Arid1a	AT rich interactive domain 1A (SWI-like)
1448757_at	Pml	promyelocytic leukemia
1448890_at	Klf2	Kruppel-like factor 2 (lung)
1448893_at	Ncor2	nuclear receptor co-repressor 2
1448964_at	S100g	S100 calcium binding protein G
1448970_at	Slc25a46	solute carrier family 25, member 46
1448992_at	Ina /// LOC100047943	internexin neuronal intermediate filament protein, alpha /// similar to Ina protein
1449005_at	Slc16a3	solute carrier family 16 (monocarboxylic acid transporters), member 3
1449050_at	Rfc1	replication factor C (activator 1) 1
1449052_a_at	Dnmt3b	DNA methyltransferase 3B
1449056_at	E330009J07Rik	RIKEN cDNA E330009J07 gene
1449080_at	Hdac2	histone deacetylase 2
1449188_at	Midn	midnolin

1449240_at	Gsbs	G substrate
1449315_at	Odz3	odd Oz/ten-m homolog 3 (Drosophila)
1449456_a_at	Cma1	chymase 1, mast cell
1449482_at	Hist3h2ba	histone cluster 3, H2ba
1449510_at	Zfp467	zinc finger protein 467
1449614_s_at	AI314976	expressed sequence AI314976
1449675_at	231869 /// Ccnb1 /// Ccnb1-rs1 /// EG434175 /// EG667005 /// LOC635091	predicted gene, 231869 /// cyclin B1 /// cyclin B1, related sequence 1 /// predicted gene, EG434175 /// predicted gene, EG667005 /// similar to major urinary protein 5
1449773_s_at	Gadd45b	growth arrest and DNA-damage-inducible 45 beta
1449836_x_at	Bik	BCL2-interacting killer
1449939_s_at	Dlk1	delta-like 1 homolog (Drosophila)
1450012_x_at	Ywhag	tyrosine 3-monooxygenase/tryptophan 5-monooxygenase activation protein, gamma polypeptide
1450021_at	Ubqln2	ubiquilin 2
1450031_at	Aff4	AF4/FMR2 family, member 4
1450052_at	Kif2a	kinesin family member 2A
1450059_at	Fancg	Fanconi anemia, complementation group G
1450082_s_at	Etv5	ets variant gene 5
1450084_s_at	Ivns1abp	influenza virus NS1A binding protein
1450269_a_at	Pfkl	phosphofructokinase, liver, B-type
1450350_a_at	Jdp2	Jun dimerization protein 2
1450636_s_at	Akp5	alkaline phosphatase 5
1450650_at	Myo10	myosin X
1450690_at	Ranbp2	RAN binding protein 2
1450710_at	Jarid2	jumonji, AT rich interactive domain 2
1450736_a_at	Hbb-bh1 /// LOC100044263	hemoglobin Z, beta-like embryonic chain /// hypothetical protein LOC100044263
1450744_at	Ell2	elongation factor RNA polymerase II 2
1450746_at	Keap1	kelch-like ECH-associated protein 1
1450764_at	Aoah	acyloxyacyl hydrolase
1450870_at	Rala	v-ral simian leukemia viral oncogene homolog A (ras related)
1450937_at	Lin7c	lin-7 homolog C (C. elegans)
1450950_at	Smc3	structural maintenace of chromosomes 3
1450971_at	Gadd45b	growth arrest and DNA-damage-inducible 45 beta
1450989_at	Tdgf1	teratocarcinoma-derived growth factor 1
1451179_a_at	LOC100046895 /// Qk	similar to Quaking protein /// quaking
1451184_at	EG627828 /// Hnrnpa3 /// LOC100045099 /// OTTMUSG00000004599	predicted gene, EG627828 /// heterogeneous nuclear ribonucleoprotein A3 /// similar to heterogeneous nuclear ribonucleoprotein A3 /// predicted gene, OTTMUSG00000004599

1451220_at	Wdr20a	WD repeat domain 20a
1451254_at	Ikbkap	inhibitor of kappa light polypeptide enhancer in B-cells, kinase complex-associated protein
1451285_at	Fus	fusion, derived from t(12;16) malignant liposarcoma (human)
1451286_s_at	Fus	fusion, derived from t(12;16) malignant liposarcoma (human)
1451316_a_at	Picalm	phosphatidylinositol binding clathrin assembly protein
1451416_a_at	Tgm1	transglutaminase 1, K polypeptide
1451527_at	Pcolce2	procollagen C-endopeptidase enhancer 2
1451554_a_at	Aph1a	anterior pharynx defective 1a homolog (C. elegans)
1451730_at	Zfp62	zinc finger protein 62
1451776_s_at	Hopx	HOP homeobox
1451872_a_at	Neurl	neuralized-like homolog (Drosophila)
1451913_a_at	Hyou1	hypoxia up-regulated 1
1452155_a_at	Ddx17	DEAD (Asp-Glu-Ala-Asp) box polypeptide 17
1452195_s_at	Sfi1	Sfi1 homolog, spindle assembly associated (yeast)
1452209_at	Pkp4	plakophilin 4
1452222_at	Utrn	utrophin
1452232_at	Galnt7	UDP-N-acetyl-alpha-D-galactosamine: polypeptide N-acetylgalactosaminyltransferase 7
1452265_at	Clasp1	CLIP associating protein 1
1452276_at	Smarcad1	SWI/SNF-related, matrix-associated actin-dependent regulator of chromatin, subfamily a, containing DEAD/H box 1
1452377_at	Mll1	myeloid/lymphoid or mixed-lineage leukemia 1
1452387_a_at	Amotl2	angiomotin-like 2
1452438_s_at	LOC100046932 /// Taf4a	similar to TAF4A RNA polymerase II, TATA box binding protein (TBP)-associated factor /// TAF4A RNA polymerase II, TATA box binding protein (TBP)-associated factor
1452659_at	Dek	DEK oncogene (DNA binding)
1452670_at	Myl9	myosin, light polypeptide 9, regulatory
1453223_s_at	Dppa2	developmental pluripotency associated 2
1453470_a_at	Gna13	guanine nucleotide binding protein, alpha 13
1453556_x_at	Cd99	CD99 antigen
1453733_a_at	4933434E20Rik	RIKEN cDNA 4933434E20 gene
1454607_s_at	Psat1	phosphoserine aminotransferase 1
1454608_x_at	Ttr	transthyretin
1454616_at	Ubr7	ubiquitin protein ligase E3 component n-recognin 7 (putative)

1454628_at	A930037G23Rik	RIKEN cDNA A930037G23 gene
1454636_at	Cbx5	chromobox homolog 5 (Drosophila HP1a)
1454794_at	Spast	spastin
1454803_a_at	Hdac11	histone deacetylase 11
1454831_at	Foxn2	forkhead box N2
1454849_x_at	Clu	clusterin
1454890_at	Amot	angiomotin
1454947_a_at	Ublcp1	ubiquitin-like domain containing CTD phosphatase 1
1454971_x_at	Tsc22d1	TSC22 domain family, member 1
1455201_x_at	Apoa1	apolipoprotein A-I
1455202_at	Gm50 /// LOC100045737	gene model 50, (NCBI) /// hypothetical protein LOC100045737
1455204_at	Pitpnc1	phosphatidylinositol transfer protein, cytoplasmic 1
1455899_x_at	Socs3	suppressor of cytokine signaling 3
1455913_x_at	Ttr	transthyretin
1455930_at	---	---
1455956_x_at	Ccnd2	cyclin D2
1456014_s_at	Fermt3	fermitin family homolog 3 (Drosophila)
1456032_x_at	EG666634 /// H2afz	predicted gene, EG666634 /// H2A histone family, member Z
1456054_a_at	Pum1	pumilio 1 (Drosophila)
1456112_at	LOC100043998 /// Tpr	similar to nuclear pore complex-associated intranuclear coiled-coil protein TPR /// translocated promoter region
1456243_x_at	Mcl1	myeloid cell leukemia sequence 1
1456310_a_at	2610002J02Rik	RIKEN cDNA 2610002J02 gene
1456377_x_at	Limd2	LIM domain containing 2
1456424_s_at	Pltp	phospholipid transfer protein
1456573_x_at	Nnt	nicotinamide nucleotide transhydrogenase
1456578_x_at	Lasp1	LIM and SH3 protein 1
1456590_x_at	Akr1b3	aldo-keto reductase family 1, member B3 (aldose reductase)
1456664_x_at	Hnrnpf /// OTTMUSG00000004294	heterogeneous nuclear ribonucleoprotein F /// predicted gene, OTTMUSG00000004294
1456728_x_at	Aco1	aconitase 1
1460206_at	Grasp	GRP1 (general receptor for phosphoinositides 1)-associated scaffold protein
1460257_a_at	100039707 /// Mthfs	predicted gene, 100039707 /// 5, 10-methenyltetrahydrofolate synthetase
1460317_s_at	Gna13	guanine nucleotide binding protein, alpha 13
1460325_at	Pum1	pumilio 1 (Drosophila)
1460403_at	Psip1	PC4 and SFRS1 interacting protein 1

1460544_at	Mak10	MAK10 homolog, amino-acid N-acetyltransferase subunit, (S. cerevisiae)
1460547_a_at	Hnrnpk	heterogeneous nuclear ribonucleoprotein K
1460571_at	Dicer1	Dicer1, Dcr-1 homolog (Drosophila)
1460631_at	Ogt	O-linked N-acetylglucosamine (GlcNAc) transferase (UDP-N-acetylglucosamine:polypeptide-N-acetylglucosaminyl transferase)
