# Supplementary material for: Knowledge-guided gene ranking by coordinative component analysis
Source: BMC Bioinformatics. 2010 Mar 30;11:162. doi: 10.1186/1471-2105-11-162 (PMC2865494; doi:10.1186/1471-2105-11-162)
Supplement: Additional file 3 — The top 500 probe sets ranked by JAK/STAT pathway-guided COCA approach. [file 1471-2105-11-162-S3.PDF]

Probe Set ID	Gene Symbol	Gene Name
1415708_at	Tug1	taurine upregulated gene 1
1415806_at	Plat	plasminogen activator, tissue
1415808_at	Tpbpa	trophoblast specific protein alpha
1415812_at	Gsn	gelsolin
1415822_at	Scd2	stearoyl-Coenzyme A desaturase 2
1415835_at	Prl3b1	prolactin family 3, subfamily b, member 1
1415848_at	Prl3d1	prolactin family 3, subfamily d, member 1
1415899_at	Junb	Jun-B oncogene
1415906_at	Tmsb4x	thymosin, beta 4, X chromosome
1415920_at	Cstf2t	cleavage stimulation factor, 3' pre-RNA subunit 2, tau
1415938_at	Spink3	serine peptidase inhibitor, Kazal type 3
1415943_at	Sdc1	syndecan 1
1415983_at	Lcp1	lymphocyte cytosolic protein 1
1416066_at	Cd9	CD9 antigen
1416077_at	Adm	adrenomedullin
1416101_a_at	Hist1h1c	histone cluster 1, H1c
1416122_at	Ccnd2	cyclin D2
1416157_at	Vcl	vinculin
1416183_a_at	Ldhb	lactate dehydrogenase B
1416257_at	Capn2	calpain 2
1416268_at	Ets2	E26 avian leukemia oncogene 2, 3' domain
1416316_at	Slc27a2	solute carrier family 27 (fatty acid transporter), member 2
1416344_at	Lamp2	lysosomal-associated membrane protein 2
1416389_a_at	Rcbtb2	regulator of chromosome condensation (RCC1) and BTB (POZ) domain containing protein 2
1416405_at	Bgn	biglycan
1416413_at	Ctsj	cathepsin J
1416416_x_at	Gstm1	glutathione S-transferase, mu 1
1416455_a_at	Cryab	crystallin, alpha B
1416498_at	Ppic	peptidylprolyl isomerase C
1416514_a_at	Fscn1	fascin homolog 1, actin bundling protein (Strongylocentrotus purpuratus)
1416529_at	Emp1	epithelial membrane protein 1
1416576_at	Socs3	suppressor of cytokine signaling 3
1416589_at	Sparc	secreted acidic cysteine rich glycoprotein
1416614_at	Eid1	EP300 interacting inhibitor of differentiation 1
1416625_at	Serping1	serine (or cysteine) peptidase inhibitor, clade G, member 1
1416630_at	Id3	inhibitor of DNA binding 3
1416645_a_at	Afp	alpha fetoprotein
1416646_at	Afp	alpha fetoprotein
1416658_at	Frzb	frizzled-related protein
1416666_at	Serpine2	serine (or cysteine) peptidase inhibitor, clade E, member 2
1416673_at	Bace2	beta-site APP-cleaving enzyme 2

1416686_at	Plod2	procollagen lysine, 2-oxoglutarate 5-dioxygenase 2
1416700_at	Rnd3	Rho family GTPase 3
1416761_at	Hsd11b2	hydroxysteroid 11-beta dehydrogenase 2
1416808_at	Nid1	nidogen 1
1416953_at	Ctgf	connective tissue growth factor
1417061_at	Slc40a1	solute carrier family 40 (iron-regulated transporter), member 1
1417065_at	Egr1	early growth response 1
1417092_at	Pthr1	parathyroid hormone receptor 1
1417109_at	Tinagl1	tubulointerstitial nephritis antigen-like 1
1417110_at	Man1a	mannosidase 1, alpha
1417111_at	Man1a	mannosidase 1, alpha
1417133_at	Pmp22	peripheral myelin protein 22
1417145_at	Nfxl1	nuclear transcription factor, X-box binding-like 1
1417149_at	P4ha2	procollagen-proline, 2-oxoglutarate 4-dioxygenase (proline 4-hydroxylase), alpha II polypeptide
1417156_at	Krt19	keratin 19
1417162_at	Tmbim1	transmembrane BAX inhibitor motif containing 1
1417175_at	Csnk1e	casein kinase 1, epsilon
1417210_at	Eif2s3y	eukaryotic translation initiation factor 2, subunit 3, structural gene Y-linked
1417225_at	Arl6ip5	ADP-ribosylation factor-like 6 interacting protein 5
1417272_at	9130005N14Rik	RIKEN cDNA 9130005N14 gene
1417273_at	Pdk4	pyruvate dehydrogenase kinase, isoenzyme 4
1417355_at	Peg3	paternally expressed 3
1417356_at	Peg3	paternally expressed 3
1417392_a_at	Slc7a7	solute carrier family 7 (cationic amino acid transporter, y+ system), member 7
1417394_at	Klf4	Kruppel-like factor 4 (gut)
1417395_at	Klf4	Kruppel-like factor 4 (gut)
1417408_at	F3	coagulation factor III
1417490_at	Ctsb	cathepsin B
1417500_a_at	Tgm2	transglutaminase 2, C polypeptide
1417514_at	Ssx2ip	synovial sarcoma, X breakpoint 2 interacting protein
1417553_at	Plac1	placental specific protein 1
1417566_at	Abhd5	abhydrolase domain containing 5
1417649_at	Cdkn1c	cyclin-dependent kinase inhibitor 1C (P57)
1417738_at	Rab25	RAB25, member RAS oncogene family
1417741_at	Pygl	liver glycogen phosphorylase
1417760_at	Nr0b1	nuclear receptor subfamily 0, group B, member 1
1417837_at	Phlda2	pleckstrin homology-like domain, family A, member 2
1417850_at	Rb1	retinoblastoma 1

1417872_at	Fhl1	four and a half LIM domains 1
1417890_at	Pdxp	pyridoxal (pyridoxine, vitamin B6) phosphatase
1417896_at	Tjp3	tight junction protein 3
1417930_at	Nab2	Ngfi-A binding protein 2
1417962_s_at	Ghr	growth hormone receptor
1417976_at	Ada	adenosine deaminase
1417987_at	Btd	biotinidase
1418015_at	Pum2	pumilio 2 (Drosophila)
1418072_at	Hist1h2bb /// Hist1h2bc /// Hist1h2be /// Hist1h2bg /// LOC665622 /// RP23-38E20.1	histone cluster 1, H2bb /// histone cluster 1, H2bc /// histone cluster 1, H2be /// histone cluster 1, H2bg /// H2b histone family member /// predicted gene, OTTMUSG00000013203
1418084_at	Nrp1	neuropilin 1
1418094_s_at	Car4	carbonic anhydrase 4
1418133_at	Bcl3	B-cell leukemia/lymphoma 3
1418144_a_at	Pip5k1a	phosphatidylinositol-4-phosphate 5-kinase, type 1 alpha
1418153_at	Lama1	laminin, alpha 1
1418191_at	LOC100048346 /// Usp18	similar to ubiquitin specific protease UBP43 /// ubiquitin specific peptidase 18
1418228_at	Nfu1	NFU1 iron-sulfur cluster scaffold homolog (S. cerevisiae)
1418258_s_at	Dynll2	dynein light chain LC8-type 2
1418349_at	Hbegf	heparin-binding EGF-like growth factor
1418350_at	Hbegf	heparin-binding EGF-like growth factor
1418374_at	Fxyd3	FXYD domain-containing ion transport regulator 3
1418387_at	Mphosph8	M-phase phosphoprotein 8
1418422_at	Serpinb9g	serine (or cysteine) peptidase inhibitor, clade B, member 9g
1418423_s_at	OTTMUSG00000000724 /// Serpinb9e /// Serpinb9f /// Serpinb9g	predicted gene, OTTMUSG00000000724 /// serine (or cysteine) peptidase inhibitor, clade B, member 9e /// serine (or cysteine) peptidase inhibitor, clade B, member 9f /// serine (or cysteine) peptidase inhibitor, clade B, member 9g
1418486_at	Vnn1	vanin 1
1418501_a_at	Oxr1	oxidation resistance 1
1418538_at	Kdelr3	KDEL (Lys-Asp-Glu-Leu) endoplasmic reticulum protein retention receptor 3
1418569_at	Fblim1	filamin binding LIM protein 1
1418626_a_at	Clu	clusterin
1418648_at	Egln3	EGL nine homolog 3 (C. elegans)
1418649_at	Egln3	EGL nine homolog 3 (C. elegans)
1418723_at	Lpar3	lysophosphatidic acid receptor 3
1418744_s_at	LOC100047138 /// Tesc	similar to Tescalcin /// tescalcin
1418805_at	Sct	secretin
1418817_at	Chmp1b	chromatin modifying protein 1B

1418835_at	Phlda1	pleckstrin homology-like domain, family A, member 1
1418847_at	Arg2	arginase type II
1418918_at	Igfbp1	insulin-like growth factor binding protein 1
1419018_at	Rhox6	reproductive homeobox 6
1419091_a_at	Anxa2	annexin A2
1419103_a_at	Abhd6	abhydrolase domain containing 6
1419106_at	2210409E12Rik	RIKEN cDNA 2210409E12 gene
1419142_at	Ctsr	cathepsin R
1419149_at	Serpine1	serine (or cysteine) peptidase inhibitor, clade E, member 1
1419380_at	Zfp423	zinc finger protein 423
1419418_a_at	Morc1	microrchidia 1
1419540_at	EG434726 /// Fthl17 /// OTTMUSG00000016694 /// OTTMUSG00000016862 ///	predicted gene, EG434726 /// ferritin, heavy polypeptide-like 17 /// predicted gene, OTTMUSG00000016694 /// predicted gene, OTTMUSG00000016862 /// predicted gene, OTTMUSG00000016933
1419606_a_at	Tnnt1	troponin T1, skeletal, slow
1419641_at	Purb	purine rich element binding protein B
1419685_at	Upf1	UPF1 regulator of nonsense transcripts homolog (yeast)
1419838_s_at	Plk4	polo-like kinase 4 (Drosophila)
1420337_at	Gbx2	gastrulation brain homeobox 2
1420342_at	Gdap10	ganglioside-induced differentiation-associated-protein 10
1420425_at	Prdm1	PR domain containing 1, with ZNF domain
1420502_at	Sat1	spermidine/spermine N1-acetyl transferase 1
1420621_a_at	App	amyloid beta (A4) precursor protein
1420647_a_at	Krt8	keratin 8
1420664_s_at	Procr	protein C receptor, endothelial
1420715_a_at	Pparg	peroxisome proliferator activated receptor gamma
1420760_s_at	Ndrg1	N-myc downstream regulated gene 1
1420773_at	Dub1	deubiquitinating enzyme 1
1420903_at	St6galnac3	ST6 (alpha-N-acetyl-neuraminyl-2,3-beta-galactosyl-1,3)-N-acetylgalactosaminide alpha-2,6-sialyltransferase 3
1420911_a_at	Mfge8	milk fat globule-EGF factor 8 protein
1420913_at	Slco2a1	solute carrier organic anion transporter family, member 2a1
1420991_at	Ankrd1	ankyrin repeat domain 1 (cardiac muscle)
1421217_a_at	Lgals9	lectin, galactose binding, soluble 9
1421267_a_at	Cited2	Cbp/p300-interacting transactivator, with Glu/Asp-rich carboxy-terminal domain, 2
1421317_x_at	Myb	myeloblastosis oncogene
1421365_at	Fst	follistatin
1421375_a_at	S100a6	S100 calcium binding protein A6 (calcyclin)
1421529_a_at	Txnrd1	thioredoxin reductase 1

1421604_a_at	Klf3 /// LOC100046855	Kruppel-like factor 3 (basic) /// similar to BKLF
1421654_a_at	Lmna	lamin A
1421657_a_at	Sox17	SRY-box containing gene 17
1421817_at	Gsr	glutathione reductase
1421830_at	Ak3l1 /// LOC100047616 /// OTTMUSG0000001317	adenylate kinase 3-like 1 /// similar to adenylate kinase 4 /// predicted gene, OTTMUSG00000013175
1421839_at	Abca1	ATP-binding cassette, sub-family A (ABC1), member 1
1421882_a_at	Elavl2	ELAV (embryonic lethal, abnormal vision, Drosophila)-like 2 (Hu antigen B)
1421914_s_at	Mrpl19	mitochondrial ribosomal protein L19
1421952_at	Capn6	calpain 6
1422079_at	Prkch	protein kinase C, eta
1422289_a_at	Ctsq	cathepsin Q
1422476_at	Ifi30	interferon gamma inducible protein 30
1422510_at	Ctdspl	CTD (carboxy-terminal domain, RNA polymerase II, polypeptide A) small phosphatase-like
1422526_at	Acsl1	acyl-CoA synthetase long-chain family member 1
1422528_a_at	Zfp36l1	zinc finger protein 36, C3H type-like 1
1422566_at	Tcfeb	transcription factor EB
1422571_at	Thbs2	thrombospondin 2
1422836_at	Mbnl3	muscleblind-like 3 (Drosophila)
1422851_at	Hmga2	high mobility group AT-hook 2
1422912_at	Bmp4	bone morphogenetic protein 4
1422943_a_at	Hspb1	heat shock protein 1
1422962_a_at	Psmb8	proteasome (prosome, macropain) subunit, beta type 8 (large multifunctional peptidase 7)
1423103_at	Rfx5	regulatory factor X, 5 (influences HLA class II expression)
1423110_at	Col1a2	collagen, type I, alpha 2
1423187_at	Gabarapl2	gamma-aminobutyric acid (GABA-A) receptor-associated protein-like 2
1423192_at	Pspc1	paraspeckle protein 1
1423211_at	Nola3	nucleolar protein family A, member 3
1423280_at	Stmn2	stathmin-like 2
1423281_at	Stmn2	stathmin-like 2
1423327_at	Rpl39l	ribosomal protein L39-like
1423331_a_at	LOC100047693 /// Pvrl3	similar to cell adhesion molecule nectin-3 beta /// poliovirus receptor-related 3
1423361_at	Yme1l1	YME1-like 1 (S. cerevisiae)
1423379_at	Nfatc4	nuclear factor of activated T-cells, cytoplasmic, calcineurin-dependent 4
1423404_at	Gkn1	gastrokine 1
1423413_at	Ndrg1	N-myc downstream regulated gene 1
1423429_at	Rhox5	reproductive homeobox 5

1423450_a_at	Hs3st1	heparan sulfate (glucosamine) 3-O-sulfotransferase 1
1423452_at	Stk17b	serine/threonine kinase 17b (apoptosis-inducing)
1423470_at	Ptbp2	polypyrimidine tract binding protein 2
1423481_at	Riok2	RIO kinase 2 (yeast)
1423506_a_at	Nnat	neuronatin
1423523_at	Aass	aminoadipate-semialdehyde synthase
1423526_at	Arid3b	AT rich interactive domain 3B (BRIGHT-like)
1423537_at	Gap43	growth associated protein 43
1423606_at	Postn	periostin, osteoblast specific factor
1423669_at	Col1a1	collagen, type I, alpha 1
1423686_a_at	Prr13	proline rich 13
1423691_x_at	Krt8	keratin 8
1423725_at	Pls3	plastin 3 (T-isoform)
1423747_a_at	Pdk1	pyruvate dehydrogenase kinase, isoenzyme 1
1423748_at	Pdk1	pyruvate dehydrogenase kinase, isoenzyme 1
1423785_at	Egln1	EGL nine homolog 1 (C. elegans)
1423786_at	8430410A17Rik	RIKEN cDNA 8430410A17 gene
1423885_at	Lamc1	laminin, gamma 1
1423886_at	Lamc1	laminin, gamma 1
1423933_a_at	1600029D21Rik	RIKEN cDNA 1600029D21 gene
1423952_a_at	Krt7	keratin 7
1423986_a_at	Shisa5	shisa homolog 5 (Xenopus laevis)
1424046_at	Bub1	budding uninhibited by benzimidazoles 1 homolog (S. cerevisiae)
1424051_at	Col4a2	collagen, type IV, alpha 2
1424086_at	Oaf	OAF homolog (Drosophila)
1424113_at	Lamb1-1	laminin B1 subunit 1
1424114_s_at	Lamb1-1	laminin B1 subunit 1
1424123_at	Flvcr2	feline leukemia virus subgroup C cellular receptor family, member 2
1424214_at	9130213B05Rik	RIKEN cDNA 9130213B05 gene
1424263_at	2810003C17Rik	RIKEN cDNA 2810003C17 gene
1424295_at	Dppa3	developmental pluripotency-associated 3
1424296_at	Gclc	glutamate-cysteine ligase, catalytic subunit
1424343_a_at	Eif1a	eukaryotic translation initiation factor 1A
1424478_at	Bbs2	Bardet-Biedl syndrome 2 (human)
1424571_at	Ddx46	DEAD (Asp-Glu-Ala-Asp) box polypeptide 46
1424649_a_at	Tspan8	tetraspanin 8
1424719_a_at	Mapt	microtubule-associated protein tau
1424769_s_at	Cald1	caldesmon 1
1424775_at	Oas1a	2'-5' oligoadenylate synthetase 1A
1424927_at	Glipr1	GLI pathogenesis-related 1 (glioma)
1425106_a_at	Wars	tryptophanyl-tRNA synthetase
1425404_a_at	Tmem110	transmembrane protein 110
1425427_at	LOC639910	hypothetical protein LOC639910
1425458_a_at	Grb10	growth factor receptor bound protein 10

1425464_at	Gata6	GATA binding protein 6
1425536_at	Stx3	syntaxin 3
1425545_x_at	H2-D1	histocompatibility 2, D region locus 1
1425546_a_at	Trf	transferrin
1425567_a_at	Anxa5	annexin A5
1425767_a_at	Six4	sine oculis-related homeobox 4 homolog (Drosophila)
1425895_a_at	Id1	inhibitor of DNA binding 1
1425926_a_at	Otx2	orthodenticle homolog 2 (Drosophila)
1425964_x_at	Hspb1	heat shock protein 1
1426004_a_at	Tgm2	transglutaminase 2, C polypeptide
1426088_at	---	---
1426151_a_at	Stx3	syntaxin 3
1426208_x_at	Plagl1	pleiomorphic adenoma gene-like 1
1426221_at	Vwa5a	von Willebrand factor A domain containing 5A
1426225_at	Rbp4	retinol binding protein 4, plasma
1426235_a_at	Glul	glutamate-ammonia ligase (glutamine synthetase)
1426243_at	Cth	cystathionase (cystathionine gamma-lyase)
1426246_at	Pros1	protein S (alpha)
1426348_at	Col4a1	collagen, type IV, alpha 1
1426370_at	Far1	fatty acyl CoA reductase 1
1426371_at	Far1	fatty acyl CoA reductase 1
1426397_at	Tgfbr2	transforming growth factor, beta receptor II
1426454_at	Arhgdib	Rho, GDP dissociation inhibitor (GDI) beta
1426587_a_at	Stat3	signal transducer and activator of transcription 3
1426808_at	Lgals3	lectin, galactose binding, soluble 3
1426812_a_at	9130404D14Rik	RIKEN cDNA 9130404D14 gene
1426840_at	Ythdf3	YTH domain family 3
1426858_at	Inhbb ///	inhibin beta-B /// similar to Inhbb protein
1426951_at	Crim1	cysteine rich transmembrane BMP regulator 1 (chordin like)
1426980_s_at	E130012A19Rik	RIKEN cDNA E130012A19 gene
1426990_at	Cubn	cubilin (intrinsic factor-cobalamin receptor)
1427108_at	9530068E07Rik	RIKEN cDNA 9530068E07 gene
1427126_at	Hspa1b	heat shock protein 1B
1427127_x_at	Hspa1b	heat shock protein 1B
1427133_s_at	Lrp2	low density lipoprotein receptor-related protein 2
1427202_at	4833442J19Rik	RIKEN cDNA 4833442J19 gene
1427238_at	Fbxo15	F-box protein 15
1427302_at	Enpp3	ectonucleotide pyrophosphatase/phosphodiesterase 3
1427442_a_at	App	amyloid beta (A4) precursor protein

1427479_at	100039042 /// 100039129 /// 100042776 /// 627881 /// BB287469 /// EG266459 /// EG435337 /// EG544883 /// EG666862 /// Eif1a /// LOC100039226 /// LOC641136	predicted gene, 100039042 /// predicted gene, 100039129 /// predicted gene, 100042776 /// predicted gene, 627881 /// expressed sequence BB287469 /// predicted gene, EG266459 /// predicted gene, EG435337 /// predicted gene, EG544883 /// predicted gene, EG666862 /// eukaryotic translation initiation factor 1A /// hypothetical protein LOC100039226 /// similar to X-linked eukaryotic translation initiation factor 1A
1427746_x_at	H2-K1	histocompatibility 2, K1, K region
1427760_s_at	Prl2c2 /// Prl2c3 /// Prl2c4	prolactin family 2, subfamily c, member 2 /// prolactin family 2, subfamily c, member 3 /// prolactin family 2, subfamily c, member 4
1427762_x_at	Hist1h2bp	histone cluster 1, H2bp
1427764_a_at	Tcfe2a	transcription factor E2a
1427768_s_at	Myl3	myosin, light polypeptide 3
1427883_a_at	Col3a1	collagen, type III, alpha 1
1427960_at	Ugt2b34	UDP glucuronosyltransferase 2 family, polypeptide B34
1427961_s_at	Ugt2b34	UDP glucuronosyltransferase 2 family, polypeptide B34
1428079_at	Fgb	fibrinogen, B beta polypeptide
1428103_at	Adam10	a disintegrin and metallopeptidase domain 10
1428111_at	Slc38a4	solute carrier family 38, member 4
1428125_at	ENSMUSG0000007474	predicted gene, ENSMUSG00000074747
1428146_s_at	Acaa2	acetyl-Coenzyme A acyltransferase 2 (mitochondrial 3-oxoacyl-Coenzyme A thiolase)
1428282_at	Tbce	tubulin-specific chaperone e
1428301_at	100041195 /// 100041874 /// 666442 /// 666637 /// ENSMUSG00000063277 /// ENSMUSG00000068790 /// ENSMUSG00000072735 /// LOC100036568 ///	predicted gene, 100041195 /// predicted gene, 100041874 /// predicted gene, 666442 /// predicted gene, 666637 /// predicted gene, ENSMUSG00000063277 /// predicted gene, ENSMUSG00000068790 /// predicted gene, ENSMUSG00000072735 /// hypothetical LOC100036568 /// hypothetical protein LOC544988 /// hypothetical protein LOC671957
1428306_at	Ddit4	DNA-damage-inducible transcript 4
1428853_at	Ptch1	patched homolog 1
1429177_x_at	Sox17	SRY-box containing gene 17
1429252_at	0610010K14Rik	RIKEN cDNA 0610010K14 gene
1429483_at	Calcoco2	calcium binding and coiled-coil domain 2
1429530_a_at	Smpd4	sphingomyelin phosphodiesterase 4
1430125_s_at	Pqlc1	PQ loop repeat containing 1
1430127_a_at	Ccnd2	cyclin D2
1431292_a_at	Twf2	twinfilin, actin-binding protein, homolog 2 (Drosophila)

1431416_a_at	Jam2	junction adhesion molecule 2
1431701_a_at	Pdzk1	PDZ domain containing 1
1431805_a_at	Rhpn2	rhophilin, Rho GTPase binding protein 2
1433428_x_at	Tgm2	transglutaminase 2, C polypeptide
1433442_at	Klhl9	kelch-like 9 (Drosophila)
1433471_at	Tcf7	transcription factor 7, T-cell specific
1433488_x_at	Gns	glucosamine (N-acetyl)-6-sulfatase
1433514_at	Etnk1	ethanolamine kinase 1
1433531_at	Acsl4	acyl-CoA synthetase long-chain family member 4
1433830_at	Hnrnpa2b1	heterogeneous nuclear ribonucleoprotein A2/B1
1433956_at	Cdh5	cadherin 5
1434025_at	---	---
1434369_a_at	Cryab	crystallin, alpha B
1434499_a_at	Ldhb	lactate dehydrogenase B
1434528_at	Aard	alanine and arginine rich domain containing protein
1434628_a_at	Rhpn2	rhophilin, Rho GTPase binding protein 2
1434745_at	Ccnd2	cyclin D2
1434897_a_at	Slc25a4	solute carrier family 25 (mitochondrial carrier, adenine nucleotide translocator), member 4
1435086_s_at	Klhdc2	kelch domain containing 2
1435176_a_at	Id2	inhibitor of DNA binding 2
1435494_s_at	Dsp	desmoplakin
1435526_at	Tor1aip2	torsin A interacting protein 2
1435530_at	Camsap1	calmodulin regulated spectrin-associated protein 1
1435561_at	Erf	Ets2 repressor factor
1435989_x_at	Krt8	keratin 8
1436291_a_at	Dpys	dihydropyrimidinase
1436399_s_at	Nrk	Nik related kinase
1436419_a_at	1700097N02Rik	RIKEN cDNA 1700097N02 gene
1436584_at	Spry2	sprouty homolog 2 (Drosophila)
1436714_at	Lpp	LIM domain containing preferred translocation partner in lipoma
1436879_x_at	Afp	alpha fetoprotein
1436959_x_at	Nelf	nasal embryonic LHRH factor
1436991_x_at	Gsn	gelsolin
1437100_x_at	Pim3	proviral integration site 3
1437165_a_at	Pcolce	procollagen C-endopeptidase enhancer protein
1437171_x_at	Gsn	gelsolin
1437277_x_at	Tgm2	transglutaminase 2, C polypeptide
1437279_x_at	Sdc1	syndecan 1
1437308_s_at	F2r	coagulation factor II (thrombin) receptor
1437340_x_at	Gkn1	gastrokine 1
1437458_x_at	Clu	clusterin
1437689_x_at	Clu	clusterin

1437810_a_at	Hbb-bh1 /// LOC100044263	hemoglobin Z, beta-like embryonic chain /// hypothetical protein LOC100044263
1437990_x_at	Hbb-bh1 /// LOC100044263	hemoglobin Z, beta-like embryonic chain /// hypothetical protein LOC100044263
1438118_x_at	Vim	vimentin
1438855_x_at	Tnfaip2	tumor necrosis factor, alpha-induced protein 2
1438941_x_at	Ampd2	adenosine monophosphate deaminase 2 (isoform L)
1439002_s_at	Prl3d1	prolactin family 3, subfamily d, member 1
1439415_x_at	EG546663 /// Rps21	predicted gene, EG546663 /// ribosomal protein S21
1439440_x_at	Twf2	twinfilin, actin-binding protein, homolog 2 (Drosophila)
1439476_at	Dsg2	desmoglein 2
1440831_at	Bach1	BTB and CNC homology 1
1443892_at	EG435970 /// LOC100045488	predicted gene, EG435970 /// similar to crooked legs CG14938-PB
1447820_x_at	Cpt2	carnitine palmitoyltransferase 2
1448029_at	Tbx3	T-box 3
1448121_at	Wbp2	WW domain binding protein 2
1448123_s_at	Tgfbi	transforming growth factor, beta induced
1448152_at	Igf2	insulin-like growth factor 2
1448194_a_at	H19	H19 fetal liver mRNA
1448201_at	Sfrp2	secreted frizzled-related protein 2
1448203_at	Atp5l /// ENSMUSG0000006771 9 /// OTTMUSG0000000277	ATP synthase, H+ transporting, mitochondrial F0 complex, subunit g /// predicted gene, ENSMUSG00000067719 /// predicted gene, OTTMUSG00000002778
1448213_at	Anxa1	annexin A1
1448229_s_at	Ccnd2	cyclin D2
1448237_x_at	Ldhb	lactate dehydrogenase B
1448239_at	Hmox1	heme oxygenase (decycling) 1
1448260_at	Uchl1	ubiquitin carboxy-terminal hydrolase L1
1448315_a_at	Pycr2	pyrroline-5-carboxylate reductase family, member 2
1448330_at	Gstm1	glutathione S-transferase, mu 1
1448380_at	Lgals3bp	lectin, galactoside-binding, soluble, 3 binding protein
1448392_at	Sparc	secreted acidic cysteine rich glycoprotein
1448393_at	Cldn7	claudin 7
1448424_at	Frzb	frizzled-related protein
1448469_at	Nid1	nidogen 1
1448491_at	Ech1	enoyl coenzyme A hydratase 1, peroxisomal
1448499_a_at	Ephx2	epoxide hydrolase 2, cytoplasmic
1448562_at	Upp1	uridine phosphorylase 1
1448566_at	Slc40a1	solute carrier family 40 (iron-regulated transporter), member 1
1448572_at	Prl4a1	prolactin family 4, subfamily a, member 1
1448592_at	Crtap	cartilage associated protein

1448653_at	Eed	embryonic ectoderm development
1448729_a_at	4-Sep	septin 4
1448732_at	Ctsb	cathepsin B
1448742_at	Snai1	snail homolog 1 (Drosophila)
1448743_at	Ssx2ip	synovial sarcoma, X breakpoint 2 interacting protein
1448845_at	Rpp25	ribonuclease P 25 subunit (human)
1448889_at	Slc38a4	solute carrier family 38, member 4
1448904_at	D6Wsu176e	DNA segment, Chr 6, Wayne State University 176, expressed
1448949_at	Car4	carbonic anhydrase 4
1448964_at	S100g	S100 calcium binding protein G
1449032_at	Prl2a1	prolactin family 2, subfamily a, member 1
1449078_at	St3gal6	ST3 beta-galactoside alpha-2,3-sialyltransferase 6
1449090_a_at	Yes1	Yamaguchi sarcoma viral (v-yes) oncogene homolog 1
1449109_at	Socs2	suppressor of cytokine signaling 2
1449119_at	Arih2	ariadne homolog 2 (Drosophila)
1449141_at	Fblim1	filamin binding LIM protein 1
1449171_at	Ttk	Ttk protein kinase
1449204_at	Gjb5	gap junction protein, beta 5
1449253_at	Smc1b	structural maintenance of chromosomes 1B
1449254_at	Spp1	secreted phosphoprotein 1
1449289_a_at	B2m	beta-2 microglobulin
1449347_a_at	LOC100044048 /// LOC100044049 /// Xlr4a /// Xlr4b /// Xlr4c /// Xlr4e	hypothetical protein LOC100044048 /// hypothetical protein LOC100044049 /// X-linked lymphocyte-regulated 4A /// X-linked lymphocyte-regulated 4B /// X-linked lymphocyte-regulated 4C /// X-linked lymphocyte-regulated 4E
1449357_at	2310030G06Rik	RIKEN cDNA 2310030G06 gene
1449408_at	Jam2	junction adhesion molecule 2
1449502_at	Dazl	deleted in azoospermia-like
1449529_s_at	Prl7a1	prolactin family 7, subfamily a, member 1
1449534_at	Sycp3	synaptonemal complex protein 3
1449540_at	Rhox9	reproductive homeobox 9
1449559_at	LOC100046255 /// Msx2	similar to homeobox protein /// homeobox, msh-like 2
1449590_a_at	Mras	muscle and microspikes RAS
1449939_s_at	Dlk1	delta-like 1 homolog (Drosophila)
1449968_s_at	Acot10 /// Acot9	acyl-CoA thioesterase 10 /// acyl-CoA thioesterase 9
1450032_at	Slco2a1	solute carrier organic anion transporter family, member 2a1
1450034_at	Stat1	signal transducer and activator of transcription 1
1450053_at	Kif2a	Kinesin family member 2A
1450078_at	Nrk	Nik related kinase
1450079_at	Nrk	Nik related kinase
1450333_a_at	Gata2	GATA binding protein 2

1450429_at	Capn6	calpain 6
1450461_at	Tcf7	transcription factor 7, T-cell specific
1450641_at	Vim	vimentin
1450644_at	Zfp36l1	zinc finger protein 36, C3H type-like 1
1450731_s_at	Tnfrsf21	tumor necrosis factor receptor superfamily, member 21
1450780_s_at	Hmga2	high mobility group AT-hook 2
1450781_at	Hmga2	high mobility group AT-hook 2
1450843_a_at	Serpinh1	serine (or cysteine) peptidase inhibitor, clade H, member 1
1450852_s_at	F2r	coagulation factor II (thrombin) receptor
1450857_a_at	Col1a2	collagen, type I, alpha 2
1450878_at	Sri	sorcin
1450894_a_at	Ap2m1	adaptor protein complex AP-2, mu1
1450947_at	2610528J11Rik	RIKEN cDNA 2610528J11 gene
1450989_at	Tdgf1	teratocarcinoma-derived growth factor 1
1450992_a_at	Meis1	Meis homeobox 1
1450997_at	Stk17b	serine/threonine kinase 17b (apoptosis-inducing)
1451021_a_at	Klf5	Kruppel-like factor 5
1451058_at	Mcts2	malignant T cell amplified sequence 2
1451069_at	Pim3	proviral integration site 3
1451241_at	Lamb1-1	laminin B1 subunit 1
1451264_at	Frmd6	FERM domain containing 6
1451320_at	Arhgap8	Rho GTPase activating protein 8
1451416_a_at	Tgm1	transglutaminase 1, K polypeptide
1451458_at	Tmem2	transmembrane protein 2
1451526_at	Arhgap12	Rho GTPase activating protein 12
1451683_x_at	H2-D1	histocompatibility 2, D region locus 1
1451784_x_at	H2-D1	histocompatibility 2, D region locus 1
1451790_a_at	Tfpi	tissue factor pathway inhibitor
1451791_at	Tfpi	tissue factor pathway inhibitor
1451839_a_at	Pde7a	phosphodiesterase 7A
1451931_x_at	H2-L	histocompatibility 2, D region
1452035_at	Col4a1	collagen, type IV, alpha 1
1452114_s_at	Igfbp5	insulin-like growth factor binding protein 5
1452165_at	Prl2b1	prolactin family 2, subfamily b, member 1
1452181_at	Ckap4	cytoskeleton-associated protein 4
1452192_at	Naf1	nuclear assembly factor 1 homolog (S. cerevisiae)
1452207_at	Cited2	Cbp/p300-interacting transactivator, with Glu/Asp-rich carboxy-terminal domain, 2
1452214_at	Skil	SKI-like
1452217_at	Ahnak	AHNAK nucleoprotein (desmoyokin)
1452270_s_at	Cubn	cubilin (intrinsic factor-cobalamin receptor)
1452286_at	Slain2	SLAIN motif family, member 2
1452318_a_at	Hspa1b	heat shock protein 1B
1452320_at	Lrp2	low density lipoprotein receptor-related protein 2
1452368_at	Bcr	breakpoint cluster region

1452384_at	Enpp3	ectonucleotide pyrophosphatase/phosphodiesterase 3
1452432_at	Tfpi	tissue factor pathway inhibitor
1452514_a_at	Kit	kit oncogene
1452540_a_at	Hist1h2bb /// Hist1h2bc /// Hist1h2be /// Hist1h2bf /// Hist1h2bg /// Hist1h2bj /// Hist1h2bl /// Hist1h2bm /// Hist1h2bn /// Hist1h2bp /// LOC100046213 /// LOC665622 /// RP23-38E20.1	histone cluster 1, H2bb /// histone cluster 1, H2bc /// histone cluster 1, H2be /// histone cluster 1, H2bf /// histone cluster 1, H2bg /// histone cluster 1, H2bj /// histone cluster 1, H2bl /// histone cluster 1, H2bm /// histone cluster 1, H2bn /// histone cluster 1, H2bp /// similar to Hist1h2bj protein /// H2b histone family member /// predicted gene, OTTMUSG00000013203
1452670_at	Myl9	myosin, light polypeptide 9, regulatory
1452731_x_at	100040880 /// 100041195 /// 100041874 /// 100042164 /// 666442 /// 666637 /// B930046C15Rik /// ENSMUSG0000006327 7 /// ENSMUSG0000006879 0 /// ENSMUSG0000007273	predicted gene, 100040880 /// predicted gene, 100041195 /// predicted gene, 100041874 /// predicted gene, 100042164 /// predicted gene, 666442 /// predicted gene, 666637 /// RIKEN cDNA B930046C15 gene /// predicted gene, ENSMUSG00000063277 /// predicted gene, ENSMUSG00000068790 /// predicted gene, ENSMUSG00000072735 /// hypothetical LOC100036568 /// similar to 1700001E04Rik protein /// hypothetical protein LOC671957
1453063_at	Cltb	clathrin, light polypeptide (Lcb)
1453132_a_at	Gkn2	gastrokine 2
1453836_a_at	Mgll	monoglyceride lipase
1453988_a_at	Ide	insulin degrading enzyme
1454045_a_at	Pgs1	phosphatidylglycerophosphate synthase 1
1454046_x_at	Pgs1	phosphatidylglycerophosphate synthase 1
1454677_at	Timp2	tissue inhibitor of metalloproteinase 2
1454681_at	Rbm35a	RNA binding motif protein 35A
1454760_at	Htatsf1	HIV TAT specific factor 1
1454794_at	Spast	spastin
1454849_x_at	Clu	clusterin
1455056_at	Lmo7	LIM domain only 7
1455061_a_at	Acaa2	acetyl-Coenzyme A acyltransferase 2 (mitochondrial 3-oxoacyl-Coenzyme A thiolase)
1455195_at	EG383815 /// EG547267 /// EG668041 /// LOC677113 /// Rps24	predicted gene, EG383815 /// predicted gene, EG547267 /// predicted gene, EG668041 /// similar to ribosomal protein S24 /// ribosomal protein S24
1455214_at	Mitf	microphthalmia-associated transcription factor
1455229_x_at	Pgs1	phosphatidylglycerophosphate synthase 1
1455235_x_at	Ldhb	lactate dehydrogenase B
1455692_x_at	1700097N02Rik	RIKEN cDNA 1700097N02 gene
1455798_at	Galk2	galactokinase 2

1455871_s_at	OTTMUSG0000001289 3 /// Rpl13 /// Tax1bp3	predicted gene, OTTMUSG00000012893 /// ribosomal protein L13 /// Tax1 (human T-cell leukemia virus type I) binding protein 3
1455899_x_at	Socs3	suppressor of cytokine signaling 3
1455913_x_at	Ttr	transthyretin
1455956_x_at	Ccnd2	cyclin D2
1456174_x_at	Ndrg1	N-myc downstream regulated gene 1
1456212_x_at	Socs3	suppressor of cytokine signaling 3
1456292_a_at	Vim	vimentin
1456312_x_at	Gsn	gelsolin
1456315_a_at	Ptpla	protein tyrosine phosphatase-like (proline instead of catalytic arginine), member a
1456388_at	Atp11a	ATPase, class VI, type 11A
1456573_x_at	Nnt	nicotinamide nucleotide transhydrogenase
1456733_x_at	Serpinh1	serine (or cysteine) peptidase inhibitor, clade H, member 1
1460038_at	LOC100045707 /// Pou3f1	similar to long overlapping ORF; NH2 terminus uncertain /// POU domain, class 3, transcription factor 1
1460217_at	Prl7d1	prolactin family 7, subfamily d, member 1
1460319_at	Fut8	fucosyltransferase 8
1460330_at	Anxa3	annexin A3
1460386_a_at	Slc1a1	solute carrier family 1 (neuronal/epithelial high affinity glutamate transporter, system Xag), member 1
1460605_at	Crxos1	Crx opposite strand transcript 1
1460700_at	Stat3	signal transducer and activator of transcription 3
1460740_at	Cltb /// LOC100046457	clathrin, light polypeptide (Lcb) /// similar to Cltb protein
