# Supplementary material for: Knowledge-guided gene ranking by coordinative component analysis
Source: BMC Bioinformatics. 2010 Mar 30;11:162. doi: 10.1186/1471-2105-11-162 (PMC2865494; doi:10.1186/1471-2105-11-162)
Supplement: Additional file 4 — The top 500 probe sets ranked by TGFβ pathway-guided COCA approach. [file 1471-2105-11-162-S4.PDF]

| Probe Set ID | Gene Symbol | Gene Name |
|---|---|---|
| 1415737_at | Rfk | riboflavin kinase |
| 1415802_at | Slc16a1 | solute carrier family 16 (monocarboxylic acid |
| 1415812_at | Gsn | gelsolin |
| 1415834_at | Dusp6 | dual specificity phosphatase 6 |
| 1415835_at | Prl3b1 | prolactin family 3, subfamily b, member 1 |
| 1415837_at | Klk1 | kallikrein 1 |
| 1415856_at | Emb | embigin |
| 1415857_at | Emb | embigin |
| 1415927_at | Actc1 /// LOC100048431 | actin, alpha, cardiac muscle 1 /// similar to alpha-actin (AA 27-375) |
| 1415938_at | Spink3 | serine peptidase inhibitor, Kazal type 3 |
| 1415961_at | Itm2c | integral membrane protein 2C |
| 1416002_x_at | Cotl1 | coactosin-like 1 (Dictyostelium) |
| 1416034_at | Cd24a /// EG621324 | CD24a antigen /// predicted gene, EG621324 |
| 1416039_x_at | Cyr61 | cysteine rich protein 61 |
| 1416041_at | Sgk1 | serum/glucocorticoid regulated kinase 1 |
| 1416048_at | Phc2 | polyhomeotic-like 2 (Drosophila) |
| 1416069_at | Pfkp | phosphofructokinase, platelet |
| 1416129_at | Errfi1 | ERBB receptor feedback inhibitor 1 |
| 1416155_at | Hmgb3 | high mobility group box 3 |
| 1416156_at | Vcl | vinculin |
| 1416213_x_at | Surf4 | surfeit gene 4 |
| 1416220_at | Spcs1 | signal peptidase complex subunit 1 homolog (S. |
| 1416230_at | Rfk | riboflavin kinase |
| 1416257_at | Capn2 | calpain 2 |
| 1416308_at | Ugdh | UDP-glucose dehydrogenase |
| 1416389_a_at | Rcbtb2 | regulator of chromosome condensation (RCC1) and |
| 1416390_at | Rcbtb2 | regulator of chromosome condensation (RCC1) and |
| 1416413_at | Ctsj | cathepsin J |
| 1416418_at | Gabarapl1 | gamma-aminobutyric acid (GABA(A)) receptor- |
| 1416419_s_at | Gabarapl1 | gamma-aminobutyric acid (GABA(A)) receptor- |
| 1416454_s_at | Acta2 | actin, alpha 2, smooth muscle, aorta |
| 1416455_a_at | Cryab | crystallin, alpha B |
| 1416521_at | Sepw1 | selenoprotein W, muscle 1 |
| 1416617_at | Acss1 | acyl-CoA synthetase short-chain family member 1 |
| 1416626_at | Pla2g1b | phospholipase A2, group IB, pancreas |
| 1416630_at | Id3 | inhibitor of DNA binding 3 |
| 1416638_at | Sall2 | sal-like 2 (Drosophila) |
| 1416645_a_at | Afp | alpha fetoprotein |
| 1416646_at | Afp | alpha fetoprotein |
| 1416656_at | Clic1 | chloride intracellular channel 1 |
| 1416666_at | Serpine2 | serine (or cysteine) peptidase inhibitor, clade E, |
| 1416696_at | D17Wsu104e | DNA segment, Chr 17, Wayne State University 104, |
| 1416697_at | Dpp4 | dipeptidylpeptidase 4 |
| 1416700_at | Rnd3 | Rho family GTPase 3 |
| 1416701_at | Rnd3 | Rho family GTPase 3 |
| 1416762_at | S100a10 | S100 calcium binding protein A10 (calpactin) |
| 1416767_a_at | 1110003E01Rik | RIKEN cDNA 1110003E01 gene |
| 1416832_at | Slc39a8 | solute carrier family 39 (metal ion transporter), member |
| 1416838_at | Mut | methylmalonyl-Coenzyme A mutase |

| | | |
|---|---|---|
| 1416880_at | Mcl1 | myeloid cell leukemia sequence 1 |
| 1416953_at | Ctgf | connective tissue growth factor |
| 1416967_at | Sox2 | SRY-box containing gene 2 |
| 1417056_at | Psme1 | proteasome (prosome, macropain) 28 subunit, alpha |
| 1417061_at | Slc40a1 | solute carrier family 40 (iron-regulated transporter), |
| 1417079_s_at | Lgals2 | lectin, galactose-binding, soluble 2 |
| 1417080_a_at | Ecsit | ECSIT homolog (Drosophila) |
| 1417090_at | Rcn1 | reticulocalbin 1 |
| 1417092_at | Pthr1 | parathyroid hormone receptor 1 |
| 1417109_at | Tinagl1 | tubulointerstitial nephritis antigen-like 1 |
| 1417156_at | Krt19 | keratin 19 |
| 1417189_at | Psme2 | proteasome (prosome, macropain) 28 subunit, beta |
| 1417231_at | Cldn2 | claudin 2 |
| 1417279_at | Itpr1 | inositol 1,4,5-triphosphate receptor 1 |
| 1417346_at | Pycard | PYD and CARD domain containing |
| 1417355_at | Peg3 | paternally expressed 3 |
| 1417388_at | Bex2 | brain expressed X-linked 2 |
| 1417396_at | Podxl | podocalyxin-like |
| 1417398_at | Rras2 | related RAS viral (r-ras) oncogene homolog 2 |
| 1417403_at | Elovl6 | ELOVL family member 6, elongation of long chain fatty |
| 1417408_at | F3 | coagulation factor III |
| 1417409_at | Jun | Jun oncogene |
| 1417428_at | Gng3 | guanine nucleotide binding protein (G protein), gamma |
| 1417478_a_at | Ppp2r3c | protein phosphatase 2, regulatory subunit B'', gamma |
| 1417490_at | Ctsb | cathepsin B |
| 1417638_at | Lefty1 | left right determination factor 1 |
| 1417666_at | Dnttip1 | deoxynucleotidyltransferase, terminal, interacting |
| 1417760_at | Nr0b1 | nuclear receptor subfamily 0, group B, member 1 |
| 1417804_at | Rasgrp2 | RAS, guanyl releasing protein 2 |
| 1417815_a_at | Serinc3 | serine incorporator 3 |
| 1417837_at | Phlda2 | pleckstrin homology-like domain, family A, member 2 |
| 1417868_a_at | Ctsz | cathepsin Z |
| 1417879_at | Nenf | neuron derived neurotrophic factor |
| 1417920_at | Amn | amnionless |
| 1417950_a_at | Apoa2 | apolipoprotein A-II |
| 1417959_at | Pdlim7 | PDZ and LIM domain 7 |
| 1417963_at | Pltp | phospholipid transfer protein |
| 1418000_a_at | Itm2b | integral membrane protein 2B |
| 1418025_at | Bhlhb2 | basic helix-loop-helix domain containing, class B2 |
| 1418057_at | Tiam1 | T-cell lymphoma invasion and metastasis 1 |
| 1418069_at | Apoc2 | apolipoprotein C-II |
| 1418072_at | Hist1h2bb /// Hist1h2bc /// Hist1h2be /// Hist1h2bg /// LOC665622 /// RP23-38E20.1 | histone cluster 1, H2bb /// histone cluster 1, H2bc /// histone cluster 1, H2be /// histone cluster 1, H2bg /// H2b histone family member /// predicted gene, OTTMUSG00000013203 |
| 1418094_s_at | Car4 | carbonic anhydrase 4 |
| 1418097_a_at | Crlf2 | cytokine receptor-like factor 2 |
| 1418171_at | LOC100045031 /// Tceal8 | similar to transcription elongation factor A (SII)-like 8 /// transcription elongation factor A (SII)-like 8 |

| | | |
|---|---|---|
| 1418189_s_at | Malat1 | metastasis associated lung adenocarcinoma transcript |
| 1418209_a_at | Pfn2 | profilin 2 |
| 1418240_at | Gbp2 | guanylate binding protein 2 |
| 1418318_at | Rnf128 | ring finger protein 128 |
| 1418362_at | Zfp42 | zinc finger protein 42 |
| 1418365_at | Ctsh | cathepsin H |
| 1418370_at | Tnnc1 | troponin C, cardiac/slow skeletal |
| 1418436_at | Stx7 | syntaxin 7 |
| 1418501_a_at | Oxr1 | oxidation resistance 1 |
| 1418514_at | Mtf2 | metal response element binding transcription factor 2 |
| 1418515_at | Mtf2 | metal response element binding transcription factor 2 |
| 1418516_at | Mtf2 | metal response element binding transcription factor 2 |
| 1418532_at | Fzd2 | frizzled homolog 2 (Drosophila) |
| 1418533_s_at | Fzd2 | frizzled homolog 2 (Drosophila) |
| 1418565_at | Serbp1 | Serpine1 mRNA binding protein 1 |
| 1418648_at | Egln3 | EGL nine homolog 3 (C. elegans) |
| 1418649_at | Egln3 | EGL nine homolog 3 (C. elegans) |
| 1418703_at | Rbms1 | RNA binding motif, single stranded interacting protein 1 |
| 1418756_at | Trh | thyrotropin releasing hormone |
| 1418835_at | Phlda1 | pleckstrin homology-like domain, family A, member 1 |
| 1418884_x_at | Tuba1a | tubulin, alpha 1A |
| 1418916_a_at | Spp2 | secreted phosphoprotein 2 |
| 1419018_at | Rhox6 | reproductive homeobox 6 |
| 1419021_at | Mcf2 | mcf.2 transforming sequence |
| 1419029_at | Ero1l | ERO1-like (S. cerevisiae) |
| 1419062_at | Epb4.1l3 | erythrocyte protein band 4.1-like 3 |
| 1419086_at | Fgfbp1 | fibroblast growth factor binding protein 1 |
| 1419095_a_at | Apom | apolipoprotein M |
| 1419096_at | Apom | apolipoprotein M |
| 1419123_a_at | Pdgfc | platelet-derived growth factor, C polypeptide |
| 1419135_at | Ltb | lymphotoxin B |
| 1419154_at | Tmprss2 | transmembrane protease, serine 2 |
| 1419232_a_at | Apoa1 | apolipoprotein A-I |
| 1419233_x_at | Apoa1 | apolipoprotein A-I |
| 1419236_at | Helb | helicase (DNA) B |
| 1419351_a_at | l7Rn6 | lethal, Chr 7, Rinchik 6 |
| 1419352_at | l7Rn6 | lethal, Chr 7, Rinchik 6 |
| 1419399_at | Mttp | microsomal triglyceride transfer protein |
| 1419400_at | Mttp | microsomal triglyceride transfer protein |
| 1419456_at | Dcxr | dicarbonyl L-xylulose reductase |
| 1419606_a_at | Tnnt1 | troponin T1, skeletal, slow |
| 1419656_at | Slc25a36 | solute carrier family 25, member 36 |
| 1419657_a_at | Slc25a36 | solute carrier family 25, member 36 |
| 1419685_at | Upf1 | UPF1 regulator of nonsense transcripts homolog |
| 1419700_a_at | Prom1 | prominin 1 |
| 1419814_s_at | S100a1 | S100 calcium binding protein A1 |
| 1419874_x_at | Zbtb16 | zinc finger and BTB domain containing 16 |
| 1420085_at | Fgf4 | fibroblast growth factor 4 |
| 1420086_x_at | Fgf4 | fibroblast growth factor 4 |
| 1420376_a_at | H3f3a /// H3f3b /// LOC100045490 | H3 histone, family 3A /// H3 histone, family 3B /// similar to H3 histone, family 3A |

| | | |
|---|---|---|
| 1420388_at | Prss12 | protease, serine, 12 neurotrypsin (motopsin) |
| 1420441_at | Cenpc1 | centromere protein C1 |
| 1420498_a_at | Dab2 | disabled homolog 2 (Drosophila) |
| 1420549_at | Gbp1 | guanylate binding protein 1 |
| 1420612_s_at | Ptp4a2 | protein tyrosine phosphatase 4a2 |
| 1420621_a_at | App | amyloid beta (A4) precursor protein |
| 1420624_a_at | Vamp8 | vesicle-associated membrane protein 8 |
| 1420628_at | LOC100045958 /// Pura | similar to hCG45299 /// purine rich element binding protein A |
| 1420647_a_at | Krt8 | keratin 8 |
| 1420711_a_at | Pex2 | peroxisome biogenesis factor 2 |
| 1420827_a_at | Ccng1 | cyclin G1 |
| 1420867_at | ENSMUSG000000744 60 /// Tmed2 | predicted gene, ENSMUSG00000074460 /// transmembrane emp24 domain trafficking protein 2 |
| 1420918_at | Sgk3 | serum/glucocorticoid regulated kinase 3 |
| 1420930_s_at | Ctnnal1 | catenin (cadherin associated protein), alpha-like 1 |
| 1420998_at | Etv5 | ets variant gene 5 |
| 1421267_a_at | Cited2 | Cbp/p300-interacting transactivator, with Glu/Asp-rich |
| 1421365_at | Fst | follistatin |
| 1421375_a_at | S100a6 | S100 calcium binding protein A6 (calcyclin) |
| 1421491_a_at | Tmem49 | transmembrane protein 49 |
| 1421708_a_at | Stat6 | signal transducer and activator of transcription 6 |
| 1421811_at | LOC640441 /// Thbs1 | similar to thrombospondin 1 /// thrombospondin 1 |
| 1421812_at | Tapbp | TAP binding protein |
| 1421851_at | Mtap1b | microtubule-associated protein 1B |
| 1421852_at | Kcnk5 | potassium channel, subfamily K, member 5 |
| 1421871_at | Sh3bgrl | SH3-binding domain glutamic acid-rich protein like |
| 1422142_at | Nphs1 | nephrosis 1 homolog, nephrin (human) |
| 1422264_s_at | 2310051E17Rik /// Klf9 | RIKEN cDNA 2310051E17 gene /// Kruppel-like factor 9 |
| 1422458_at | Tcl1 | T-cell lymphoma breakpoint 1 |
| 1422470_at | Bnip3 | BCL2/adenovirus E1B interacting protein 3 |
| 1422537_a_at | Id2 | inhibitor of DNA binding 2 |
| 1422557_s_at | Mt1 | metallothionein 1 |
| 1422610_s_at | Igf2bp3 | insulin-like growth factor 2 mRNA binding protein 3 |
| 1422611_s_at | Igf2bp3 | insulin-like growth factor 2 mRNA binding protein 3 |
| 1422660_at | Rbm3 | RNA binding motif protein 3 |
| 1422792_at | Pafah1b2 | platelet-activating factor acetylhydrolase, isoform 1b, |
| 1422851_at | Hmga2 | high mobility group AT-hook 2 |
| 1422962_a_at | Psmb8 | proteasome (prosome, macropain) subunit, beta type 8 |
| 1422967_a_at | Tfrc | transferrin receptor |
| 1422986_at | Esrrb | estrogen related receptor, beta |
| 1423049_a_at | Tpm1 | tropomyosin 1, alpha |
| 1423062_at | Igfbp3 | insulin-like growth factor binding protein 3 |
| 1423065_at | Dnmt3a | DNA methyltransferase 3A |
| 1423104_at | Irs1 | insulin receptor substrate 1 |
| 1423110_at | Col1a2 | collagen, type I, alpha 2 |
| 1423175_s_at | Pard6b | par-6 (partitioning defective 6) homolog beta (C. |
| 1423222_at | Cap2 | CAP, adenylate cyclase-associated protein, 2 (yeast) |
| 1423280_at | Stmn2 | stathmin-like 2 |

| | | |
|---|---|---|
| 1423281_at | Stmn2 | stathmin-like 2 |
| 1423393_at | Clic4 | chloride intracellular channel 4 (mitochondrial) |
| 1423416_at | Smarcc1 | SWI/SNF related, matrix associated, actin dependent |
| 1423424_at | Zic3 | zinc finger protein of the cerebellum 3 |
| 1423505_at | Tagln | transgelin |
| 1423523_at | Aass | aminoadipate-semialdehyde synthase |
| 1423586_at | Axl | AXL receptor tyrosine kinase |
| 1423606_at | Postn | periostin, osteoblast specific factor |
| 1423686_a_at | Prr13 | proline rich 13 |
| 1423691_x_at | Krt8 | keratin 8 |
| 1423694_at | Kctd10 | potassium channel tetramerisation domain containing |
| 1423721_at | Tpm1 | tropomyosin 1, alpha |
| 1423722_at | Tmem49 | transmembrane protein 49 |
| 1423804_a_at | Idi1 | isopentenyl-diphosphate delta isomerase |
| 1423805_at | Dab2 | disabled homolog 2 (Drosophila) |
| 1423824_at | Gpr177 | G protein-coupled receptor 177 |
| 1423900_at | Trip12 | thyroid hormone receptor interactor 12 |
| 1423952_a_at | Krt7 | keratin 7 |
| 1424051_at | Col4a2 | collagen, type IV, alpha 2 |
| 1424075_at | 9430016H08Rik | RIKEN cDNA 9430016H08 gene |
| 1424163_at | Rmnd5b | required for meiotic nuclear division 5 homolog B (S. |
| 1424169_at | OTTMUSG000000128 93 /// Tax1bp3 | predicted gene, OTTMUSG00000012893 /// Tax1 (human T-cell leukemia virus type I) binding protein 3 |
| 1424239_at | 2310066E14Rik | RIKEN cDNA 2310066E14 gene |
| 1424263_at | 2810003C17Rik | RIKEN cDNA 2810003C17 gene |
| 1424269_a_at | EG433297 /// EG667952 /// Myl6 | predicted gene, EG433297 /// predicted gene, EG667952 /// myosin, light polypeptide 6, alkali, smooth |
| 1424309_a_at | Mocs2 | molybdenum cofactor synthesis 2 |
| 1424398_at | Dhx36 | DEAH (Asp-Glu-Ala-His) box polypeptide 36 |
| 1424531_a_at | Tcea3 | transcription elongation factor A (SII), 3 |
| 1424641_a_at | Thoc1 | THO complex 1 |
| 1424713_at | Calml4 | calmodulin-like 4 |
| 1424726_at | Tmem150 | transmembrane protein 150 |
| 1424740_at | Creb3 | cAMP responsive element binding protein 3 |
| 1424741_s_at | Creb3 | cAMP responsive element binding protein 3 |
| 1424768_at | Cald1 | caldesmon 1 |
| 1424769_s_at | Cald1 | caldesmon 1 |
| 1424770_at | Cald1 | caldesmon 1 |
| 1424792_at | Rpp40 | ribonuclease P 40 subunit (human) |
| 1424797_a_at | Pitx2 | paired-like homeodomain transcription factor 2 |
| 1425458_a_at | Grb10 | growth factor receptor bound protein 10 |
| 1425565_at | Rest | RE1-silencing transcription factor |
| 1425810_a_at | Csrp1 | cysteine and glycine-rich protein 1 |
| 1425895_a_at | Id1 | inhibitor of DNA binding 1 |
| 1426010_a_at | Epb4.1l3 | erythrocyte protein band 4.1-like 3 |
| 1426186_a_at | Fgf5 | fibroblast growth factor 5 |
| 1426195_a_at | Cst3 | cystatin C |
| 1426208_x_at | Plagl1 | pleiomorphic adenoma gene-like 1 |
| 1426225_at | Rbp4 | retinol binding protein 4, plasma |
| 1426332_a_at | Cldn3 | claudin 3 |
| 1426348_at | Col4a1 | collagen, type IV, alpha 1 |

| | | |
|---|---|---|
| 1426371_at | Far1 | fatty acyl CoA reductase 1 |
| 1426510_at | LOC100044896 /// Sccpdh | similar to Saccharopine dehydrogenase (putative) /// saccharopine dehydrogenase (putative) |
| 1426519_at | P4ha1 | procollagen-proline, 2-oxoglutarate 4-dioxygenase |
| 1426559_at | Sbno1 | sno, strawberry notch homolog 1 (Drosophila) |
| 1426598_at | Uty | ubiquitously transcribed tetratricopeptide repeat gene, Y |
| 1426614_at | Prkcbp1 | protein kinase C binding protein 1 |
| 1426696_at | Lrpap1 | low density lipoprotein receptor-related protein |
| 1426716_at | Tdrd7 | tudor domain containing 7 |
| 1426753_at | Phf17 | PHD finger protein 17 |
| 1426801_at | 8-Sep | septin 8 |
| 1426968_a_at | Rdh10 | retinol dehydrogenase 10 (all-trans) |
| 1427094_at | Pole2 | polymerase (DNA directed), epsilon 2 (p59 subunit) |
| 1427108_at | 9530068E07Rik | RIKEN cDNA 9530068E07 gene |
| 1427143_at | Jarid1b | jumonji, AT rich interactive domain 1B (Rbp2 like) |
| 1427174_at | 100039000 /// 3100002L24Rik /// OTTMUSG00000016219 /// OTTMUSG00000016325 /// OTTMUSG00000016327 /// OTTMUSG00000016609 /// OTTMUSG00000016611 | predicted gene, 100039000 /// RIKEN cDNA 3100002L24 gene /// predicted gene, OTTMUSG00000016219 /// predicted gene, OTTMUSG00000016325 /// predicted gene, OTTMUSG00000016327 /// predicted gene, OTTMUSG00000016609 /// predicted gene, OTTMUSG00000016611 |
| 1427197_at | Atr | ataxia telangiectasia and Rad3 related |
| 1427266_at | Pbrm1 | polybromo 1 |
| 1427382_a_at | Suv39h1 | suppressor of variegation 3-9 homolog 1 (Drosophila) |
| 1427442_a_at | App | amyloid beta (A4) precursor protein |
| 1427477_at | Tmprss13 | transmembrane protease, serine 13 |
| 1427539_a_at | Zwint | ZW10 interactor |
| 1427735_a_at | Acta1 | actin, alpha 1, skeletal muscle |
| 1427760_s_at | Prl2c2 /// Prl2c3 /// Prl2c4 | prolactin family 2, subfamily c, member 2 /// prolactin family 2, subfamily c, member 3 /// prolactin family 2, |
| 1427768_s_at | Myl3 | myosin, light polypeptide 3 |
| 1427872_at | Ptafr | platelet-activating factor receptor |
| 1427883_a_at | Col3a1 | collagen, type III, alpha 1 |
| 1428079_at | Fgb | fibrinogen, B beta polypeptide |
| 1428107_at | Sh3bgrl | SH3-binding domain glutamic acid-rich protein like |
| 1428272_at | Eif1b | eukaryotic translation initiation factor 1B |
| 1428289_at | 2310051E17Rik /// Klf9 | RIKEN cDNA 2310051E17 gene /// Kruppel-like factor 9 |

| | | |
|---|---|---|
| 1428301_at | 100041195 /// 100041874 /// 666442 /// 666637 /// ENSMUSG00000063277 /// ENSMUSG00000068790 /// ENSMUSG00000072735 /// LOC100036568 /// LOC544988 /// LOC671957 | predicted gene, 100041195 /// predicted gene, 100041874 /// predicted gene, 666442 /// predicted gene, 666637 /// predicted gene, ENSMUSG00000063277 /// predicted gene, ENSMUSG00000068790 /// predicted gene, ENSMUSG00000072735 /// hypothetical LOC100036568 /// hypothetical protein LOC544988 /// hypothetical protein LOC671957 |
| 1428310_at | Larp7 | La ribonucleoprotein domain family, member 7 |
| 1428405_at | Hcfc1r1 | host cell factor C1 regulator 1 (XPO1-dependent) |
| 1428406_s_at | Hcfc1r1 | host cell factor C1 regulator 1 (XPO1-dependent) |
| 1428842_a_at | Ngfrap1 | nerve growth factor receptor (TNFRSF16) associated |
| 1428853_at | Ptch1 | patched homolog 1 |
| 1428942_at | Mt2 | metallothionein 2 |
| 1429177_x_at | Sox17 | SRY-box containing gene 17 |
| 1429265_a_at | Rnf130 | ring finger protein 130 |
| 1429859_a_at | Arl2bp | ADP-ribosylation factor-like 2 binding protein |
| 1429888_a_at | Hspb2 | heat shock protein 2 |
| 1430634_a_at | Pfkp | phosphofructokinase, platelet |
| 1431036_a_at | ENSMUSG00000074460 /// Tmed2 | predicted gene, ENSMUSG00000074460 /// transmembrane emp24 domain trafficking protein 2 |
| 1431292_a_at | Twf2 | twinfilin, actin-binding protein, homolog 2 (Drosophila) |
| 1431417_at | Jam2 | junction adhesion molecule 2 |
| 1431429_a_at | Arl4a | ADP-ribosylation factor-like 4A |
| 1431805_a_at | Rhpn2 | rhophilin, Rho GTPase binding protein 2 |
| 1431900_a_at | Foxa3 | forkhead box A3 |
| 1432057_a_at | Prdm5 | PR domain containing 5 |
| 1432466_a_at | Apoe | apolipoprotein E |
| 1432828_at | --- | --- |
| 1433428_x_at | Tgm2 | transglutaminase 2, C polypeptide |
| 1433508_at | Klf6 | Kruppel-like factor 6 |
| 1433519_at | Nucks1 | nuclear casein kinase and cyclin-dependent kinase |
| 1433720_s_at | Chchd10 | coiled-coil-helix-coiled-coil-helix domain containing 10 |
| 1433883_at | Tpm4 | tropomyosin 4 |
| 1433924_at | --- | --- |
| 1434005_at | Rbms1 | RNA binding motif, single stranded interacting protein 1 |
| 1434025_at | --- | --- |
| 1434036_at | Mtss1 | metastasis suppressor 1 |
| 1434369_a_at | Cryab | crystallin, alpha B |
| 1434436_at | Morc4 | microrchidia 4 |
| 1434489_at | Elmo3 | engulfment and cell motility 3, ced-12 homolog (C. |
| 1434503_s_at | Lamp2 | lysosomal-associated membrane protein 2 |
| 1434637_x_at | Sin3b | transcriptional regulator, SIN3B (yeast) |
| 1434853_x_at | Mkrn1 | makorin, ring finger protein, 1 |
| 1434987_at | Aldh2 | aldehyde dehydrogenase 2, mitochondrial |
| 1434988_x_at | Aldh2 | aldehyde dehydrogenase 2, mitochondrial |
| 1435176_a_at | Id2 | inhibitor of DNA binding 2 |

| | | |
|---|---|---|
| 1435194_at | Hspa4 | heat shock protein 4 |
| 1435275_at | Cox6b2 | cytochrome c oxidase subunit VIb polypeptide 2 |
| 1435382_at | Ndn | necdin |
| 1435493_at | Dsp | desmoplakin |
| 1435494_s_at | Dsp | desmoplakin |
| 1435561_at | Erf | Ets2 repressor factor |
| 1435783_at | B230112C05Rik | RIKEN cDNA B230112C05 gene |
| 1435989_x_at | Krt8 | keratin 8 |
| 1436291_a_at | Dpys | dihydropyrimidinase |
| 1436451_a_at | ENSMUSG000000744 60 /// Tmed2 | predicted gene, ENSMUSG00000074460 /// transmembrane emp24 domain trafficking protein 2 |
| 1436512_at | Arl4c /// LOC632433 | ADP-ribosylation factor-like 4C /// similar to ADP- |
| 1436609_a_at | Lrpap1 | low density lipoprotein receptor-related protein |
| 1436714_at | Lpp | LIM domain containing preferred translocation partner |
| 1436736_x_at | D0H4S114 | DNA segment, human D4S114 |
| 1436790_a_at | Sox11 | SRY-box containing gene 11 |
| 1436838_x_at | Cotl1 | coactosin-like 1 (Dictyostelium) |
| 1436879_x_at | Afp | alpha fetoprotein |
| 1436926_at | Esrrb | estrogen related receptor, beta |
| 1436990_s_at | Chchd10 | coiled-coil-helix-coiled-coil-helix domain containing 10 |
| 1437015_x_at | Pla2g1b | phospholipase A2, group IB, pancreas |
| 1437110_at | 2810474O19Rik | RIKEN cDNA 2810474O19 gene |
| 1437171_x_at | Gsn | gelsolin |
| 1437239_x_at | Phc2 | polyhomeotic-like 2 (Drosophila) |
| 1437277_x_at | Tgm2 | transglutaminase 2, C polypeptide |
| 1437279_x_at | Sdc1 | syndecan 1 |
| 1437308_s_at | F2r | coagulation factor II (thrombin) receptor |
| 1437434_a_at | Gpr177 | G protein-coupled receptor 177 |
| 1437502_x_at | Cd24a /// EG621324 | CD24a antigen /// predicted gene, EG621324 |
| 1437503_a_at | Shisa5 | shisa homolog 5 (Xenopus laevis) |
| 1437527_x_at | Mcl1 | myeloid cell leukemia sequence 1 |
| 1437689_x_at | Clu | clusterin |
| 1437845_x_at | Pofut2 | protein O-fucosyltransferase 2 |
| 1438116_x_at | Slc9a3r1 | solute carrier family 9 (sodium/hydrogen exchanger), |
| 1438118_x_at | Vim | vimentin |
| 1438133_a_at | Cyr61 | cysteine rich protein 61 |
| 1438321_x_at | 4930504E06Rik | RIKEN cDNA 4930504E06 gene |
| 1438386_x_at | Mat2a | methionine adenosyltransferase II, alpha |
| 1438840_x_at | Apoa1 | apolipoprotein A-I |
| 1438932_at | Rasgrp2 | RAS, guanyl releasing protein 2 |
| 1438945_x_at | Gja1 | gap junction protein, alpha 1 |
| 1438973_x_at | Gja1 | gap junction protein, alpha 1 |
| 1439440_x_at | Twf2 | twinfilin, actin-binding protein, homolog 2 (Drosophila) |
| 1442028_at | B4galnt2 | beta-1,4-N-acetyl-galactosaminyl transferase 2 |
| 1447977_x_at | 3100002L24Rik /// OTTMUSG000000166 09 /// OTTMUSG000000166 11 | RIKEN cDNA 3100002L24 gene /// predicted gene, OTTMUSG00000016609 /// predicted gene, OTTMUSG00000016611 |
| 1448152_at | Igf2 | insulin-like growth factor 2 |
| 1448169_at | Krt18 | keratin 18 |

| | | |
|---|---|---|
| 1448182_a_at | Cd24a /// EG621324 | CD24a antigen /// predicted gene, EG621324 |
| | AU021838 /// Mipol1 /// | expressed sequence AU021838 /// mirror-image |
| 1448192_s_at | Prps1 | polydactyly gene 1 homolog (human) /// phosphoribosyl |
| 1448194_a_at | H19 | H19 fetal liver mRNA |
| 1448361_at | Ttc3 | tetratricopeptide repeat domain 3 |
| 1448392_at | Sparc | secreted acidic cysteine rich glycoprotein |
| 1448393_at | Cldn7 | claudin 7 |
| 1448445_at | Acp6 | acid phosphatase 6, lysophosphatidic |
| 1448482_at | Slc39a8 | solute carrier family 39 (metal ion transporter), member |
| 1448524_s_at | Ssr4 | signal sequence receptor, delta |
| 1448562_at | Upp1 | uridine phosphorylase 1 |
| 1448688_at | Podxl | podocalyxin-like |
| 1448704_s_at | H47 | histocompatibility 47 |
| 1448705_at | Zbtb22 | zinc finger and BTB domain containing 22 |
| 1448729_a_at | 4-Sep | septin 4 |
| 1448732_at | Ctsb | cathepsin B |
| 1448737_at | Tspan7 | tetraspanin 7 |
| 1448752_at | Car2 | carbonic anhydrase 2 |
| 1448845_at | Rpp25 | ribonuclease P 25 subunit (human) |
| 1448873_at | Ocln | occludin |
| 1448883_at | Lgmn | legumain |
| 1448890_at | Klf2 | Kruppel-like factor 2 (lung) |
| 1448904_at | D6Wsu176e | DNA segment, Chr 6, Wayne State University 176, |
| 1448949_at | Car4 | carbonic anhydrase 4 |
| 1448964_at | S100g | S100 calcium binding protein G |
| 1448991_a_at | Ina /// LOC100047943 | internexin neuronal intermediate filament protein, alpha /// similar to Ina protein |
| 1449027_at | Rhou | ras homolog gene family, member U |
| 1449031_at | Cited1 | Cbp/p300-interacting transactivator with Glu/Asp-rich |
| 1449032_at | Prl2a1 | prolactin family 2, subfamily a, member 1 |
| 1449036_at | Rnf128 | ring finger protein 128 |
| 1449048_s_at | Rab4a | RAB4A, member RAS oncogene family |
| 1449064_at | Tdh | L-threonine dehydrogenase |
| 1449106_at | Gpx3 | glutathione peroxidase 3 |
| 1449119_at | Arih2 | ariadne homolog 2 (Drosophila) |
| 1449168_a_at | Akap2 /// Palm2 /// RP23-334A5.2 | A kinase (PRKA) anchor protein 2 /// paralemmin 2 /// Palm2-Akap2 protein |
| 1449244_at | Cdh2 /// LOC100044363 | cadherin 2 /// similar to N-cadherin |
| 1449254_at | Spp1 | secreted phosphoprotein 1 |
| 1449289_a_at | B2m | beta-2 microglobulin |
| 1449363_at | Atf3 | activating transcription factor 3 |
| 1449369_at | Tmprss2 | transmembrane protease, serine 2 |
| 1449408_at | Jam2 | junction adhesion molecule 2 |
| 1449455_at | Hck | hemopoietic cell kinase |
| 1449729_at | Fgf4 | Fibroblast growth factor 4 |
| 1449738_s_at | D3Ertd300e | DNA segment, Chr 3, ERATO Doi 300, expressed |
| 1449875_s_at | H2-T10 /// H2-T17 /// H2-T22 /// H2-T9 | histocompatibility 2, T region locus 10 /// histocompatibility 2, T region locus 17 /// |
| 1449929_at | Dynlt3 | dynein light chain Tctex-type 3 |
| 1449939_s_at | Dlk1 | delta-like 1 homolog (Drosophila) |

| | | |
|---|---|---|
| 1449942_a_at | Ilk | integrin linked kinase |
| 1450021_at | Ubqln2 | ubiquilin 2 |
| 1450079_at | Nrk | Nik related kinase |
| 1450089_a_at | Srprb | signal recognition particle receptor, B subunit |
| 1450186_s_at | Gnas | GNAS (guanine nucleotide binding protein, alpha |
| 1450264_a_at | Chka | choline kinase alpha |
| | | |
| 1450377_at | LOC640441 /// Thbs1 | similar to thrombospondin 1 /// thrombospondin 1 |
| 1450397_at | Mtap1b | microtubule-associated protein 1B |
| 1450398_at | Kcnk5 | potassium channel, subfamily K, member 5 |
| 1450482_a_at | Pitx2 | paired-like homeodomain transcription factor 2 |
| 1450567_a_at | Col2a1 | collagen, type II, alpha 1 |
| 1450626_at | Manba | mannosidase, beta A, lysosomal |
| 1450650_at | Myo10 | myosin X |
| 1450710_at | Jarid2 | jumonji, AT rich interactive domain 2 |
| 1450780_s_at | Hmga2 | high mobility group AT-hook 2 |
| 1450781_at | Hmga2 | high mobility group AT-hook 2 |
| 1450839_at | D0H4S114 | DNA segment, human D4S114 |
| 1450843_a_at | Serpinh1 | serine (or cysteine) peptidase inhibitor, clade H, |
| 1450851_at | Wdr1 | WD repeat domain 1 |
| 1450857_a_at | Col1a2 | collagen, type I, alpha 2 |
| 1450872_s_at | Lipa | lysosomal acid lipase A |
| 1450971_at | Gadd45b | growth arrest and DNA-damage-inducible 45 beta |
| 1450981_at | Cnn2 | calponin 2 |
| 1450989_at | Tdgf1 | teratocarcinoma-derived growth factor 1 |
| 1450990_at | Gpc3 | glypican 3 |
| 1451021_a_at | Klf5 | Kruppel-like factor 5 |
| 1451219_at | Ormdl1 | ORM1-like 1 (S. cerevisiae) |
| 1451230_a_at | Wbp5 | WW domain binding protein 5 |
| 1451310_a_at | Ctsl | cathepsin L |
| 1451458_at | Tmem2 | transmembrane protein 2 |
| 1451580_a_at | Ttr | transthyretin |
| 1451931_x_at | H2-L | histocompatibility 2, D region |
| 1451968_at | Xrcc5 | X-ray repair complementing defective repair in Chinese |
| 1452035_at | Col4a1 | collagen, type IV, alpha 1 |
| 1452050_at | Camk1d | calcium/calmodulin-dependent protein kinase ID |
| 1452092_at | 4631426J05Rik | RIKEN cDNA 4631426J05 gene |
| 1452141_a_at | Sepp1 | selenoprotein P, plasma, 1 |
| 1452148_at | Lrpap1 | low density lipoprotein receptor-related protein |
| 1452165_at | Prl2b1 | prolactin family 2, subfamily b, member 1 |
| 1452180_at | Phf17 | PHD finger protein 17 |
| 1452207_at | Cited2 | Cbp/p300-interacting transactivator, with Glu/Asp-rich |
| 1452217_at | Ahnak | AHNAK nucleoprotein (desmoyokin) |
| 1452287_at | --- | --- |
| 1452294_at | Pcdh1 | protocadherin 1 |
| 1452297_at | Ccnt2 | cyclin T2 |
| 1452387_a_at | Amotl2 | angiomotin-like 2 |
| 1452428_a_at | B2m | beta-2 microglobulin |
| 1452670_at | Myl9 | myosin, light polypeptide 9, regulatory |
| 1452716_at | 5730469M10Rik | RIKEN cDNA 5730469M10 gene |

| | | |
|---|---|---|
| 1452731_x_at | 100040880 /// 100041195 /// 100041874 /// 100042164 /// 666442 /// 666637 /// B930046C15Rik /// ENSMUSG00000063277 /// ENSMUSG00000068790 /// ENSMUSG00000072735 /// LOC100036568 /// LOC100041530 /// LOC671957 | predicted gene, 100040880 /// predicted gene, 100041195 /// predicted gene, 100041874 /// predicted gene, 100042164 /// predicted gene, 666442 /// predicted gene, 666637 /// RIKEN cDNA B930046C15 gene /// predicted gene, ENSMUSG00000063277 /// predicted gene, ENSMUSG00000068790 /// predicted gene, ENSMUSG00000072735 /// hypothetical LOC100036568 /// similar to 1700001E04Rik protein /// hypothetical protein LOC671957 |
| 1453004_at | Slc22a23 | solute carrier family 22, member 23 |
| 1453223_s_at | Dppa2 | developmental pluripotency associated 2 |
| 1453556_x_at | Cd99 | CD99 antigen |
| 1453957_a_at | Igf2bp3 | insulin-like growth factor 2 mRNA binding protein 3 |
| 1454064_a_at | Rnf138 | ring finger protein 138 |
| 1454268_a_at | Cyba | cytochrome b-245, alpha polypeptide |
| 1454608_x_at | Ttr | transthyretin |
| 1454838_s_at | AW548124 /// LOC100048505 | expressed sequence AW548124 /// similar to Expressed sequence AW548124 |
| 1454849_x_at | Clu | clusterin |
| 1454890_at | Amot | angiomotin |
| 1454946_at | Mybl2 | myeloblastosis oncogene-like 2 |
| 1455084_x_at | Shmt2 | serine hydroxymethyltransferase 2 (mitochondrial) |
| 1455099_at | Mogat2 | monoacylglycerol O-acyltransferase 2 |
| 1455201_x_at | Apoa1 | apolipoprotein A-I |
| 1455540_at | Cps1 | carbamoyl-phosphate synthetase 1 |
| 1455792_x_at | Ndn | necdin |
| 1455871_s_at | OTTMUSG00000012893 /// Rpl13 /// Tax1bp3 | predicted gene, OTTMUSG00000012893 /// ribosomal protein L13 /// Tax1 (human T-cell leukemia virus type I) binding protein 3 |
| 1455913_x_at | Ttr | transthyretin |
| 1455940_x_at | Wdr6 | WD repeat domain 6 |
| 1456014_s_at | Fermt3 | fermitin family homolog 3 (Drosophila) |
| 1456085_x_at | Cd151 | CD151 antigen |
| 1456112_at | LOC100043998 /// Tpr | similar to nuclear pore complex-associated intranuclear coiled-coil protein TPR /// translocated promoter region |
| 1456174_x_at | Ndrg1 | N-myc downstream regulated gene 1 |
| 1456243_x_at | Mcl1 | myeloid cell leukemia sequence 1 |
| 1456312_x_at | Gsn | gelsolin |
| 1456341_a_at | 2310051E17Rik /// Klf9 | RIKEN cDNA 2310051E17 gene /// Kruppel-like factor 9 |
| 1456381_x_at | Mcl1 | myeloid cell leukemia sequence 1 |
| 1456424_s_at | Pltp | phospholipid transfer protein |
| 1456511_x_at | Eras | ES cell-expressed Ras |
| 1456530_x_at | Elovl1 | elongation of very long chain fatty acids (FEN1/Elo2, |
| 1456573_x_at | Nnt | nicotinamide nucleotide transhydrogenase |

| | | |
|---|---|---|
| 1456590_x_at | Akr1b3 | aldo-keto reductase family 1, member B3 (aldose |
| 1456623_at | Tpm1 | tropomyosin 1, alpha |
| 1456626_a_at | 1110005A23Rik /// EG625193 | RIKEN cDNA 1110005A23 gene /// predicted gene, EG625193 |
| 1456733_x_at | Serpinh1 | serine (or cysteine) peptidase inhibitor, clade H, |
| 1460217_at | Prl7d1 | prolactin family 7, subfamily d, member 1 |
| 1460302_at | Thbs1 | thrombospondin 1 |
| 1460314_s_at | Hist1h3a /// Hist1h3b /// Hist1h3c /// Hist1h3d /// Hist1h3e /// Hist1h3f /// Hist1h3g /// Hist1h3h /// Hist1h3i /// Hist2h3b /// Hist2h3c1 /// Hist2h3c2 | histone cluster 1, H3a /// histone cluster 1, H3b /// histone cluster 1, H3c /// histone cluster 1, H3d /// histone cluster 1, H3e /// histone cluster 1, H3f /// histone cluster 1, H3g /// histone cluster 1, H3h /// histone cluster 1, H3i /// histone cluster 2, H3b /// histone cluster 2, H3c1 /// histone cluster 2, H3c2 |
| 1460330_at | Anxa3 | annexin A3 |
| 1460411_s_at | AW548124 /// LOC100048505 | expressed sequence AW548124 /// similar to Expressed sequence AW548124 |
| 1460430_at | LOC100047558 /// Rap2c | similar to RAP2C, member of RAS oncogene family /// RAP2C, member of RAS oncogene family |
| 1460561_x_at | Sepw1 | selenoprotein W, muscle 1 |
