# Supplementary material for: Knowledge-guided gene ranking by coordinative component analysis
Source: BMC Bioinformatics. 2010 Mar 30;11:162. doi: 10.1186/1471-2105-11-162 (PMC2865494; doi:10.1186/1471-2105-11-162)
Supplement: Additional file 5 — The top 500 probe sets ranked by WNT pathway-guided COCA approach. [file 1471-2105-11-162-S5.PDF]

Probe Set ID	Gene Symbol	Gene Name
1415697_at	G3bp2	GTPase activating protein (SH3 domain) binding protein 2
1415726_at	Ankrd17	ankyrin repeat domain 17
1415772_at	Ncl	nucleolin
1415806_at	Plat	plasminogen activator, tissue
1415808_at	Tpbpa	trophoblast specific protein alpha
1415812_at	Gsn	gelsolin
1415835_at	Prl3b1	prolactin family 3, subfamily b, member 1
1415848_at	Prl3d1	prolactin family 3, subfamily d, member 1
1415906_at	Tmsb4x	thymosin, beta 4, X chromosome
1415938_at	Spink3	serine peptidase inhibitor, Kazal type 3
1415943_at	Sdc1	syndecan 1
1415971_at	Marcks	myristoylated alanine rich protein kinase C substrate
1415972_at	Marcks	myristoylated alanine rich protein kinase C substrate
1415973_at	Marcks	myristoylated alanine rich protein kinase C substrate
1415983_at	Lcp1	lymphocyte cytosolic protein 1
1415987_at	Hdlbp	high density lipoprotein (HDL) binding protein
1415996_at	Txnip	thioredoxin interacting protein
1415997_at	Txnip	thioredoxin interacting protein
1416021_a_at	EG620603 /// Fabp5	predicted gene, EG620603 /// fatty acid binding protein 5, epidermal
1416023_at	Fabp3	fatty acid binding protein 3, muscle and heart
1416029_at	Klf10	Kruppel-like factor 10
1416034_at	Cd24a /// EG621324	CD24a antigen /// predicted gene, EG621324
1416039_x_at	Cyr61	cysteine rich protein 61
1416122_at	Ccnd2	cyclin D2
1416155_at	Hmgb3	high mobility group box 3
1416183_a_at	Ldhb	lactate dehydrogenase B
1416211_a_at	Ptn	pleiotrophin
1416401_at	Cd82	CD82 antigen
1416405_at	Bgn	biglycan
1416413_at	Ctsj	cathepsin J
1416416_x_at	Gstm1	glutathione S-transferase, mu 1
1416444_at	Elovl2	elongation of very long chain fatty acids (FEN1/Elo2, SUR4/Elo3, yeast)-like 2
1416455_a_at	Cryab	crystallin, alpha B
1416589_at	Sparc	secreted acidic cysteine rich glycoprotein
1416614_at	Eid1	EP300 interacting inhibitor of differentiation 1

1416617_at	Acss1	acyl-CoA synthetase short-chain family member 1
1416630_at	Id3	inhibitor of DNA binding 3
1416637_at	Slc4a2	solute carrier family 4 (anion exchanger), member 2
1416645_a_at	Afp	alpha fetoprotein
1416646_at	Afp	alpha fetoprotein
1416658_at	Frzb	frizzled-related protein
1416673_at	Bace2	beta-site APP-cleaving enzyme 2
1416686_at	Plod2	procollagen lysine, 2-oxoglutarate 5-dioxygenase 2
1416687_at	Plod2	procollagen lysine, 2-oxoglutarate 5-dioxygenase 2
1416737_at	Gys1	glycogen synthase 1, muscle
1416761_at	Hsd11b2	hydroxysteroid 11-beta dehydrogenase 2
1416767_a_at	1110003E01Rik	RIKEN cDNA 1110003E01 gene
1416808_at	Nid1	nidogen 1
1416832_at	Slc39a8	solute carrier family 39 (metal ion transporter), member 8
1416920_at	Rbm4	RNA binding motif protein 4
1416926_at	Trp53inp1	transformation related protein 53 inducible nuclear protein 1
1416953_at	Ctgf	connective tissue growth factor
1416965_at	Pcsk1n	proprotein convertase subtilisin/kexin type 1 inhibitor
1416967_at	Sox2	SRY-box containing gene 2
1417078_at	Lgals2	lectin, galactose-binding, soluble 2
1417079_s_at	Lgals2	lectin, galactose-binding, soluble 2
1417092_at	Pthr1	parathyroid hormone receptor 1
1417109_at	Tinagl1	tubulointerstitial nephritis antigen-like 1
1417110_at	Man1a	mannosidase 1, alpha
1417111_at	Man1a	mannosidase 1, alpha
1417133_at	Pmp22	peripheral myelin protein 22
1417149_at	P4ha2	procollagen-proline, 2-oxoglutarate 4-dioxygenase (proline 4-hydroxylase), alpha II polypeptide
1417156_at	Krt19	keratin 19
1417175_at	Csnk1e	casein kinase 1, epsilon
1417176_at	Csnk1e	casein kinase 1, epsilon
1417316_at	Them2	thioesterase superfamily member 2
1417343_at	Fxyd6	FXYD domain-containing ion transport regulator 6
1417346_at	Pycard	PYD and CARD domain containing
1417355_at	Peg3	paternally expressed 3
1417356_at	Peg3	paternally expressed 3
1417394_at	Klf4	Kruppel-like factor 4 (gut)
1417395_at	Klf4	Kruppel-like factor 4 (gut)
1417408_at	F3	coagulation factor III
1417419_at	Ccnd1	cyclin D1
1417420_at	Ccnd1	cyclin D1

1417500_a_at	Tgm2	transglutaminase 2, C polypeptide
1417553_at	Plac1	placental specific protein 1
1417566_at	Abhd5	abhydrolase domain containing 5
1417649_at	Cdkn1c	cyclin-dependent kinase inhibitor 1C (P57)
1417654_at	Sdc4	syndecan 4
1417730_at	Ext1	exostoses (multiple) 1
1417738_at	Rab25	RAB25, member RAS oncogene family
1417741_at	Pygl	liver glycogen phosphorylase
1417821_at	D17H6S56E-5	DNA segment, Chr 17, human D6S56E 5
1417928_at	Pdlim4	PDZ and LIM domain 4
1417950_a_at	Apoa2	apolipoprotein A-II
1417976_at	Ada	adenosine deaminase
1418069_at	Apoc2	apolipoprotein C-II
1418144_a_at	Pip5k1a	phosphatidylinositol-4-phosphate 5-kinase, type 1 alpha
1418153_at	Lama1	laminin, alpha 1
1418191_at	LOC100048346 /// Usp18	similar to ubiquitin specific protease UBP43 /// ubiquitin specific peptidase 18
1418237_s_at	Col18a1	collagen, type XVIII, alpha 1
1418283_at	Cldn4	claudin 4
1418364_a_at	EG665937 /// Ftl1 /// Ftl2 /// mCG_17237	predicted gene, EG665937 /// ferritin light chain 1 /// ferritin light chain 2 /// ferritin light chain 1 pseudogene
1418374_at	Fxyd3	FXYD domain-containing ion transport regulator 3
1418386_at	N6amt2	N-6 adenine-specific DNA methyltransferase 2 (putative)
1418422_at	Serpinb9g	serine (or cysteine) peptidase inhibitor, clade B, member 9g
1418423_s_at	OTTMUSG00000000724 /// Serpinb9e /// Serpinb9f /// Serpinb9g	predicted gene, OTTMUSG00000000724 /// serine (or cysteine) peptidase inhibitor, clade B, member 9e /// serine (or cysteine) peptidase inhibitor, clade B, member 9f /// serine (or cysteine) peptidase inhibitor, clade B, member 9g
1418455_at	Copz2	coatomer protein complex, subunit zeta 2
1418486_at	Vnn1	vanin 1
1418538_at	Kdelr3	KDEL (Lys-Asp-Glu-Leu) endoplasmic reticulum protein retention receptor 3
1418626_a_at	Clu	clusterin
1418703_at	Rbms1	RNA binding motif, single stranded interacting protein 1
1418788_at	Tek	endothelial-specific receptor tyrosine kinase

1418805_at	Sct	secretin
1418815_at	Cdh2 /// LOC100044363	cadherin 2 /// similar to N-cadherin
1418835_at	Phlda1	pleckstrin homology-like domain, family A, member 1
1418901_at	Cebpb	CCAAT/enhancer binding protein (C/EBP), beta
1418918_at	Igfbp1	insulin-like growth factor binding protein 1
1419018_at	Rhox6	reproductive homeobox 6
1419091_a_at	Anxa2	annexin A2
1419095_a_at	Apom	apolipoprotein M
1419096_at	Apom	apolipoprotein M
1419142_at	Ctsr	cathepsin R
1419149_at	Serpine1	serine (or cysteine) peptidase inhibitor, clade E, member 1
1419154_at	Tmprss2	transmembrane protease, serine 2
1419194_s_at	Gmfg	glia maturation factor, gamma
1419232_a_at	Apoa1	apolipoprotein A-I
1419233_x_at	Apoa1	apolipoprotein A-I
1419266_at	Nfyb	nuclear transcription factor-Y beta
1419273_at	C80913	expressed sequence C80913
1419300_at	Flt1	FMS-like tyrosine kinase 1
1419456_at	Dcxr	dicarbonyl L-xylulose reductase
1419513_a_at	Ect2	ect2 oncogene
1419606_a_at	Tnnt1	troponin T1, skeletal, slow
1419640_at	Purb	purine rich element binding protein B
1419641_at	Purb	purine rich element binding protein B
1419656_at	Slc25a36	solute carrier family 25, member 36
1419657_a_at	Slc25a36	solute carrier family 25, member 36
1419672_at	Spock1	sparc/osteonectin, cwcv and kazal-like domains proteoglycan 1
1419722_at	Klk8	kallikrein related-peptidase 8
1419767_at	Padi3	peptidyl arginine deiminase, type III
1419819_s_at	Sec63	SEC63-like (S. cerevisiae)
1419835_s_at	Plec1	plectin 1
1419872_at	Csf1r	colony stimulating factor 1 receptor
1420088_at	Nfkbia	nuclear factor of kappa light polypeptide gene enhancer in B-cells inhibitor, alpha
1420621_a_at	App	amyloid beta (A4) precursor protein
1420626_at	Gtf3c6	general transcription factor IIIC, polypeptide 6, alpha
1420647_a_at	Krt8	keratin 8
1420664_s_at	Procr	protein C receptor, endothelial
1420760_s_at	Ndrg1	N-myc downstream regulated gene 1
1420901_a_at	Hk1	hexokinase 1
1420913_at	Slco2a1	solute carrier organic anion transporter family, member 2a1
1421014_a_at	Clybl	citrate lyase beta like

1421022_x_at	Acyp1	acylphosphatase 1, erythrocyte (common) type
1421061_at	Guca1a	guanylate cyclase activator 1a (retina)
1421135_a_at	Cnot8	CCR4-NOT transcription complex, subunit 8
1421217_a_at	Lgals9	lectin, galactose binding, soluble 9
1421267_a_at	Cited2	Cbp/p300-interacting transactivator, with Glu/Asp-rich carboxy-terminal domain, 2
1421323_a_at	G3bp2	GTPase activating protein (SH3 domain) binding protein 2
1421365_at	Fst	follistatin
1421375_a_at	S100a6	S100 calcium binding protein A6 (calcyclin)
1421654_a_at	Lmna	lamin A
1421657_a_at	Sox17	SRY-box containing gene 17
1421750_a_at	Vbp1	von Hippel-Lindau binding protein 1
1421882_a_at	Elavl2	ELAV (embryonic lethal, abnormal vision, Drosophila)-like 2 (Hu antigen B)
1421917_at	Pdgfra	platelet derived growth factor receptor, alpha polypeptide
1422289_a_at	Ctsq	cathepsin Q
1422438_at	Ephx1	epoxide hydrolase 1, microsomal
1422462_at	Ube2t	ubiquitin-conjugating enzyme E2T (putative)
1422476_at	Ifi30	interferon gamma inducible protein 30
1422668_at	Serpinb9b	serine (or cysteine) peptidase inhibitor, clade B, member 9b
1422733_at	Fjx1	four jointed box 1 (Drosophila)
1422768_at	Syncrip	synaptotagmin binding, cytoplasmic RNA interacting protein
1422804_at	Serpinb6b	serine (or cysteine) peptidase inhibitor, clade B, member 6b
1422836_at	Mbnl3	muscleblind-like 3 (Drosophila)
1422851_at	Hmga2	high mobility group AT-hook 2
1422943_a_at	Hspb1	heat shock protein 1
1423026_at	Rad51c	Rad51 homolog c (S. cerevisiae)
1423054_at	Wdr1	WD repeat domain 1
1423063_at	Dnmt3a	DNA methyltransferase 3A
1423064_at	Dnmt3a	DNA methyltransferase 3A
1423066_at	Dnmt3a	DNA methyltransferase 3A
1423110_at	Col1a2	collagen, type I, alpha 2
1423187_at	Gabarapl2	gamma-aminobutyric acid (GABA-A) receptor-associated protein-like 2
1423259_at	Id4 /// LOC100045546	inhibitor of DNA binding 4 /// similar to Id4
1423271_at	Gjb2	gap junction protein, beta 2
1423294_at	Mest	mesoderm specific transcript

1423321_at	Myadm	myeloid-associated differentiation marker
1423404_at	Gkn1	gastrokine 1
1423413_at	Ndrg1	N-myc downstream regulated gene 1
1423429_at	Rhox5	reproductive homeobox 5
1423450_a_at	Hs3st1	heparan sulfate (glucosamine) 3-O-sulfotransferase 1
1423506_a_at	Nnat	neuronatin
1423523_at	Aass	aminoadipate-semialdehyde synthase
1423605_a_at	Mdm2	transformed mouse 3T3 cell double minute 2
1423606_at	Postn	periostin, osteoblast specific factor
1423669_at	Col1a1	collagen, type I, alpha 1
1423686_a_at	Prr13	proline rich 13
1423691_x_at	Krt8	keratin 8
1423747_a_at	Pdk1	pyruvate dehydrogenase kinase, isoenzyme 1
1423756_s_at	Igfbp4	insulin-like growth factor binding protein 4
1423758_at	G3bp2	GTPase activating protein (SH3 domain) binding protein 2
1423824_at	Gpr177	G protein-coupled receptor 177
1423883_at	Acsl1	acyl-CoA synthetase long-chain family member 1
1423885_at	Lamc1	laminin, gamma 1
1423933_a_at	1600029D21Rik	RIKEN cDNA 1600029D21 gene
1423952_a_at	Krt7	keratin 7
1424051_at	Col4a2	collagen, type IV, alpha 2
1424067_at	Icam1	intercellular adhesion molecule 1
1424072_at	2010107G23Rik	RIKEN cDNA 2010107G23 gene
1424086_at	Oaf	OAF homolog (Drosophila)
1424113_at	Lamb1-1	laminin B1 subunit 1
1424114_s_at	Lamb1-1	laminin B1 subunit 1
1424123_at	Flvcr2	feline leukemia virus subgroup C cellular receptor family, member 2
1424254_at	Ifitm1	interferon induced transmembrane protein 1
1424263_at	2810003C17Rik	RIKEN cDNA 2810003C17 gene
1424265_at	Npl	N-acetylneuraminate pyruvate lyase
1424295_at	Dppa3	developmental pluripotency-associated 3
1424309_a_at	Mocs2	molybdenum cofactor synthesis 2
1424351_at	Wfdc2	WAP four-disulfide core domain 2
1424355_a_at	Sin3b	transcriptional regulator, SIN3B (yeast)
1424367_a_at	Homer2	homer homolog 2 (Drosophila)
1424490_at	Zfp428	zinc finger protein 428
1424556_at	Pycr1	pyrroline-5-carboxylate reductase 1
1424572_a_at	H2afy	H2A histone family, member Y

1424607_a_at	100039204 /// 100040620 /// 100040790 /// 100041416 /// 100042151 /// 100043211 /// 100043316 /// BC003993 /// LOC100047648 /// OTTMUSG00000017752	predicted gene, 100039204 /// predicted gene, 100040620 /// predicted gene, 100040790 /// predicted gene, 100041416 /// predicted gene, 100042151 /// predicted gene, 100043211 /// predicted gene, 100043316 /// cDNA sequence BC003993 /// hypothetical protein LOC100047648 /// predicted gene, OTTMUSG00000017752
1424649_a_at	Tspan8	tetraspanin 8
1424657_at	Taok1	TAO kinase 1
1424759_at	Arrdc4	arrestin domain containing 4
1424769_s_at	Cald1	caldesmon 1
1424847_at	Nefh	neurofilament, heavy polypeptide
1424927_at	Glipr1	GLI pathogenesis-related 1 (glioma)
1424942_a_at	Myc	myelocytomatosis oncogene
1425106_a_at	Wars	tryptophanyl-tRNA synthetase
1425107_a_at	Lifr	leukemia inhibitory factor receptor
1425149_a_at	Pdcl	phosducin-like
1425220_x_at	LOC100038935	hypothetical protein LOC100038935
1425319_s_at	6530403A03Rik	RIKEN cDNA 6530403A03 gene
1425458_a_at	Grb10	growth factor receptor bound protein 10
1425464_at	Gata6	GATA binding protein 6
1425545_x_at	H2-D1	histocompatibility 2, D region locus 1
1425546_a_at	Trf	transferrin
1425567_a_at	Anxa5	annexin A5
1425991_a_at	Kank2	KN motif and ankyrin repeat domains 2
1425993_a_at	Hsph1	heat shock 105kDa/110kDa protein 1
1426088_at	---	---
1426208_x_at	Plagl1	pleiomorphic adenoma gene-like 1
1426225_at	Rbp4	retinol binding protein 4, plasma
1426243_at	Cth	cystathionase (cystathionine gamma-lyase)
1426255_at	Nefl	neurofilament, light polypeptide
1426348_at	Col4a1	collagen, type IV, alpha 1
1426370_at	Far1	fatty acyl CoA reductase 1
1426539_at	Usp11	ubiquitin specific peptidase 11
1426587_a_at	Stat3	signal transducer and activator of transcription 3
1426593_a_at	Fbxo22	F-box protein 22
1426682_at	Cnot6	CCR4-NOT transcription complex, subunit 6
1426683_at	Cnot6 /// LOC100046343	CCR4-NOT transcription complex, subunit 6 /// similar to CCR4-NOT transcription complex, subunit 6
1426808_at	Lgals3	lectin, galactose binding, soluble 3

1426951_at	Crim1	cysteine rich transmembrane BMP regulator 1 (chordin like)
1426980_s_at	E130012A19Rik	RIKEN cDNA E130012A19 gene
1426998_at	Zfand3	zinc finger, AN1-type domain 3
1427074_at	Pcmtd2	protein-L-isoaspartate (D-aspartate) O-methyltransferase domain containing 2
1427126_at	Hspa1b	heat shock protein 1B
1427127_x_at	Hspa1b	heat shock protein 1B
1427399_a_at	Nxf7	nuclear RNA export factor 7
1427442_a_at	App	amyloid beta (A4) precursor protein
1427474_s_at	Gstm3	glutathione S-transferase, mu 3
1427477_at	Tmprss13	transmembrane protease, serine 13
1427479_at	100039042 /// 100039129 /// 100042776 /// 627881 /// BB287469 /// EG266459 /// EG435337 /// EG544883 /// EG666862 /// Eif1a /// LOC100039226 /// LOC641136	predicted gene, 100039042 /// predicted gene, 100039129 /// predicted gene, 100042776 /// predicted gene, 627881 /// expressed sequence BB287469 /// predicted gene, EG266459 /// predicted gene, EG435337 /// predicted gene, EG544883 /// predicted gene, EG666862 /// eukaryotic translation initiation factor 1A /// hypothetical protein LOC100039226 /// similar to X-linked eukaryotic translation initiation factor 1A
1427481_a_at	Atp1a3	ATPase, Na+/K+ transporting, alpha 3 polypeptide
1427539_a_at	Zwint	ZW10 interactor
1427550_at	Peg10	paternally expressed 10
1427670_a_at	Tcf12	transcription factor 12
1427718_a_at	Mdm2	transformed mouse 3T3 cell double minute 2
1427760_s_at	Prl2c2 /// Prl2c3 /// Prl2c4	prolactin family 2, subfamily c, member 2 /// prolactin family 2, subfamily c, member 3 /// prolactin family 2, subfamily c, member 4
1427768_s_at	Myl3	myosin, light polypeptide 3
1427770_a_at	Slc2a3	solute carrier family 2 (facilitated glucose transporter), member 3
1427883_a_at	Col3a1	collagen, type III, alpha 1
1427887_at	Rprd1b	regulation of nuclear pre-mRNA domain containing 1B
1427960_at	Ugt2b34	UDP glucuronosyltransferase 2 family, polypeptide B34
1427961_s_at	Ugt2b34	UDP glucuronosyltransferase 2 family, polypeptide B34
1428061_at	Hat1	histone aminotransferase 1
1428079_at	Fgb	fibrinogen, B beta polypeptide
1428103_at	Adam10	a disintegrin and metallopeptidase domain 10

1428125_at	ENSMUSG00000074747	predicted gene, ENSMUSG00000074747
1428146_s_at	Acaa2	acetyl-Coenzyme A acyltransferase 2 (mitochondrial 3-oxoacyl-Coenzyme A thiolase)
1428306_at	Ddit4	DNA-damage-inducible transcript 4
1428572_at	Basp1 /// LOC100045716	brain abundant, membrane attached signal protein 1 /// similar to 22 kDa neuronal tissue-enriched acidic protein
1428706_at	Prr6	proline-rich polypeptide 6
1428853_at	Ptch1	patched homolog 1
1429177_x_at	Sox17	SRY-box containing gene 17
1429388_at	Nanog /// Nanogpd	Nanog homeobox /// similar to Nanog homeobox
1429483_at	Calcoco2	calcium binding and coiled-coil domain 2
1429654_at	Dppa2	developmental pluripotency associated 2
1430127_a_at	Ccnd2	cyclin D2
1430820_a_at	Bbx	bobby sox homolog (Drosophila)
1431701_a_at	Pdzk1	PDZ domain containing 1
1433428_x_at	Tgm2	transglutaminase 2, C polypeptide
1433471_at	Tcf7	transcription factor 7, T-cell specific
1433488_x_at	Gns	glucosamine (N-acetyl)-6-sulfatase
1433514_at	Etnk1	ethanolamine kinase 1
1433575_at	Sox4	SRY-box containing gene 4
1433720_s_at	Chchd10	coiled-coil-helix-coiled-coil-helix domain containing 10
1433924_at	---	---
1433956_at	Cdh5	cadherin 5
1434005_at	Rbms1	RNA binding motif, single stranded interacting protein 1
1434056_a_at	Ndufb6	NADH dehydrogenase (ubiquinone) 1 beta subcomplex, 6
1434353_at	Sfmbt2	Scm-like with four mbt domains 2
1434436_at	Morc4	microrchidia 4
1434499_a_at	Ldhb	lactate dehydrogenase B
1434513_at	Atp13a3	ATPase type 13A3
1434745_at	Ccnd2	cyclin D2
1434784_s_at	Tmem106c	transmembrane protein 106C
1434853_x_at	Mkrn1	makorin, ring finger protein, 1
1434924_at	Phf2	PHD finger protein 2
1434930_at	Tpcn1	two pore channel 1
1435176_a_at	Id2	inhibitor of DNA binding 2
1435275_at	Cox6b2	cytochrome c oxidase subunit VIb polypeptide 2
1435383_x_at	Ndn	necdin
1435494_s_at	Dsp	desmoplakin
1435655_at	Rpl12	ribosomal protein L12
1435989_x_at	Krt8	keratin 8

1436362_x_at	100043775 /// 2700079J08Rik /// Ccrn4l	predicted gene, 100043775 /// RIKEN cDNA 2700079J08 gene /// CCR4 carbon catabolite repression 4-like (S. cerevisiae)
1436399_s_at	Nrk	Nik related kinase
1436506_a_at	Snhg6	small nucleolar RNA host gene (non-protein coding) 6
1436567_a_at	Ndufa7	NADH dehydrogenase (ubiquinone) 1 alpha subcomplex, 7 (B14.5a)
1436689_a_at	Aldh9a1	aldehyde dehydrogenase 9, subfamily A1
1436714_at	Lpp	LIM domain containing preferred translocation partner in lipoma
1436715_s_at	Cdipt	CDP-diacylglycerol--inositol 3-phosphatidyltransferase (phosphatidylinositol synthase)
1436746_at	Wnk1	WNK lysine deficient protein kinase 1
1436775_a_at	Ankrd17	ankyrin repeat domain 17
1436790_a_at	Sox11	SRY-box containing gene 11
1436879_x_at	Afp	alpha fetoprotein
1436898_at	Sfpq	splicing factor proline/glutamine rich (polypyrimidine tract binding protein associated)
1436915_x_at	Laptm4b	lysosomal-associated protein transmembrane 4B
1436926_at	Esrrb	estrogen related receptor, beta
1436990_s_at	Chchd10	coiled-coil-helix-coiled-coil-helix domain containing 10
1436991_x_at	Gsn	gelsolin
1437171_x_at	Gsn	gelsolin
1437238_x_at	Nmd3	NMD3 homolog (S. cerevisiae)
1437277_x_at	Tgm2	transglutaminase 2, C polypeptide
1437308_s_at	F2r	coagulation factor II (thrombin) receptor
1437331_a_at	Arf3	ADP-ribosylation factor 3
1437340_x_at	Gkn1	gastrokine 1
1437396_at	Creb3l2	cAMP responsive element binding protein 3-like 2
1437458_x_at	Clu	clusterin
1437502_x_at	Cd24a /// EG621324	CD24a antigen /// predicted gene, EG621324
1437689_x_at	Clu	clusterin
1437810_a_at	Hbb-bh1 /// LOC100044263	hemoglobin Z, beta-like embryonic chain /// hypothetical protein LOC100044263
1437974_a_at	Hk1	hexokinase 1
1437990_x_at	Hbb-bh1 /// LOC100044263	hemoglobin Z, beta-like embryonic chain /// hypothetical protein LOC100044263
1438006_at	4933439F18Rik	RIKEN cDNA 4933439F18 gene
1438118_x_at	Vim	vimentin

1438190_x_at	Tpbpa	trophoblast specific protein alpha
1438651_a_at	Aplnr	apelin receptor
1438840_x_at	Apoa1	apolipoprotein A-I
1438932_at	Rasgrp2	RAS, guanyl releasing protein 2
1438941_x_at	Ampd2	adenosine monophosphate deaminase 2 (isoform L)
1439002_s_at	Prl3d1	prolactin family 3, subfamily d, member 1
1439415_x_at	EG546663 /// Rps21	predicted gene, EG546663 /// ribosomal protein S21
1439440_x_at	Twf2	twinfilin, actin-binding protein, homolog 2 (Drosophila)
1439460_a_at	Arfgap2	ADP-ribosylation factor GTPase activating protein 2
1439476_at	Dsg2	desmoglein 2
1448029_at	Tbx3	T-box 3
1448123_s_at	Tgfbi	transforming growth factor, beta induced
1448131_at	Mfn2	mitofusin 2
1448134_at	X99384	cDNA sequence X99384
1448152_at	Igf2	insulin-like growth factor 2
1448182_a_at	Cd24a /// EG621324	CD24a antigen /// predicted gene, EG621324
1448213_at	Anxa1	annexin A1
1448229_s_at	Ccnd2	cyclin D2
1448234_at	Dnajb6	DnaJ (Hsp40) homolog, subfamily B, member 6
1448237_x_at	Ldhb	lactate dehydrogenase B
1448259_at	Fstl1	follistatin-like 1
1448260_at	Uchl1	ubiquitin carboxy-terminal hydrolase L1
1448306_at	Nfkbia	nuclear factor of kappa light polypeptide gene enhancer in B-cells inhibitor, alpha
1448371_at	Mylpf	myosin light chain, phosphorylatable, fast skeletal muscle
1448380_at	Lgals3bp	lectin, galactoside-binding, soluble, 3 binding protein
1448383_at	Mmp14	matrix metallopeptidase 14 (membrane-inserted)
1448392_at	Sparc	secreted acidic cysteine rich glycoprotein
1448402_at	Tln1	talin 1
1448424_at	Frzb	frizzled-related protein
1448432_at	Plcd1	phospholipase C, delta 1
1448438_at	Derl2	Der1-like domain family, member 2
1448469_at	Nid1	nidogen 1
1448572_at	Prl4a1	prolactin family 4, subfamily a, member 1
1448592_at	Crtap	cartilage associated protein

1448607_at	Nampt	nicotinamide phosphoribosyltransferase
1448670_at	LOC100047012 /// Ube2e3	similar to ubiquitin-conjugating enzyme UbcM2 /// ubiquitin-conjugating enzyme E2E 3, UBC4/5 homolog (yeast)
1448705_at	Zbtb22	zinc finger and BTB domain containing 22
1448793_a_at	Sdc4	syndecan 4
1448797_at	Elk3	ELK3, member of ETS oncogene family
1448889_at	Slc38a4	solute carrier family 38, member 4
1448964_at	S100g	S100 calcium binding protein G
1448984_at	Ercc4	excision repair cross-complementing rodent repair deficiency, complementation group 4
1449024_a_at	Hexa	hexosaminidase A
1449032_at	Prl2a1	prolactin family 2, subfamily a, member 1
1449106_at	Gpx3	glutathione peroxidase 3
1449283_a_at	Mapk12	mitogen-activated protein kinase 12
1449410_a_at	Gas5	growth arrest specific 5
1449529_s_at	Prl7a1	prolactin family 7, subfamily a, member 1
1449534_at	Sycp3	synaptonemal complex protein 3
1449540_at	Rhox9	reproductive homeobox 9
1449731_s_at	Nfkbia	nuclear factor of kappa light polypeptide gene enhancer in B-cells inhibitor, alpha
1449888_at	Epas1 /// LOC100048537	endothelial PAS domain protein 1 /// similar to Endothelial PAS domain protein 1
1449939_s_at	Dlk1	delta-like 1 homolog (Drosophila)
1450021_at	Ubqln2	ubiquilin 2
1450032_at	Slco2a1	solute carrier organic anion transporter family, member 2a1
1450078_at	Nrk	Nik related kinase
1450079_at	Nrk	Nik related kinase
1450095_a_at	Acyp1	acylphosphatase 1, erythrocyte (common) type
1450157_a_at	Hmmr	hyaluronan mediated motility receptor (RHAMM)
1450333_a_at	Gata2	GATA binding protein 2
1450429_at	Capn6	calpain 6
1450461_at	Tcf7	transcription factor 7, T-cell specific
1450506_a_at	Aen	apoptosis enhancing nuclease
1450641_at	Vim	vimentin
1450731_s_at	Tnfrsf21	tumor necrosis factor receptor superfamily, member 21
1450757_at	Cdh11	cadherin 11
1450780_s_at	Hmga2	high mobility group AT-hook 2

1450781_at	Hmga2	high mobility group AT-hook 2
1450843_a_at	Serpinh1	serine (or cysteine) peptidase inhibitor, clade H, member 1
1450851_at	Wdr1	WD repeat domain 1
1450852_s_at	F2r	coagulation factor II (thrombin) receptor
1450878_at	Sri	sorcin
1450894_a_at	Ap2m1	adaptor protein complex AP-2, mu1
1450904_at	Tmem167	transmembrane protein 167
1450929_at	Zfp57	zinc finger protein 57
1450934_at	Eif4a2	eukaryotic translation initiation factor 4A2
1450947_at	2610528J11Rik	RIKEN cDNA 2610528J11 gene
1450976_at	Ndrg1	N-myc downstream regulated gene 1
1450997_at	Stk17b	serine/threonine kinase 17b (apoptosis-inducing)
1451069_at	Pim3	proviral integration site 3
1451102_at	Cnot8	CCR4-NOT transcription complex, subunit 8
1451127_at	AW146242	expressed sequence AW146242
1451190_a_at	Sbk1	SH3-binding kinase 1
1451241_at	Lamb1-1	laminin B1 subunit 1
1451335_at	Plac8	placenta-specific 8
1451580_a_at	Ttr	transthyretin
1451683_x_at	H2-D1	histocompatibility 2, D region locus 1
1451695_a_at	Gpx4	glutathione peroxidase 4
1451701_x_at	Cldn3	claudin 3
1451771_at	Tpcn1	two pore channel 1
1451784_x_at	H2-D1	histocompatibility 2, D region locus 1
1451790_a_at	Tfpi	tissue factor pathway inhibitor
1451791_at	Tfpi	tissue factor pathway inhibitor
1451931_x_at	H2-L	histocompatibility 2, D region
1451987_at	Arrb2	arrestin, beta 2
1452035_at	Col4a1	collagen, type IV, alpha 1
1452092_at	4631426J05Rik	RIKEN cDNA 4631426J05 gene
1452094_at	P4ha1	procollagen-proline, 2-oxoglutarate 4-dioxygenase (proline 4-hydroxylase), alpha 1 polypeptide
1452114_s_at	Igfbp5	insulin-like growth factor binding protein 5
1452141_a_at	Sepp1	selenoprotein P, plasma, 1
1452165_at	Prl2b1	prolactin family 2, subfamily b, member 1
1452181_at	Ckap4	cytoskeleton-associated protein 4
1452203_at	Obfc2a	oligonucleotide/oligosaccharide-binding fold containing 2A
1452207_at	Cited2	Cbp/p300-interacting transactivator, with Glu/Asp-rich carboxy-terminal domain, 2

1452217_at	Ahnak	AHNAK nucleoprotein (desmoyokin)
1452249_at	Prickle1	prickle like 1 (Drosophila)
1452318_a_at	Hspa1b	heat shock protein 1B
1452357_at	Gp1bb /// Sept5	glycoprotein Ib, beta polypeptide /// septin 5
1452381_at	Creb3l2	cAMP responsive element binding protein 3-like 2
1452432_at	Tfpi	tissue factor pathway inhibitor
1452606_at	Mnd1	meiotic nuclear divisions 1 homolog (S. cerevisiae)
1452713_a_at	Wdr57	WD repeat domain 57 (U5 snRNP specific)
1452772_at	Tnks2	tankyrase, TRF1-interacting ankyrin-related ADP-ribose polymerase 2
1452774_at	Hnrnpa3	heterogeneous nuclear ribonucleoprotein A3
1453132_a_at	Gkn2	gastrokine 2
1453223_s_at	Dppa2	developmental pluripotency associated 2
1453988_a_at	Ide	insulin degrading enzyme
1453993_a_at	Bnip2	BCL2/adenovirus E1B interacting protein 2
1454159_a_at	Igfbp2	insulin-like growth factor binding protein 2
1454247_a_at	Gpa33	glycoprotein A33 (transmembrane)
1454268_a_at	Cyba	cytochrome b-245, alpha polypeptide
1454608_x_at	Ttr	transthyretin
1454763_at	Ankrd17	ankyrin repeat domain 17
1454838_s_at	AW548124 /// LOC100048505	expressed sequence AW548124 /// similar to Expressed sequence AW548124
1454849_x_at	Clu	clusterin
1454862_at	Phldb2	pleckstrin homology-like domain, family B, member 2
1454984_at	Lifr	leukemia inhibitory factor receptor
1455019_x_at	Ckap4	cytoskeleton-associated protein 4
1455056_at	Lmo7	LIM domain only 7
1455061_a_at	Acaa2	acetyl-Coenzyme A acyltransferase 2 (mitochondrial 3-oxoacyl-Coenzyme A thiolase)
1455195_at	EG383815 /// EG547267 /// EG668041 /// LOC677113 /// Rps24	predicted gene, EG383815 /// predicted gene, EG547267 /// predicted gene, EG668041 /// similar to ribosomal protein S24 /// ribosomal protein S24
1455201_x_at	Apoa1	apolipoprotein A-I
1455214_at	Mitf	microphthalmia-associated transcription factor
1455235_x_at	Ldhb	lactate dehydrogenase B
1455288_at	1110036O03Rik	RIKEN cDNA 1110036O03 gene

1455316_x_at	ENSMUSG00000073624	predicted gene, ENSMUSG00000073624
1455792_x_at	Ndn	necdin
1455871_s_at	OTTMUSG00000012893 /// Rpl13 /// Tax1bp3	predicted gene, OTTMUSG00000012893 /// ribosomal protein L13 /// Tax1 (human T-cell leukemia virus type I) binding protein 3
1455913_x_at	Ttr	transthyretin
1455956_x_at	Ccnd2	cyclin D2
1456042_s_at	Cramp1l	Crm, cramped-like (Drosophila)
1456174_x_at	Ndrg1	N-myc downstream regulated gene 1
1456292_a_at	Vim	vimentin
1456312_x_at	Gsn	gelsolin
1456388_at	Atp11a	ATPase, class VI, type 11A
1456424_s_at	Pltp	phospholipid transfer protein
1456466_x_at	Atxn10	ataxin 10
1456615_a_at	Bptf	bromodomain PHD finger transcription factor
1456733_x_at	Serpinh1	serine (or cysteine) peptidase inhibitor, clade H, member 1
1460217_at	Prl7d1	prolactin family 7, subfamily d, member 1
1460319_at	Fut8	fucosyltransferase 8
1460330_at	Anxa3	annexin A3
1460356_at	Esam	endothelial cell-specific adhesion molecule
1460605_at	Crxos1	Crx opposite strand transcript 1
1460700_at	Stat3	signal transducer and activator of transcription 3
